# Analysis of Differentially Expressed Genes in Coronary Artery Disease by Integrated Microarray Analysis

**DOI:** 10.3390/biom10010035

**Published:** 2019-12-25

**Authors:** Meenashi Vanathi Balashanmugam, Thippeswamy Boreddy Shivanandappa, Sivagurunathan Nagarethinam, Basavaraj Vastrad, Chanabasayya Vastrad

**Affiliations:** 1 Department of Biomedical Sciences, College of Pharmacy, Shaqra University, Al Dawadmi 11911, Saudi Arabia; meenashivanathi@gmail.com (M.V.B.); t_swamy@hotmail.com (T.B.S.); sivagurunathann@gmail.com (S.N.); 2 Department of Pharmaceutics, SET’S College of Pharmacy, Dharwad, Karnataka 580002, India; basavarajmv@gmail.com; 3 Biostatistics and Bioinformatics, Chanabasava Nilaya, Bharthinagar, Dharwad 580001, Karanataka

**Keywords:** coronary artery disease, differentially expressed genes, hub genes, protein-protein interaction network, pathway enrichment analyses

## Abstract

Coronary artery disease (CAD) is a major cause of end-stage cardiac disease. Although profound efforts have been made to illuminate the pathogenesis, the molecular mechanisms of CAD remain to be analyzed. To identify the candidate genes in the advancement of CAD, microarray dataset GSE23766 was downloaded from the Gene Expression Omnibus database. The differentially expressed genes (DEGs) were identified, and pathway and gene ontology (GO) enrichment analyses were performed. The protein-protein interaction network was constructed and the module analysis was performed using the Biological General Repository for Interaction Datasets (BioGRID) and Cytoscape. Additionally, target genes-miRNA regulatory network and target genes-TF regulatory network were constructed and analyzed. There were 894 DEGs between male human CAD samples and female human CAD samples, including 456 up regulated genes and 438 down regulated genes. Pathway enrichment analyses revealed that DEGs (up and down regulated) were mostly enriched in the superpathway of steroid hormone biosynthesis, ABC transporters, oxidative ethanol degradation III and Complement and coagulation cascades. Similarly, geneontology enrichment analyses revealed that DEGs (up and down regulated) were mostly enriched in the forebrain neuron differentiation, filopodium membrane, platelet degranulation and blood microparticle. In the PPI network and modules (up and down regulated), MYC, NPM1, TRPC7, UBC, FN1, HEMK1, IFT74 and VHL were hub genes. In the target genes-miRNA regulatory network and target genes—TF regulatory network (up and down regulated), TAOK1, KHSRP, HSD17B11 and PAH were target genes. In conclusion, the pathway and GO ontology enriched by DEGs may reveal the molecular mechanism of CAD. Its hub and target genes, MYC, NPM1, TRPC7, UBC, FN1, HEMK1, IFT74, VHL, TAOK1, KHSRP, HSD17B11 and PAH were expected to be new targets for CAD. Our finding provided clues for exploring molecular mechanism and developing new prognostics, diagnostic and therapeutic strategies for CAD.

## 1. Introduction

Coronary artery disease (CAD), the most common type of heart disease, is considered a complicated disease. CAD is caused due to narrowing or blockage of the coronary arteries due to buildup of cholesterol and fatty deposits on the inner walls of the arteries [1]. The causes of CAD consist of genetic risk and environmental influence [2,3,4]. Compared with histological classification, genes play many key roles in the diagnosis, treatment, and prognosis of CAD [5]. Alteration in genes such as ABCA1 [6] and human paraoxonase/arylesterase (HUMPONA) [7] were associated with development of CAD. Polymorphism in genes such as β fibrinogen [8] apolipoprotein A-I [9] heme oxygenase-1 [10], PON1 and PON2 [11], PAI-1 [12], MMP-2, MMP-3, MMP-9 and MMP-12 [13], NADH/NADPH oxidase [14] and angiotensin II type 1 receptor [15] were responsible for the progression of CAD. To date, the precise molecular mechanisms of CAD are still unknown, it is extremely important to uncover the underlying genes contributed to CAD.

With the rapid development of microarray technology, some high throughput platforms for analysis of gene expression are widely used to explore the differentially expressed genes (DEGs) during progression of cardiac diseases and molecular mechanisms of cardiovascular drugs [16,17]. However, the DEGs diagnosed with microarray dependon the sample size, gender, grading, and ethnic group, etc. The genes obtained from microarrays might be more representative.

In the current study, messenger RNA (mRNA) microarray datasets (GSE23766) were downloaded from the Gene Expression Omnibus (GEO) database, which were subsequently analyzed to obtain overlapping DEGs. Pathway and gene ontology (GO) enrichment analysis and PPI network construction, module analysis, target genes-miRNA regulatory network construction and target genes-TF regulatory network construction were applied to diagnose the important genes linked with CAD.

## 2. Materials and Methods

### 2.1. Microarray Data and Data Processing

**Figure 1:**
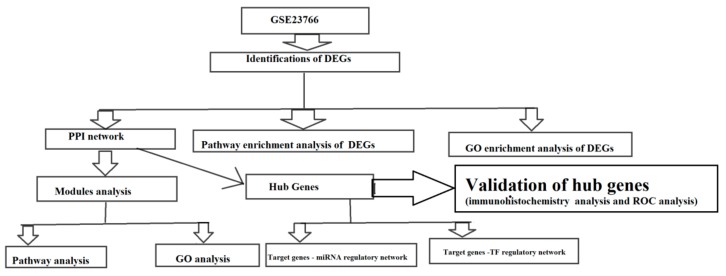
Study design (flow diagram of study).

Flowchart of materials and methods is shown in Figure 1. The gene expression profile GSE23766 was downloaded from the GEO database (http://www.ncbi.nlm.nih.gov/geo/), which were all based on GPL6480 Agilent-014850 Whole Human Genome Microarray 4 × 44K G4112F (AGILENT TECHNOLOGIES, INC, 5301 Stevens Creek Blvd, Santa Clara, CA 95051, USA). The GSE23766 dataset contained 16 male human CAD samples and 16 female human CAD samples. Series Matrix text files of the dataset were obtained. Subsequently, background correction, quartile normalization and probe summarization were performed with the limma R package [18,19].

### 2.2. Identification of DEGs between Male CAD and Female CAD Samples

In this study, we used an empirical Bayes t-test (eBayes) to identify the DEGs between male human CAD samples and female human CAD samples with cutoff value |log2(Fold Change)| > 0.1694 for up regulated genes, |log2(Fold Change)| > 0.229 for down regulated genes and adjusted *p*-value < 0.05.

### 2.3. Pathway Enrichment Analysis of DEGs

The pathway enrichment of candidate DEGs were analyzed using multiple online databases. ToppGene (ToppFun) (https://toppgene.cchmc.org/enrichment.jsp) [20] is a website for gene annotation and visualization with an integrated discovery function and can, therefore, provide the biological meaning of genes, including gene annotations and visualization. Pathway enrichment analysis of DEGs was carried out using the Kyoto Encyclopedia of Genes and Genomes (KEGG; http://www.genome.jp/kegg/) [21], Pathway Interaction Database (PID, http://pid.nci.nih.gov/) [22], Reactome (https://reactome.org/PathwayBrowser/) [23], Molecular signatures database (MSigDB, http://software.broadinstitute.org/gsea/msigdb/) [24], GenMAPP (http://www.genmapp.org/) [25], Pathway Ontology (https://bioportal.bioontology.org/ontologies/PW) [26] and PantherDB (http://www.pantherdb.org/) [27] websites, with *p* < 0.05 as the cutoff value.

### 2.4. GO Enrichment Analysis of DEGs

GO (http://www.geneontology.org/) [28] analysis is a common genes analysis method, which can contribute functional classification for genomic data, with categories of biological processes (BP), cellular component (CC), and molecular function (MF). ToppGene (ToppFun) (https://toppgene.cchmc.org/enrichment.jsp) [20] is an online tool for gene functional classification, which can systematic and integrative analysis of large gene lists.

### 2.5. Comprehensive Analysis of PPI Network and Modules

We used Biological General Repository for Interaction Datasets (BioGRID) (https://thebiogrid.org/) to assess protein–protein interaction (PPI) information [29]. In addition, in order to explore the relationship between DEGs, we used the BioGRID which is integrated with various protein interaction database partners such as Molecular INTeraction Database (MINT, https://mint.bio.uniroma2.it/) [30], IntAct (https://www.ebi.ac.uk/intact/) [31], Database of Interacting Proteins (DIP, https://dip.doe-mbi.ucla.edu/dip/Main.cgi) [32], Pathway Commons (http://www.pathwaycommons.org/) [33], iRefIndex (http://irefindex.org/wiki/index.php?title=iRefIndex) [34], STRING (https://string-db.org/) [35], MatrixDB (http://matrixdb.univ-lyon1.fr/) [36], MPIDB (https://www.jcvi.org/mpidb/about.php) [37], InnateDB (https://www.innatedb.com/) [38], iRefWeb (http://wodaklab.org/iRefWeb/search/index) [39], I2D (http://ophid.utoronto.ca/ophidv2.204/) [40] and converted the results visually by using Cytoscape software (http://www.cytoscape.org/) [41]. Topological properties of PPI network such as node degree [42], betweenness [43], stress [44], closeness [45] and clustering coefficient [46] were calculated. Furthermore, module analysis was performed by using the PEWCC1 [47] plugin (version 1.3) to explore the most important clustering modules in the huge PPI network (degree cutoff = 5, k-core = 2, node score cutoff = 0.2, and max. Depth rom Seed: 100).

### 2.6. Construction of Target Genes-miRNA Regulatory Network

The miRNAs of target genes were predicted by two established miRNA target prediction databases such as DIANA-TarBase (http://diana.imis.athena-innovation.gr/DianaTools/index.php?r=tarbase/index) [48] and miRTarBase (http://mirtarbase.mbc.nctu.edu.tw/php/download.php) [49]. The miRNAs predicted by online tool NetworkAnalyst (https://www.networkanalyst.ca/) [50] were selected as the miRNAs of target genes. A network based on correlation analysis of target genes and miRNAs linked with CAD was constructed by Cytoscape software (http://www.cytoscape.org/, version 3.7.2, National Institutes of Health, Bethesda, MD, USA) [41]. In the network, a green (up regulated) and red (down regulated) circular node represented the target genes and a white and blue diamond shape node represented the miRNA, their interaction was represented by a line. The numbers of lines in the networks indicated the contribution of one miRNA to the surrounding target genes, and the higher the degree, the more central the target gene was within the network.

### 2.7. Construction of Target Genes-TF Regulatory Network

The transcription factors (TFs) of target genes were predicted by established TF target prediction databases such as JASPAR (http://jaspar.genereg.net/) [51]. The miRNAs predicted by online tool Network Analyst (https://www.networkanalyst.ca/) [50] were selected as the TFs of target genes. A network based on correlation analysis of target genes and TFs linked with CAD was constructed by Cytoscape software (http://www.cytoscape.org/) [41]. In the network, a green (up regulated) and red (down regulated) circular node represented the target genes and a gray and blue triangle shape node represented the TF, their interaction was represented by a line. The numbers of lines in the networks indicated the contribution of one TF to the surrounding target genes, and the higher the degree, the more central the target gene was within the network.

### 2.8. Hub Gene Expression Levels in CAD

The Human Protein Atlas (HPA) (https://www.proteinatlas.org/) was used to validate the expression level of the hub genes [52].

### 2.9. Receiver Operating Characteristic Curve Analysis

Receiver operating characteristic curve (ROC) analysis was performed using R package “pROC” [53] to distinguish male CAD samples from female CAD tissues. In GSE23766, we worked out the AUC to distinguish male CAD samples from female CAD tissues. After that, we compared the expression levels of candidate hub genes in male CAD and female CAD tissues using GSE23766.

## 3. Results

### 3.1. Identification of DEGs

**Figure 2:**
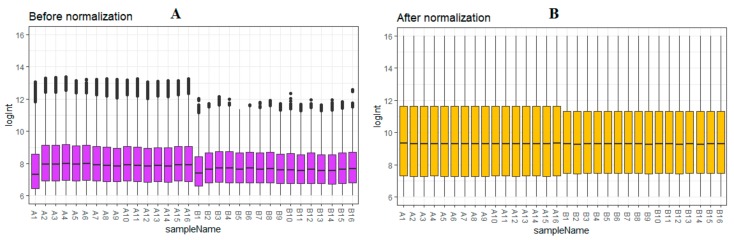
Box plots of the gene expression data before (**A**) and after normalization (**B**). Horizontal axis represents the sample symbol and the vertical axis represents the gene expression values. The black line in the box plot represents the median value of gene expression. (A1–A16 = male human CAD samples; B1–B16 = female human CAD samples).

**Figure 3:**
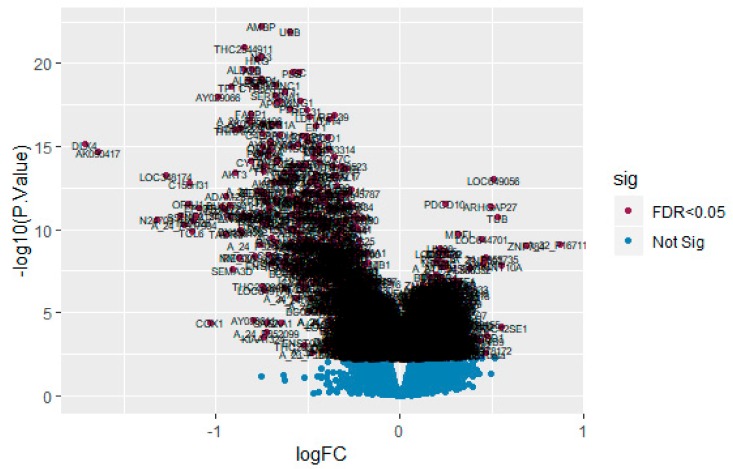
Volcano plot of differentially expressed genes. Genes with a significant change of more than two-fold were selected.

**Figure 4:**
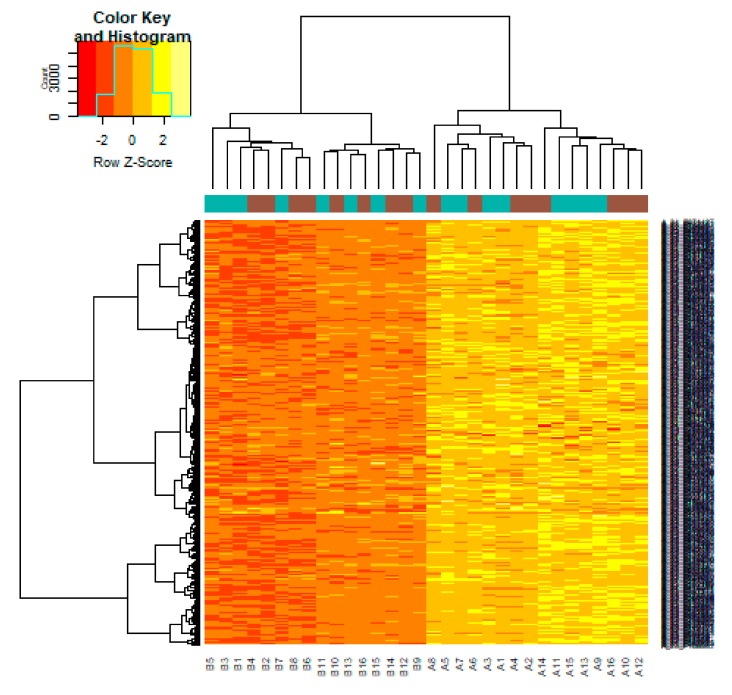
Heat map of up regulated differentially expressed genes. Legend on the top left indicate log fold change of genes. (A1, A2, A3, A4, A5, A6, A7, A8, A9, A10, A11, A12, A13, A14, A15, A16 = male human CAD samples; B1, B2, B3, B4, B5, B6, B7, B8, B9, B10, B11, B12, B13, B14, B15, B16 = female human CAD samples).

**Figure 5:**
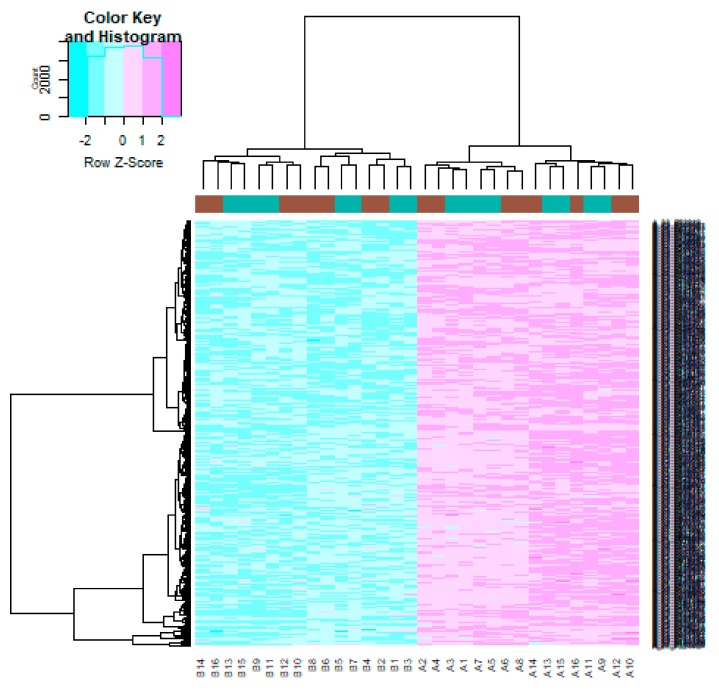
Heat map of down regulated differentially expressed genes. Legend on the top left indicate log fold change of genes. (A1, A2, A3, A4, A5, A6, A7, A8, A9, A10, A11, A12, A13, A14, A15, A16 = male human CAD samples; B1, B2, B3, B4, B5, B6, B7, B8, B9, B10, B11, B12, B13, B14, B15, B16 = female human CAD samples).

After quality control, normalization and batch effect adjustment, gene expression profiles from CAD samples with male and female were compared. The boxplot proved good normalization of the GSE23766 data (Figure 2A,B). A total of 894 significant DEGs (adjusted *p*-value < 0.05, |log2(Fold Change)| > 0.1694 for up regulated genes, |log2(Fold Change)| > 0.229 for down regulated genes), including 456 up regulated and 438 down regulated genes, were identified from GSE23766 dataset (Table S1). The volcano plot of each gene expression profile data was shown in Figure 3. Hierarchical clustering analysis showed the expression pattern of DEGs among samples, which suggested that the expression of genes in CAD with male significantly differ from those in adjacent CAD with female (Figure 4 and Figure 5).

### 3.2. Pathway Enrichment Analysis

To gain further insight into the identified DEGs (up and down regulated genes), pathway and GO enrichment analyses were conducted using ToppGene and are given in Tables S2 and S3. Pathway enrichment analysis showed that up regulated genes were mainly involved in PABC transporters, BARD1 signaling events, androgen/estrogene/progesterone biosynthesis and multidrug resistance-associated protein mediated transport, while down regulated genes were mainly involved to complement and coagulation cascades, FOXA2 and FOXA3 transcription factor networks, fatty acid metabolism and fibrinolysis pathway. Gene ontology (GO) enrichment analysis showed that up regulated genes were mainly associated in biological processes (BP), cellular component (CC) and molecular function (MF), including forebrain neuron differentiation, filopodium membrane and DNA binding, bending, while down regulated genes were mainly associated in biological processes (BP), cellular component (CC) and molecular function (MF), including platelet degranulation, blood microparticle and peptidase regulator activity.

### 3.3. GO Enrichment Analysis of DEGs

Enrichment analyses for the up regulated and down regulated genes were performed by ToppGene. The up regulated genes were mainly enriched in forebrain neuron differentiation and negative regulation of immune system process by BP, filopodium membrane and nucleolus by CC and DNA binding, bending and protein dimerization activity by MF, respectively (Table S4). Meanwhile, down regulated genes were mainly enriched in platelet degranulation and response to inorganic substance by BP, blood microparticle and extracellular space by CC and peptidase regulator activity and endopeptidase inhibitor activity by MF, respectively (Table S5).

### 3.4. Comprehensive Analysis of PPI Network and Modules

**Figure 6:**
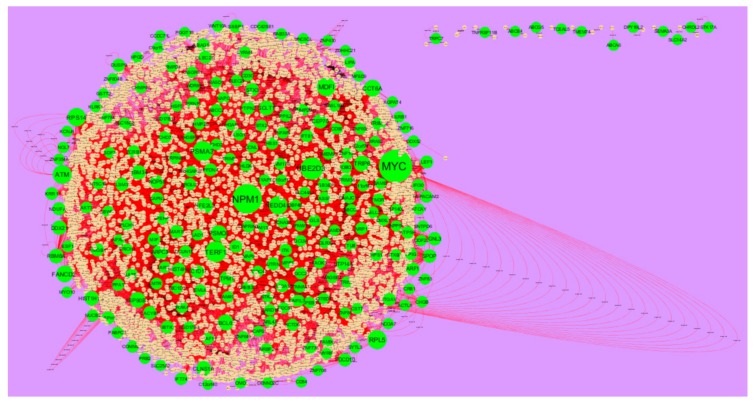
Protein–protein interaction network of up regulated genes. Green nodes denotes up regulated genes.

**Figure 7:**
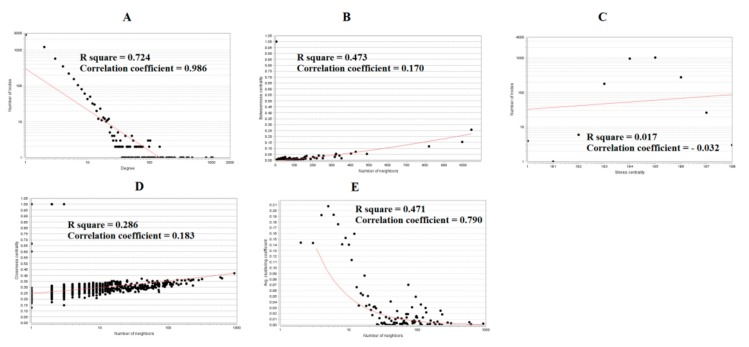
Regression diagrams for up regulated genes (**A**) Node degree; (**B**) Betweenness centrality; (**C**) Stress centrality; (**D**) Closeness centrality; (**E**) Clustering coefficient.

**Figure 8:**
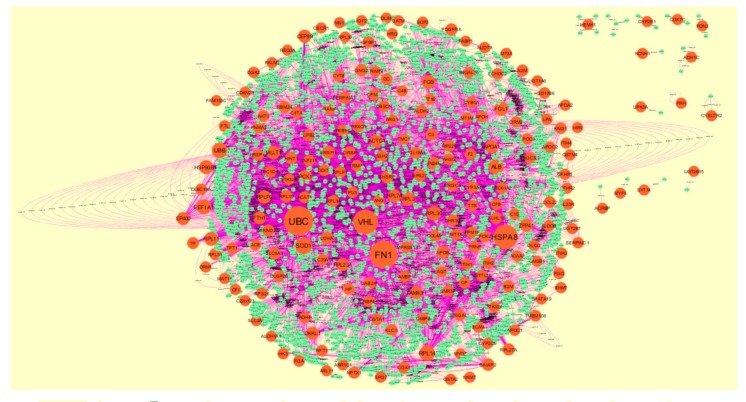
Protein–protein interaction network of down regulated genes. Orange nodes denotes down regulated genes.

**Figure 9:**
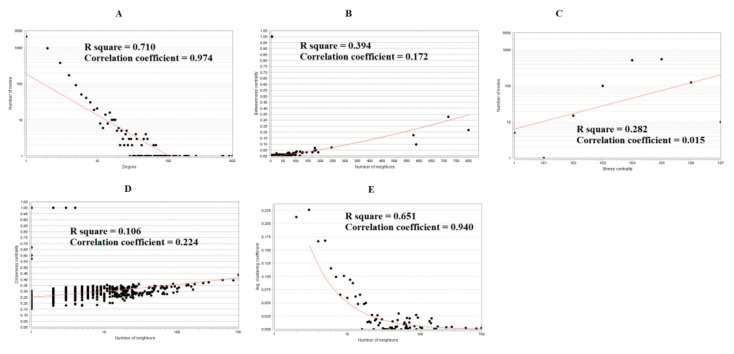
Regression diagrams for down regulated genes (**A**) Node degree; (**B**) Betweenness centrality; (**C**) Stress centrality; (**D**) Closeness centrality; (**E**) Clustering coefficient.

The PPI network of up regulated genes consisted of 5840 nodes and 16,142 edges (Figure 6). Hub genes with high node degree such as MYC (degree = 998), NPM1 (degree = 820), UBE2D3 (degree = 488), TERF1 (degree = 429) and PSMA3 (degree = 403) were listed in Table S6. R square = 0.724 and correlation coefficient = 0.986 for node degree (Figure 7A). Hub genes with high betweenness such as MYC (betweenness = 0.15430897), NPM1 (betweenness = 0.11756148), TERF1 (betweenness = 0.06992938), PSMA3 (betweenness = 0.05585022) and MDFI (betweenness = 0.055025) were listed in Table S6. R square = 0.473 and correlation coefficient = 0.170 for betweenness (Figure 7B). Hub genes with high stress such as MYC (stress = 168499346), NPM1 (stress = 107362468), PSMA3 (stress = 49381886), MDFI (stress = 39487556) and TERF1 (stress = 32105062) were listed in Table S6. R square = 0.017 and correlation coefficient = −0.032 for stress (Figure 7C). Hub genes with high closeness such as NPM1 (closeness = 0.38413706), MYC (closeness = 0.37588193), TERF1 (closeness = 0.36795083), CCT6A (closeness = 0.36512827) and RPS14 (closeness = 0.36450945) were listed in Table S6. R square = 0.286 and correlation coefficient = 0.183 for closeness (Figure 7D). Hub genes with low clustering coefficient such as TRPC7 (clustering coefficient = 0), TNFRSF11B (clustering coefficient = 0), ABCB4 (clustering coefficient = 0), DPY19L2 (clustering coefficient = 0) and ABCG5 (clustering coefficient = 0) were listed in Table S6. R square = 0.471 and correlation coefficient = 0.790 for clustering coefficient (Figure 7E). The PPI network of down regulated genes consisted of 4014 nodes and 8815 edges (Figure 8). Hub genes with high node degree such as UBC (degree = 799), FN1 (degree = 718), VHL (degree = 587), HSPA8 (degree = 576) and SOD1 (degree = 245) were listed in Table S6. R square = 0.710 and correlation coefficient = 0.974 for node degree (Figure 9A). Hub genes with high betweenness such as FN1 (betweenness = 0.32861508), UBC (betweenness = 0.21817265), HSPA8 (betweenness = 0.17366877), VHL (betweenness = 0.09640801) and SOD1 (betweenness = 0.0712639) were listed in Table S6. R square = 0.394 and correlation coefficient = 0.172 for betweenness (Figure 9B). Hub genes with high stress such as UBC (stress = 75534956), FN1 (stress = 48390654), HSPA8 (stress = 48349874), VHL (stress = 18147816) and SOD1 (stress = 17465716) were listed in Table S6. R square = 0.282 and correlation coefficient = 0.015 for stress (Figure 9C). Hub genes with high closeness such as FN1 (closeness = 0.437081), HSPA8 (closeness = 0.39532803), UBC (closeness = 0.39232515), VHL (closeness = 0.37550751) and SOD1 (closeness = 0.36971275) were listed in Table S6. R square = 0.106 and correlation coefficient = 0.224 for closeness (Figure 9D). Hub genes with low clustering coefficient such as HEMK1 (clustering coefficient = 0), ADH1C (clustering coefficient = 0), CYSLTR2 (clustering coefficient = 0), CYP2E1 (clustering coefficient = 0) and COX7C (clustering coefficient = 0) were listed in Table S6. R square = 0.651 and correlation coefficient = 0.940 for clustering coefficient (Figure 9E).

**Figure 10:**
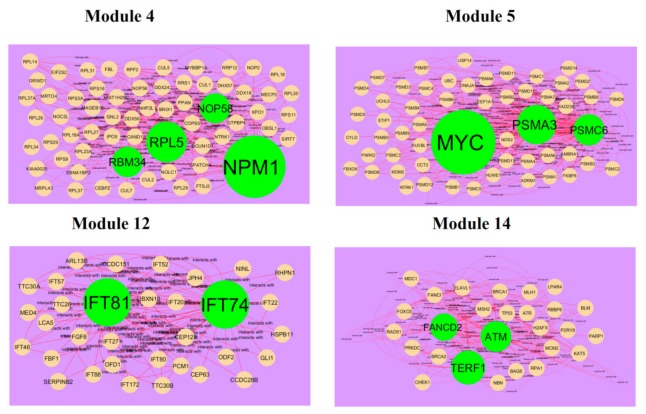
Modules in protein–protein interaction (PPI) network. The green nodes denote the up regulated genes.

**Figure 11:**
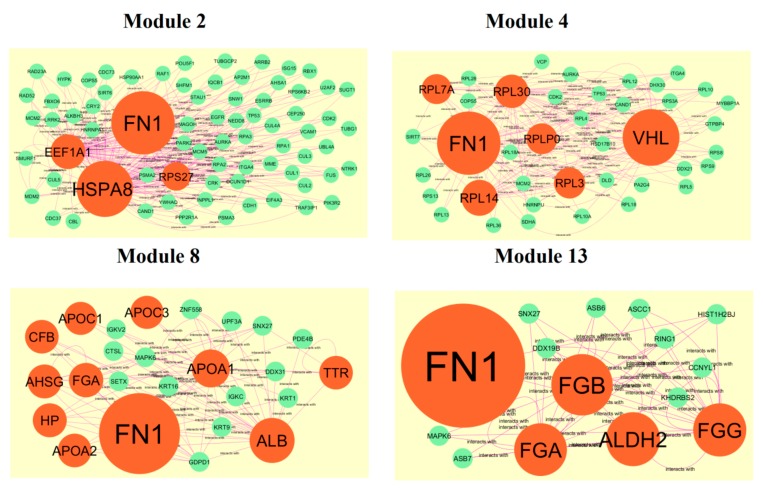
Modules in PPI network. The orange nodes denote the down regulated genes.

After cluster analysis, according to the given parameters, four significant modules (module 4, module 5, module 12 and module 14) were obtained from PPI network for up regulated genes (Figure 10), while four significant modules (module 2, module 4, module 8 and module 13) were obtained from PPI network for down regulated genes (Figure 11). Pathway and GO enrichment analysis showed that up regulated genes in module 4, module 5, module 12 and module 14 were closely related to signaling pathways regulating pluripotency of stem cells, platinum drug resistance, negative regulation of immune system process and protein dimerization activity, while down regulated genes in module 2, module 4, module 8 and module 13 were closely related to amb2 Integrin signaling, hypoxia-inducible factor in the cardiovascular system, platelet degranulation and response to inorganic substance.

### 3.5. Construction of Target Genes-miRNA Regulatory Network

**Figure 12:**
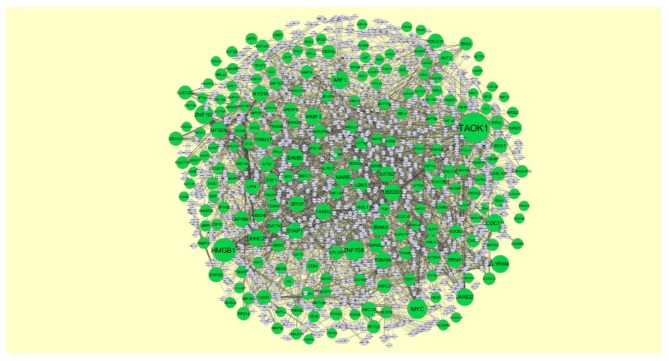
The network of up regulated genes and their related miRNAs. The green circles nodes are the up regulated genes, and white diamond nodes are the miRNAs.

**Figure 13:**
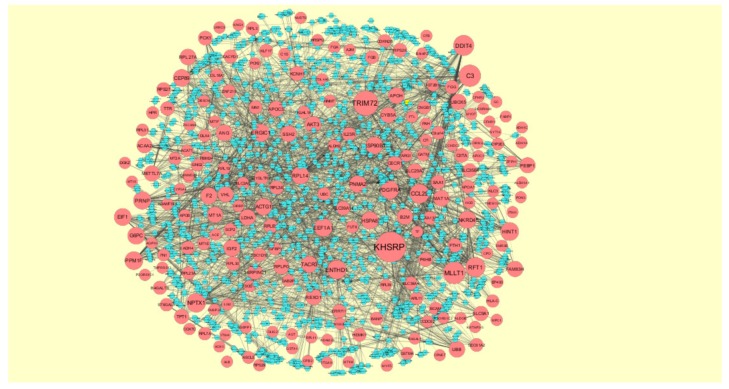
The network of down- regulated genes and their related miRNAs. The red circles nodes are the down regulated genes, and blue diamond nodes are the miRNAs.

MicroRNAs (miRNAs) expressions were responsible for progression of CAD [54]. The miRNAs that may control the up and down regulated target genes are shown in Figure 12 and Figure 13. Top five up regulated target genes such as TAOK1 interacts with 222 miRNAs (ex, hsa-mir-3941), HMGB1 interacts with 143 miRNAs (ex, hsa-mir-1183), ZNF708 interacts with 104 miRNAs (ex, hsa-mir-6832-5p), MYC interacts with 103 miRNAs (ex, hsa-mir-4662b) and ZNF101 interacts with 91 miRNAs (ex, hsa-mir-3187-3p) are listed in Table S7. Top five down regulated target genes such as KHSRP interacts with 178 miRNAs (ex, hsa-mir-548ac), TRIM72 interacts with 123 miRNAs (ex, hsa-mir-6890-3p), MLLT1 interacts with 95 miRNAs (ex, hsa-mir-5681a), C3 interacts with 93 miRNAs (ex, hsa-mir-3135b) and DDIT4 interacts with 82 miRNAs (ex, hsa-mir-3607-3p) are listed in Table S7.

### 3.6. Construction of Target Genes–TF Regulatory Network

**Figure 14:**
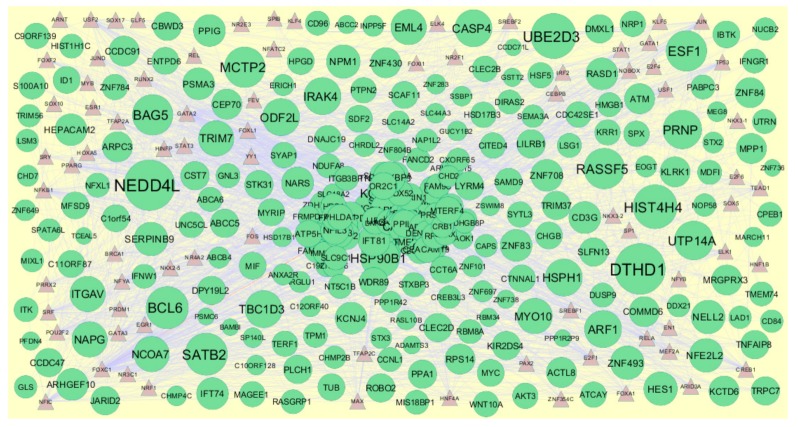
The network of up regulated genes and their related TFs. The green circles nodes are the up regulated genes, and brown triangle nodes are the TFs.

**Figure 15:**
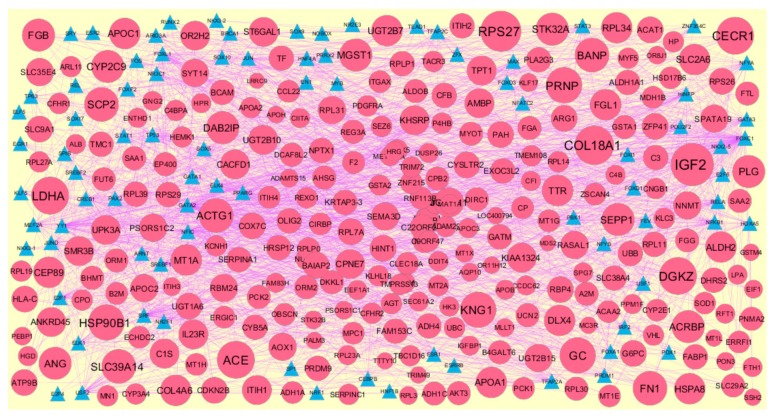
The network of down regulated genes and their related TFs. The green circles nodes are the down regulated genes, and blue triangle nodes are the TFs.

Transcription factors (TFs) expressions were responsible for progression of CAD [55]. The TFs that may control the up and down regulated target genes are shown in Figure 14 and Figure 15. Top five up regulated target genes such as HSD17B11 interacts with 166 TFs (ex, FOXC1), HEPACAM2 interacts with 128 TFs (ex, GATA2), CACYBP interacts with 88 TFs (ex, FOXL1), NAPG interacts with 76 TFs (ex, YY1) and PSMA3 interacts with 59 TFs (ex, USF2) are listed in Table S8. Top five down regulated target genes such as PAH interacts with 149 TFs (ex, FOXC1), CYP3A4 interacts with 118 TFs (ex, GATA2), CP interacts with 76 TFs (ex, YY1), COX7C interacts with 66 TFs (ex, SRF) and TTTY10 interacts with 59 TFs (ex, FOXL1) are listed in Table S8.

### 3.7. Validation of Hub Genes by Immunohistochemistry from HPA Database and Receiver Operating Characteristic Curve analysis

**Figure 16:**
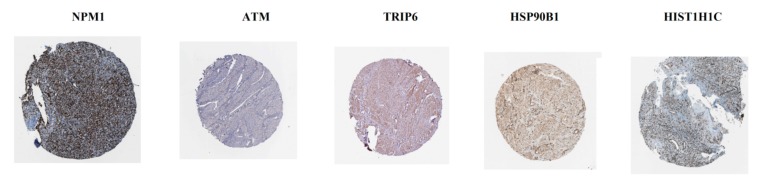
Validation of the hub genes (up regulated) with immunohistochemistry from HPA database. Hub genes NPM1, ATM, TRIP6, HSP90B1 and HIST1H1C were high expressed in CAD.

**Figure 17:**
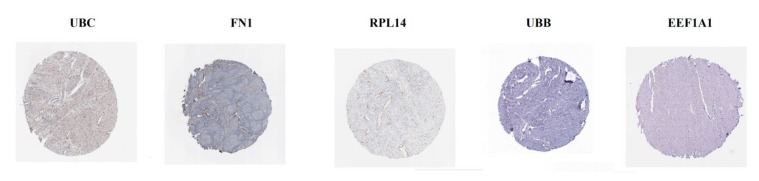
Validation of the hub genes (up regulated) with immunohistochemistry from HPA database. Hub genes UBC, FN1, RPL14, UBB and EEF1A1 were low expressed in CAD.

**Figure 18:**
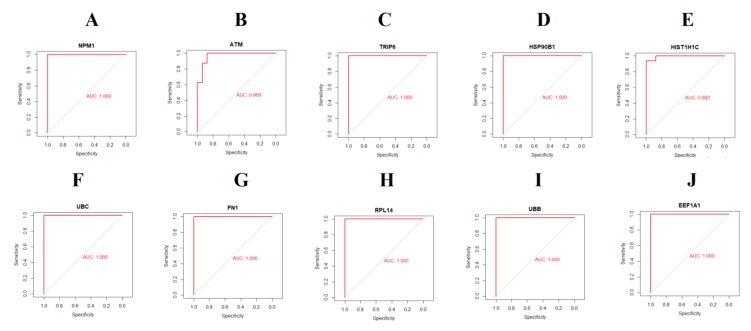
Receiver operating characteristic (ROC) curves and area under the curve (AUC) statistics to evaluate the diagnostic efficiency of the hub genes in GSE23766. (**A**) NPM1 (**B**) ATM (**C**) TRIP6 (**D**) HSP90B1 (**E**) HIST1H1C (**F**) UBC (**G**) FN1 (**H**) RPL14 (**I**) UBB (**J**) EEF1A1.

The analysis from The Human Protein Atlas indicated that the expression of hub genes (up regulated) such as NPM1, ATM, TRIP6, HSP90B1 and HIST1H1C are enhanced in CAD smooth muscle tissue (Figure 16), whereas, the expression of hub genes (down regulated) such as UBC, FN1, RPL14, UBB and EEF1A1 are reduced in CAD smooth muscle tissue (Figure 17). The results of ROC curve indicated that NPM1, TRIP6, HSP90B1, UBC, FN1, RPL14, UBB and EEF1A1 could distinguish male CAD samples from female CAD tissues best, among all the up and down regulated hub genes (NPM1: AUC = 1; ATM: AUC = 0.875; TRIP6: AUC = 1; HSP90B1: AUC = 1, HIST1H1C: AUC = 0.938; UBC: AUC = 1; FN1: AUC = 1; RPL14: AUC = 1; UBB: AUC = 1; EEF1A1: AUC = 1) (Figure 18).

## 4. Discussion

The majority of patients with CAD are diagnosed at advanced stages and have poor overall survival [56]. However, the molecular mechanisms associated in the development of CAD remain unclear.

In the current study, the raw gene expression data of GSE23766 was obtained from the GEO and a total of 894 important DEGs were identified in male CAD samples compared with female CAD samples, including 456 up regulated genes and 438 down regulated genes. Low expression of LIN28 was associated with progression of cardiac ischaemia [57], but this gene may be linked with development of CAD. DUSP9 was involved in progression of cardiac hypertrophy [58], but this gene may be responsible for progression of CAD. Mutation in PSMC6 was important for progression of type 1 diabetes [59], but variation in this gene may be associated with the development of CAD. Decrease expression of ATP5H was liable for progression of mitochondrial dysfunction in cardiomyocytes [60], but low expression of this gene may be linked with the advancement of CAD. Genes such as IRAK4 [61], albumin (ALB) [62] and plasminogen (PLG) [63] were involved in progression of CAD. Alteration in SEPP1 was important for development of peripheral arterial disease [64], but modification in this gene may be liable for advancement of CAD. Polymorphism in ALDH2 was liable for development of CAD [65]. Modification in SERPINA1 was diagnosed with development of large artery stroke [66], but this altered gene may be liable for progression of CAD.

In pathway enrichment analysis, up regulated genes were enriched in various pathway databases such as superpathway of steroid hormone biosynthesis, ABC transporters, BARD1 signaling events, ABC-family proteins mediated transport, methionine metabolism, telomeres, telomerase, cellular aging, and immortality, androgen/estrogene/progesterone biosynthesis, multidrug resistance-associated protein mediated transport and lysosomal acid lipase deficiency (Wolman Disease). Mutation in HSD17B3 was linked with development of type 2 diabetes [67], but this variant gene may be responsible for progression of CAD. ABCG5 was linked with progression of CAD [68]. Mutation in ataxia telangiectasia mutated (ATM) was responsible for progression of CAD in male [69]. ABCC5 was expressed in heart disease [70], but this gene may be associated with advancement of CAD. Lysosomal acid, cholesterol esterase (LIPA) was important for development of CAD [71]. In these pathways genes such as HSD17B11, ABCB4, ABCC2, ABCA6, FANCD2, UBE2D3, NPM1, PSMA3, methionyl-tRNA synthetase (MARS), 5-methyltetrahydrofolate-homocysteine methyltransferase (MTR), v-myc myelocytomatosis viral oncogene homolog (avian) (MYC) and TERF1 were predicted as novel prognostic or diagnostic biomarkers and new therapeutic target in CAD. Up regulated genes such as ABCB4, ABCC2, ABCG5, ABCC5 and ABCA6 were involved in centralized pathway of ABC transporters was associated with pathogenesis of CAD [72]. Down regulated genes were enriched in various pathway databases such as oxidative ethanol degradation III, complement and coagulation cascades, FOXA2 and FOXA3 transcription factor networks, metallothioneins bind metals, fatty acid metabolism, fibrinolysis pathway, plasminogen activating cascade, altered lipoprotein metabolic and enoxaparin pathway. Polymorphism in fibrinogen alpha chain (FGA) was liable for progression of myocardial infarction [73], but this polymorphic gene may be involved in pathogenesis of CAD. Fibrinogen gamma chain (FGG) was diagnosed with progression of ischemic stroke [74], but this gene may be identified with development of CAD. Increased expression of genes such as CPB2 was liable for advancement of myocardial infarction [75], but elevated expression of this gene may be associated with progression of CAD. Over expression of complement component 3 (C3) was responsible for advancement of CAD in female [76]. Low expression of C4B was culpable for pathogenesis of myocardial infarction [77], but decrease expression of this gene may be involved in progression of CAD. Genes such as APOA1 [78] IGFBP1 [79], ACAT1 [80], apolipoprotein B (APOB) [81] and APOC1 [82] were important for pathogenesis of CAD. Genes such as PCK1 [83] and ALDH1A1 [84] were associated with development of diabetes and obesity, but these genes may be linked with progression of CAD. Polymorphism in genes such as MT1A [85], MT2A [86], CYP2C9 [87], CYP3A4 [88], fibrinogen beta chain (FGB) [89], ADH1C [90], lipoprotein, Lp(a) (LPA) [91] and APOC3 [92] were liable for advancement of CAD. Alteration in APOC2 was involved in progression of hypertriglyceridaemia [93], but this gene may be responsible for the development of CAD. Down regulated genes such as ACAT1, ADH1A, ADH1C, ADH4, ACAA2, ALDH1A1, ALDH2, CYP2C9, CYP2E1, CYP3A4 were involved in centralized pathway of fatty acid metabolism was associated with pathogenesis of CAD [94]. In these pathways genes such as CYP2E1, A2M, coagulation factor II (thrombin) (F2), complement factor I (CFI), SERPINC1, integrin, alpha X (complement component 3 receptor 4 subunit) (ITGAX), complement factor B (CFB), C1S, C4BPA, KNG1, transthyretin (prealbumin, amyloidosis type I) (TTR) aldolase B, fructose-bisphosphate (ALDOB), G6PC, MT1B, MT1E, MT1F, MT1G, MT1H, MT1M, MT1X, ADH1A, ADH4 and ACAA2 were predicted as novel prognostic or diagnostic biomarkers and new therapeutic targets in CAD.

In GO enrichment analysis, up regulated genes were enriched in all GO categories such as forebrain neuron differentiation, filopodium membrane and DNA binding, bending. SEMA3A was responsible for progression of myocardial infarction [95], but this gene may be associated with pathogenesis of CAD. Genes such as HES1 [96] and NRP1 [97] were important for progression of cardiac ischemia, but these genes may be linked with CAD. Loss of utrophin (UTRN) was involved in the advancement of cardiomyopathy [98], but this gene may be liable for progression of CAD. Increased expression of HMGB1 was answerable for progression of CAD [99]. In these GO categories, genes such as PHLDA1, SATB2, ROBO2, LEF1, MYO10 and integrin, alpha V (ITGAV) were predicted as novel prognostic or diagnostic biomarkers and new therapeutic target in CAD. Down regulated genes were enriched in all GO categories such as platelet degranulation, blood microparticle and peptidase regulator activity. Polymorphism in apolipoprotein H (APOH) was linked with progression of hypercholesterolemia [100], but this polymorphic gene may be liable for development of CAD. Genes such as IGF2 [101], ITIH4 [102], haptoglobin (HP) [103] and ceruloplasmin (ferroxidase) (CP) [104] were important for advancement of CAD. High expression of SOD1 was involved in the progression of CAD [105]. ITIH3 was answerable for advancement of myocardial infarction [106], but this gene may be associated with development of CAD. Transferrin (TF) was identified with progression of acute stroke [107], but this gene may be linked with development of CAD. Loss of CFHR1 was important for progression of hypertension [108], but loss this gene may be responsible for advancement of CAD. Polymorphism in genes such as angiotensinogen (AGT) [109] and HSPA8 [110] were important for theadvancement of CAD. In these GO categories genes such as alpha-2-HS-glycoprotein (AHSG), histidine-rich glycoprotein (HRG), FN1, ORM1, ORM2, TMPRSS13, ACTG1, haptoglobin-related protein (HPR), alpha-1-microglobulin/bikunin precursor (AMBP), APOA2, group-specific component (GC), ITIH1, ITIH2, acrosin binding protein (ACRBP) and PEBP1 were predicted as novel prognostic or diagnostic biomarkers and new therapeutic target in CAD.

In PPI network, up regulated genes such as MYC, NPM1, UBE2D3, TERF1, PSMA3, MDFI, CCT6A and RPS14 were identified as hub genes showing the highest node degree, betweenness, stress and closeness. Up regulated genes such as TRPC7, TNFRSF11B, ABCB4, DPY19L2 and ABCG5 were identified as hub genes showing the lowest clustering coefficient. TRPC7 was linked with development of CAD [111]. Polymorphism in TNFRSF11B was identified with development of ischemic stroke [112], but this gene may be important for progression of CAD. In this PPI network genes such as myoD family inhibitor (MDFI), CCT6A, RPS14 and DPY19L2 were predicted as novel prognostic or diagnostic biomarkers and new therapeutic target in CAD. Down regulated genes such as UBC, FN1, VHL, HSPA8 and SOD1 were identified as hub genes showing the highest node degree, betweenness, stress and closeness. Down regulated genes such as HEMK1, ADH1C, CYSLTR2, CYP2E1 and COX7C were identified as hub genes showing the lowest clustering coefficient. In this PPI network genes such as ubiquitin C (UBC), VHL (von Hippel-Lindau tumor suppressor), HEMK1, CYSLTR2 and COX7C were predicted as novel prognostic or diagnostic biomarkers and new therapeutic target in CAD.

In module analysis, up regulated genes such as NPM1, RPL5, NOP58, RBM34, MYC, PSMA3, PSMC6, IFT74 and IFT8 were identified as hub genes showing the highest node degree in all four significant modules. In these modules genes such as RPL5, NOP58, RBM34, IFT74 and IFT8 were predicted as novel prognostic or diagnostic biomarkers and new therapeutic target in CAD. Down regulated genes such as FN1, HSPA8, EEF1A1, RPS27, VHL, RPL7A, RPL30, RPLP0, RPL3, RPL14, ALB, TTR, APOA1, APOC3, APOC1, CFB, AHSG, FGA, HP, APOA2, FGB, FGG, ALDH2 and FGA were identified as hub genes showing the highest node degree in all four significant modules. In these modules genes such as EEF1A1, RPS27, RPL7A, RPL30, RPLP0, RPL3 and RPL14 were predicted as novel prognostic or diagnostic biomarkers and new therapeutic target in CAD.

In target gene-miRNA network, up regulated genes such as TAOK1, HMGB1, ZNF708, MYC and ZNF101 were identified as target genes showing the highest number of integration with miRNAs. In this network gene such as TAOK1, ZNF708 and ZNF101 along with miRNA such as hsa-mir-3941, hsa-mir-1183, hsa-mir-6832-5p, hsa-mir-4662b and hsa-mir-3187-3p were predicted as novel prognostic or diagnostic biomarkers and new therapeutic target in CAD. Whereas, down regulated genes such as KHSRP, TRIM72, MLLT1, C3 and DDIT4 were identified as target genes showing the highest number of integration with miRNAs. Low expression of TRIM72 was responsible for development of cardiovascular diseases [113], but loss of this gene may be linked with progression of CAD. In this network down regulated genes such as KH-type splicing regulatory protein (FUSE binding protein 2) (KHSRP), MLLT1 and DDIT4 along with miRNA such as hsa-mir-548ac, hsa-mir-6890-3p, hsa-mir-5681a, hsa-mir-3135b and hsa-mir-3607-3p were predicted as novel prognostic or diagnostic biomarkers and new therapeutic target in CAD.

In the target gene-TF network, up regulated genes such as HSD17B11, HEPACAM2, CACYBP, NAPG and PSMA3 were identified as target genes showing the highest number of integration with TFs. In this network genes such as HEPACAM2, calcyclin binding protein (CACYBP) and N-ethylmaleimide-sensitive factor attachment protein, gamma (NAPG) along TFs such as FOXC1, FOXL1, YY1 and USF2 were predicted as novel prognostic or diagnostic biomarkers and new therapeutic target in CAD. Transcription factor GATA2 was linked with pathogens of CAD [114]. Down regulated genes such as PAH, CYP3A4, CP, COX7C and TTTY10 were identified as target genes showing the highest number of integration with TFs. Phenylalanine hydroxylase (PAH) was associated with development of cardiovascular diseases [115], but this gene may be identified with advancement of CAD. In this network down regulated gene TTTY10 along transcription factor SRF (serum response factor) were predicted as novel prognostic or diagnostic biomarkers and new therapeutic target in CAD.

In conclusion, the present study identified 13 hub genes (up regulated) and 10 hub genes (down regulated) that may be associated in the progression of CAD. Among them, 12 hub genes (up regulated) and 7 hub genes (down regulated) are closely related to the prognosis of CAD. Also identified 10 miRNA and 10 TFs may be associated in the progression of CAD. These hub genes may be regarded as diagnostic and prognostic biomarkers, and could become potential therapeutic target for future CAD therapeutic strategies. However, bioinformatics only plays a predictive role; the function of these hub genes in CAD needs further study to elucidate the biological characteristics.

## Data Availability

The datasets supporting the conclusions of this article are available in the GEO (Gene Expression Omnibus) (https://www.ncbi.nlm.nih.gov/geo/) repository. [(GSE23766) (https://www.ncbi.nlm.nih.gov/geo/query/acc.cgi?acc=GSE23766)].

## Supplementary Materials

The following are available online at https://www.mdpi.com/2218-273X/10/1/35/s1.
