# Supplementary material for: Analysis of Differentially Expressed Genes in Coronary Artery Disease by Integrated Microarray Analysis
Source: Biomolecules. 2019 Dec 25;10(1):35. doi: 10.3390/biom10010035 (PMC7022900; doi:10.3390/biom10010035)
Supplement: Supplementary file 1 [file biomolecules-10-00035-s001.pdf]

Table 1 The statistical metrics for key differentially expressed genes (DEGs)

Agiliant Id	Gene Symbol	logFC	pValue	FDR	tvalue	Regulation	Gene Name
A_24_P237896	LOC649056	0.509843	9.18E-14	4.54E-11	12.07302	Up	PREDICTED: Homo sapiens similar to Filamin-C (Gamma-filamin) (Filamin-2) (Protein FLNc) (Actin-binding-like protein) (ABP-L) (ABP-280-like protein) (LOC649056), mRNA [XR_018580]
A_23_P18325	PDCD10	0.243111	2.8E-12	9.24E-10	10.62808	Up	Homo sapiens programmed cell death 10 (PDCD10), transcript variant 1, mRNA [NM_007217]
A_23_P141335	ARHGAP27	0.492709	3.97E-12	1.22E-09	10.48571	Up	Homo sapiens Rho GTPase activating protein 27 (ARHGAP27), mRNA [NM_199282]
A_23_P53110	TUB	0.528219	1.77E-11	4.56E-09	9.891033	Up	Homo sapiens tubby homolog (mouse) (TUB), transcript variant 1, mRNA [NM_003320]
A_23_P42168	MDFI	0.314474	1.81E-10	3.74E-08	8.998697	Up	Homo sapiens MyoD family inhibitor (MDFI), mRNA [NM_005586]
A_32_P56890	LOC644701	0.444703	3.6E-10	7.09E-08	8.743973	Up	PREDICTED: Homo sapiens hypothetical LOC644701 (LOC644701), mRNA [XM_932316]
A_32_P167111	A_32_P167111	0.873588	7.41E-10	1.4E-07	8.47781	Up	Unknown
A_24_P221424	ZNF784	0.686781	9.18E-10	1.68E-07	8.399687	Up	Homo sapiens zinc finger protein 784 (ZNF784), mRNA [NM_203374]
A_23_P74895	LIN28	0.218876	1.27E-09	2.24E-07	8.282224	Up	Homo sapiens lin-28 homolog (C. elegans) (LIN28), mRNA [NM_024674]
A_23_P12140	RPL5	0.247598	1.81E-09	3.11E-07	8.154317	Up	Homo sapiens ribosomal protein L5 (RPL5), mRNA [NM_000969]
A_24_P852082	LOC92482	0.212904	2.33E-09	3.87E-07	8.062867	Up	Homo sapiens cDNA FLJ43841 fis, clone TESTI4006137. [AK125829]
A_23_P19210	BXDC1	0.229614	2.43E-09	4.02E-07	8.04778	Up	Homo sapiens brix domain containing 1 (BXDC1), mRNA [NM_032194]
A_24_P417189	DUSP9	0.238423	2.47E-09	4.06E-07	8.042031	Up	Homo sapiens dual specificity phosphatase 9 (DUSP9), mRNA [NM_001395]
A_24_P655735	A_24_P655735	0.465887	4.93E-09	7.57E-07	7.794954	Up	Unknown
A_23_P128930	PSMC6	0.211435	5.26E-09	8.02E-07	7.771854	Up	Homo sapiens proteasome (prosome, macropain) 26S subunit, ATPase, 6 (PSMC6), mRNA [NM_002806]
A_23_P322076	ZNF697	0.458852	6.27E-09	9.38E-07	7.709988	Up	Homo sapiens zinc finger protein 697 (ZNF697), mRNA [NM_001080470]
A_32_P49423	NPM1	0.197369	8.59E-09	1.26E-06	7.598635	Up	Homo sapiens nucleophosmin (nucleolar phosphoprotein B23, numatrin) (NPM1), transcript variant 1, mRNA [NM_002520]
A_23_P154411	PPIG	0.24475	1.02E-08	1.46E-06	7.53862	Up	Homo sapiens peptidylprolyl isomerase G (cyclophilin G) (PPIG), mRNA [NM_004792]
A_23_P11739	MIXL1	0.454042	1.24E-08	1.75E-06	7.46905	Up	Homo sapiens Mix1 homeobox-like 1 (Xenopus laevis) (MIXL1), mRNA [NM_031944]
A_23_P102113	WNT10A	0.548292	1.45E-08	2.02E-06	7.414681	Up	Homo sapiens wingless-type MMTV integration site family, member 10A (WNT10A), mRNA [NM_025216]
A_24_P545807	A_24_P545807	0.2424	1.73E-08	2.36E-06	7.353909	Up	Unknown
A_23_P77818	ATP5H	0.222976	1.78E-08	2.42E-06	7.343264	Up	Homo sapiens ATP synthase, H+ transporting, mitochondrial F0 complex, subunit d (ATP5H), nuclear gene encoding mitochondrial protein, transcript variant 1, mRNA

							[NM_006356]
A_32_P219126	AL566332	0.38112	2.2E-08	2.97E-06	7.268967	Up	AL566332 AL566332 Homo sapiens FETAL BRAIN Homo sapiens cDNA clone CS0DF015YB18 3-PRIME, mRNA sequence [AL566332]
A_24_P158903	IRAK4	0.194633	5.37E-08	6.63E-06	6.960491	Up	Homo sapiens interleukin-1 receptor-associated kinase 4 (IRAK4), mRNA [NM_016123]
A_23_P8281	IFNGR1	0.217078	7.04E-08	8.49E-06	6.86702	Up	Homo sapiens interferon gamma receptor 1 (IFNGR1), mRNA [NM_000416]
A_23_P114445	MAGEE1	0.296376	1.07E-07	1.26E-05	6.724063	Up	Homo sapiens melanoma antigen family E, 1 (MAGEE1), mRNA [NM_020932]
A_24_P103944	ATM	0.290111	1.2E-07	1.4E-05	6.68513	Up	Homo sapiens ataxia telangiectasia mutated (includes complementation groups A, C and D), mRNA (cDNA clone IMAGE:4290281), complete cds. [BC022307]
A_23_P69908	GLRX	0.232477	1.32E-07	1.53E-05	6.651658	Up	Homo sapiens glutaredoxin (thioltransferase) (GLRX), mRNA [NM_002064]
A_23_P142407	ZNF101	0.211492	1.42E-07	1.63E-05	6.627825	Up	Homo sapiens zinc finger protein 101 (ZNF101), mRNA [NM_033204]
A_32_P176018	ACTL8	0.320954	1.52E-07	1.73E-05	6.60323	Up	Homo sapiens actin-like 8 (ACTL8), mRNA [NM_030812]
A_24_P401870	C9orf139	0.283575	1.8E-07	2.02E-05	6.546414	Up	Homo sapiens chromosome 9 open reading frame 139 (C9orf139), mRNA [NM_207511]
A_24_P374461	ZNF804B	0.302123	1.84E-07	2.06E-05	6.538052	Up	Homo sapiens zinc finger protein 804B (ZNF804B), mRNA [NM_181646]
A_23_P407090	NFXL1	0.175415	2.23E-07	2.45E-05	6.473312	Up	Homo sapiens nuclear transcription factor, X-box binding-like 1 (NFXL1), mRNA [NM_152995]
A_23_P92650	SLC25A2	0.260309	2.24E-07	2.46E-05	6.471641	Up	Homo sapiens solute carrier family 25 (mitochondrial carrier; ornithine transporter) member 2 (SLC25A2), nuclear gene encoding mitochondrial protein, mRNA [NM_031947]
A_23_P200936	MTR	0.24618	2.42E-07	2.64E-05	6.445762	Up	Homo sapiens 5-methyltetrahydrofolate-homocysteine methyltransferase (MTR), mRNA [NM_000254]
A_24_P940457	AA458851	0.249255	3.37E-07	3.58E-05	6.333288	Up	AA458851 aa26c08.s1 NCI_CGAP_GCB1 Homo sapiens cDNA clone IMAGE:814382 3' similar to contains Alu repetitive element;, mRNA sequence [AA458851]
A_32_P138933	A_32_P138933	0.303245	3.49E-07	3.68E-05	6.321376	Up	Unknown
A_24_P764595	PTP4A2P1	0.199621	3.64E-07	3.82E-05	6.306887	Up	protein tyrosine phosphatase type IVA, member 2 pseudogene 1 [
A_23_P154447	NOP58	0.254899	4.62E-07	4.73E-05	6.226598	Up	Homo sapiens nucleolar protein NOP58 NOP58, mRNA [NM_015934]
A_24_P7228	ENST00000320662	0.199039	5.37E-07	5.41E-05	6.175832	Up	Homo sapiens chromosome 8 open reading frame 56, mRNA (cDNA clone IMAGE:4820412). [BC029562]
A_24_P194670	RP11-218C14.6	0.285381	5.82E-07	5.81E-05	6.148082	Up	Homo sapiens cystatin pseudogene (LOC164380) on chromosome 20 [NR_001279]
A_24_P253780	DBF4B	0.235473	6.16E-07	6.1E-05	6.129076	Up	Homo sapiens DBF4 homolog B (S. cerevisiae) (DBF4B), transcript variant 1, mRNA [NM_145663]
A_24_P24770	SLC9A10	0.206055	6.69E-07	6.55E-05	6.101405	Up	Homo sapiens solute carrier family 9, member 10 (SLC9A10), mRNA [NM_183061]
A_23_P82068	NOL7	0.18435	7.36E-07	7.11E-05	6.069302	Up	Homo sapiens nucleolar protein 7, 27kDa (NOL7), mRNA [NM_016167]
A_32_P113436	RP11-78J21.1	0.178095	7.66E-07	7.39E-05	6.055602	Up	Homo sapiens heterogeneous nuclear ribonucleoprotein A1-like

							(LOC144983), transcript variant 1, mRNA [NM_001011724]
A_32_P125103	AA902595	0.204354	7.69E-07	7.4E-05	6.054299	Up	AA902595 oj60c08.s1 NCI_CGAP_Kid3 Homo sapiens cDNA clone IMAGE:1502702 3', mRNA sequence [AA902595]
A_24_P925518	A_24_P925518	0.310077	8.29E-07	7.94E-05	6.029195	Up	Unknown
A_24_P152753	LOC285260	0.174159	8.38E-07	7.98E-05	6.025319	Up	PREDICTED: Homo sapiens similar to ribosomal protein L31 (LOC285260), mRNA [XR_019376]
A_23_P91699	EIF3S7	0.16952	8.39E-07	7.98E-05	6.024922	Up	Homo sapiens eukaryotic translation initiation factor 3, subunit 7 zeta, 66/67kDa (EIF3S7), mRNA [NM_003753]
A_24_P170186	LOC652423	0.250976	8.47E-07	8.03E-05	6.021607	Up	PREDICTED: Homo sapiens similar to nucleophosmin 1 isoform 1 (LOC652423), mRNA [XR_019315]
A_23_P502590	KIR2DS4	0.23622	9.28E-07	8.73E-05	5.990844	Up	Homo sapiens killer cell immunoglobulin-like receptor, two domains, short cytoplasmic tail, 4 (KIR2DS4), mRNA [NM_012314]
A_23_P144521	ADAMTS3	0.306151	1.05E-06	9.7E-05	5.949165	Up	Homo sapiens ADAM metallopeptidase with thrombospondin type 1 motif, 3 (ADAMTS3), mRNA [NM_014243]
A_32_P114574	CACYBP	0.178042	1.19E-06	0.000107	5.907809	Up	Homo sapiens calcyclin binding protein (CACYBP), transcript variant 1, mRNA [NM_014412]
A_23_P255714	IFT74	0.199324	1.46E-06	0.000128	5.839114	Up	Homo sapiens intraflagellar transport 74 homolog (Chlamydomonas) (IFT74), mRNA [NM_025103]
A_32_P172698	A_32_P172698	0.263194	1.49E-06	0.000131	5.832162	Up	Unknown
A_24_P67552	LOC731058	0.249313	1.5E-06	0.000131	5.828882	Up	PREDICTED: Homo sapiens hypothetical protein LOC731058 (LOC731058), mRNA [XR_015778]
A_24_P358337	A_24_P358337	0.225366	1.69E-06	0.000147	5.789699	Up	Unknown
A_23_P43566	NDUFA8	0.171967	2.15E-06	0.000181	5.707776	Up	Homo sapiens NADH dehydrogenase (ubiquinone) 1 alpha subcomplex, 8, 19kDa (NDUFA8), nuclear gene encoding mitochondrial protein, mRNA [NM_014222]
A_32_P166152	LOC732417	0.169514	2.17E-06	0.000181	5.705787	Up	PREDICTED: Homo sapiens hypothetical protein LOC732417 (LOC732417), mRNA [XM_001133403]
A_23_P34007	PPP1R2P9	0.308542	2.42E-06	0.000198	5.668597	Up	Homo sapiens protein phosphatase 1, regulatory (inhibitor) subunit 2 pseudogene 9 (PPP1R2P9) on chromosome X [NR_002191]
A_24_P50228	C11orf87	0.280534	2.52E-06	0.000205	5.654983	Up	chromosome 11 open reading frame 87
A_32_P40235	DPP10-AS3	0.278894	2.82E-06	0.000227	5.617099	Up	DPP10 antisense RNA 3
A_23_P213153	HNRPDL	0.215705	2.88E-06	0.000231	5.610186	Up	Homo sapiens heterogeneous nuclear ribonucleoprotein D-like (HNRPDL), transcript variant 2, mRNA [NM_031372]
A_23_P60565	ZNF354A	0.199548	3.15E-06	0.000251	5.57972	Up	Homo sapiens zinc finger protein 354A (ZNF354A), mRNA [NM_005649]
A_24_P676878	F03017	0.312392	3.29E-06	0.000259	5.565206	Up	HSC1JF102 normalized infant brain cDNA Homo sapiens cDNA clone c-1jf10 3', mRNA sequence [F03017]
A_23_P132863	LSM3	0.191107	3.36E-06	0.000262	5.558741	Up	LSM3 homolog, U6 small nuclear RNA and mRNA degradation associated
A_23_P158148	CCT6A	0.244163	3.44E-06	0.000267	5.550648	Up	Homo sapiens chaperonin containing TCP1, subunit 6A (zeta 1) (CCT6A), transcript variant 1, mRNA [NM_001762]
A_24_P248031	BC017945	0.19698	4.2E-06	0.000319	5.483589	Up	Homo sapiens cDNA clone IMAGE:4293510, partial cds. [BC017945]
A_24_P801264	HMGB1	0.224732	4.5E-06	0.000337	5.460453	Up	Homo sapiens high-mobility group box 1 (HMGB1), mRNA [NM_002128]

A_24_P536218	THC2645872	0.276857	4.6E-06	0.000342	5.45309	Up	ALU1_HUMAN (P39188) Alu subfamily J sequence contamination warning entry, partial (13%) [THC2645872]
A_24_P794024	THC2688335	0.210397	5.15E-06	0.000377	5.414948	Up	Q4SZ31_TETNG (Q4SZ31) Chromosome undetermined SCAF11859, whole genome shotgun sequence. (Fragment), partial (3%) [THC2688335]
A_24_P829209	HOXA-AS3	0.21821	5.22E-06	0.000381	5.41053	Up	HOXA cluster antisense RNA 3
A_23_P105276	ZNF84	0.192191	5.67E-06	0.000408	5.382928	Up	Homo sapiens zinc finger protein 84 (ZNF84), mRNA [NM_003428]
A_24_P672240	FRMPD4	0.292206	5.7E-06	0.000408	5.381271	Up	PDZ domain containing 10 [Source:RefSeq_peptide;Acc:NP_055543] [ENST00000380682]
A_24_P920629	BE671947	0.225413	5.71E-06	0.000408	5.380651	Up	BE671947 7a46g09.x1 NCI_CGAP_GC6 Homo sapiens cDNA clone IMAGE:3221824 3', mRNA sequence [BE671947]
A_24_P817186	A_24_P817186	0.20335	6.14E-06	0.000434	5.356061	Up	Unknown
A_32_P757733	CEP70	0.182894	6.53E-06	0.000458	5.335323	Up	Homo sapiens centrosomal protein 70kDa (CEP70), mRNA [NM_024491]
A_24_P776036	A_24_P776036	0.232906	6.61E-06	0.000463	5.331199	Up	Unknown
A_23_P205216	UTP14A	0.170246	6.75E-06	0.000468	5.324223	Up	Homo sapiens UTP14, U3 small nucleolar ribonucleoprotein, homolog A (yeast) (UTP14A), mRNA [NM_006649]
A_24_P916746	THC2672343	0.249088	6.75E-06	0.000468	5.324201	Up	Unknown
A_24_P416014	C15orf15	0.200735	7.38E-06	0.000505	5.294233	Up	Homo sapiens chromosome 15 open reading frame 15 (C15orf15), mRNA [NM_016304]
A_24_P385024	BC031359	0.196402	8.11E-06	0.000544	5.262794	Up	Homo sapiens, clone IMAGE:4778855, mRNA, partial cds. [BC031359]
A_32_P48842	SMIM10	0.217992	8.43E-06	0.000561	5.24953	Up	small integral membrane protein 10
A_24_P37939	RPS14	0.237043	9.33E-06	0.000611	5.215674	Up	40S ribosomal protein S14. [Source:Uniprot/SWISSPROT;Acc:P62263] [ENST00000312037]
A_24_P928888	TMEM74	0.172424	9.37E-06	0.000612	5.214115	Up	transmembrane protein 74
A_24_P367421	LOC647968	0.182509	9.82E-06	0.000636	5.198208	Up	PREDICTED: Homo sapiens similar to M-phase phosphoprotein 10 (LOC647968), mRNA [XR_018268]
A_24_P301186	WDR89	0.187182	9.87E-06	0.000638	5.19674	Up	Homo sapiens WD repeat domain 89 (WDR89), transcript variant 1, mRNA [NM_001008726]
A_24_P902728	CAPRIN1	0.180177	9.99E-06	0.000645	5.192614	Up	Homo sapiens GPI-anchored membrane protein 1 (GPIAP1), transcript variant 2, mRNA [NM_203364]
A_24_P322229	RASL10B	0.267746	1.06E-05	0.000674	5.172192	Up	Homo sapiens RAS-like, family 10, member B (RASL10B), mRNA [NM_033315]
A_23_P354942	ZNF493	0.181066	1.07E-05	0.000676	5.170667	Up	Homo sapiens zinc finger protein 493 (ZNF493), transcript variant 1, mRNA [NM_175910]
A_24_P265407	AF289570	0.171552	1.09E-05	0.000688	5.163194	Up	Homo sapiens clone pp6750 unknown mRNA. [AF289570]
A_23_P13007	FKSG44	0.215709	1.13E-05	0.000708	5.152182	Up	Homo sapiens FKSG44 (FKSG44) mRNA, complete cds. [AF334946]
A_24_P647507	CR739597	0.341774	1.17E-05	0.000727	5.140479	Up	CR739597 CR739597 NCI_CGAP_Kid11 Homo sapiens cDNA clone IMAGp998E215695 ; IMAGE:2298620 5', mRNA sequence [CR739597]
A_23_P63816	NRBF2	0.177201	1.26E-05	0.000777	5.113497	Up	Homo sapiens nuclear receptor binding factor 2 (NRBF2), mRNA [NM_030759]

A_24_P161463	C20orf199	0.216436	1.35E-05	0.000823	5.091158	Up	Homo sapiens unknown protein 2 mRNA, complete cds. [AY513723]
A_23_P431890	DNAJC19	0.181045	1.38E-05	0.00084	5.082653	Up	Homo sapiens DnaJ (Hsp40) homolog, subfamily C, member 19 (DNAJC19), mRNA [NM_145261]
A_23_P322086	C12orf39	0.250927	1.42E-05	0.000857	5.073324	Up	Homo sapiens chromosome 12 open reading frame 39 (C12orf39), mRNA [NM_030572]
A_24_P196528	CRB1	0.229787	1.44E-05	0.000868	5.068602	Up	Homo sapiens crumbs homolog 1 (Drosophila) (CRB1), mRNA [NM_201253]
A_24_P374427	ZDHHC21	0.173802	1.5E-05	0.000897	5.055737	Up	Homo sapiens zinc finger, DHHC-type containing 21 (ZDHHC21), mRNA [NM_178566]
A_23_P252125	TRIP6	0.213019	1.51E-05	0.000902	5.052466	Up	thyroid hormone receptor interactor 6
A_32_P106466	THC2545456	0.232208	1.52E-05	0.000903	5.051282	Up	Unknown
A_23_P124912	NPEPPS	0.215224	1.58E-05	0.000936	5.037544	Up	Homo sapiens aminopeptidase puromycin sensitive (NPEPPS), mRNA [NM_006310]
A_23_P408271	HSD17B11	0.20349	1.67E-05	0.000981	5.020255	Up	Homo sapiens hydroxysteroid (17-beta) dehydrogenase 11 (HSD17B11), mRNA [NM_016245]
A_24_P484196	H92965	0.192622	1.7E-05	0.000995	5.014341	Up	H92965 yv07a03.s1 Soares fetal liver spleen 1NFLS Homo sapiens cDNA clone IMAGE:241996 3' similar to contains Alu repetitive element;, mRNA sequence [H92965]
A_24_P418517	LOC390411	0.205095	1.76E-05	0.00102	5.002597	Up	PREDICTED: Homo sapiens similar to nucleophosmin 1 isoform 1 (LOC390411), mRNA [XR_018240]
A_32_P218812	AK090670	0.206814	1.83E-05	0.001052	4.98948	Up	Homo sapiens cDNA FLJ33351 fis, clone BRACE2005063. [AK090670]
A_32_P118397	HEPACAM2	0.249188	1.91E-05	0.001089	4.974765	Up	HEPACAM family member 2
A_24_P201702	CLEC2B	0.18675	2.09E-05	0.001178	4.944365	Up	Homo sapiens C-type lectin domain family 2, member B (CLEC2B), mRNA [NM_005127]
A_23_P24097	C10orf128	0.171385	2.09E-05	0.001178	4.944086	Up	Homo sapiens cDNA clone IMAGE:5168526. [BC031641]
A_23_P88776	PRSS54	0.351758	2.16E-05	0.001214	4.932344	Up	serine protease 54
A_32_P67978	BC008341	0.245436	2.22E-05	0.001241	4.924243	Up	Homo sapiens cDNA clone IMAGE:3509098, **** WARNING: chimeric clone ****. [BC008341]
A_32_P178635	BC033590	0.255687	2.46E-05	0.001347	4.889674	Up	Homo sapiens, clone IMAGE:4344826, mRNA. [BC033590]
A_24_P171268	RASSF5	0.177186	2.46E-05	0.001347	4.888969	Up	Homo sapiens Ras association (RalGDS/AF-6) domain family 5 (RASSF5), transcript variant 3, mRNA [NM_182665]
A_24_P148653	LSG1	0.16996	2.52E-05	0.001374	4.880521	Up	Homo sapiens large subunit GTPase 1 homolog (S. cerevisiae) (LSG1), mRNA [NM_018385]
A_32_P787109	DTHD1	0.211041	2.57E-05	0.001392	4.874832	Up	death domain containing 1
A_23_P35906	CASP4	0.189963	2.71E-05	0.001449	4.856633	Up	Homo sapiens caspase 4, apoptosis-related cysteine peptidase (CASP4), transcript variant gamma, mRNA [NM_033306]
A_23_P431591	C5orf39	0.237254	2.76E-05	0.001467	4.850075	Up	Homo sapiens similar to annexin II receptor (AXIIR), mRNA [NM_001014279]
A_24_P204639	ZNF716	0.170801	2.83E-05	0.001497	4.841398	Up	zinc finger protein 716
A_32_P182609	BE008305	0.23262	2.86E-05	0.00151	4.837997	Up	BE008305 CM0-BN0154-080400-325-a07 BN0154 Homo sapiens cDNA, mRNA sequence [BE008305]
A_24_P914918	BM677097	0.222147	2.88E-05	0.001516	4.835976	Up	BM677097 UI-E-EO1-aic-e-03-0-UI.s1 UI-E-EO1 Homo sapiens cDNA clone UI-E-EO1-aic-e-03-0-UI 3', mRNA

							sequence [BM677097]
A_24_P920483	A_24_P920483	0.192639	3.1E-05	0.001611	4.811323	Up	Unknown
A_32_P200608	LOC151300	0.179885	3.21E-05	0.001664	4.798622	Up	Homo sapiens cDNA clone IMAGE:4839086. [BC049373]
A_24_P931306	THC2487053	0.21588	3.22E-05	0.001666	4.797788	Up	Unknown
A_24_P394075	NT5C1B	0.337433	3.24E-05	0.001672	4.79624	Up	Homo sapiens 5'-nucleotidase, cytosolic IB (NT5C1B), transcript variant 1, mRNA [NM_001002006]
A_32_P57775	CA313037	0.244585	3.37E-05	0.001727	4.782586	Up	CA313037 UI-CF-FN0-aex-g-14-0-UI.s1 UI-CF-FN0 Homo sapiens cDNA clone UI-CF-FN0-aex-g-14-0-UI 3', mRNA sequence [CA313037]
A_23_P140301	PSMA3	0.186392	3.45E-05	0.001759	4.774297	Up	Homo sapiens proteasome (prosome, macropain) subunit, alpha type, 3 (PSMA3), transcript variant 1, mRNA [NM_002788]
A_32_P524014	UTRN	0.200837	3.58E-05	0.001819	4.762142	Up	Homo sapiens utrophin (UTRN), mRNA [NM_007124]
A_24_P50458	TERF1	0.1889	3.67E-05	0.001858	4.754117	Up	Homo sapiens telomeric repeat binding factor (NIMA-interacting) 1 (TERF1), transcript variant 1, mRNA [NM_017489]
A_24_P177531	PCDHGB8P	0.18168	3.8E-05	0.001913	4.742157	Up	Homo sapiens protocadherin gamma subfamily B, 8 pseudogene (PCDHGB8P) on chromosome 5 [NR_001297]
A_24_P175187	SAMD9	0.220148	3.83E-05	0.001921	4.739544	Up	Homo sapiens sterile alpha motif domain containing 9 (SAMD9), mRNA [NM_017654]
A_24_P557355	AF070564	0.181476	3.89E-05	0.001941	4.733878	Up	Homo sapiens clone 24571 mRNA sequence. [AF070564]
A_32_P425876	AK095904	0.195024	4.01E-05	0.001994	4.723573	Up	Homo sapiens cDNA FLJ38585 fis, clone HCHON2009191. [AK095904]
A_23_P162378	CCDC41	0.186036	4.13E-05	0.002032	4.713669	Up	Homo sapiens coiled-coil domain containing 41 (CCDC41), transcript variant 1, mRNA [NM_016122]
A_32_P100771	BX118374	0.202443	4.13E-05	0.002032	4.71347	Up	BX118374 NCI_CGAP_Pr28 Homo sapiens cDNA clone IMAGp998J145570, mRNA sequence [BX118374]
A_24_P653603	NDUFAF8	0.238431	4.21E-05	0.00206	4.706847	Up	NADH:ubiquinone oxidoreductase complex assembly factor 8
A_32_P110156	CCT6AP1	0.210885	4.21E-05	0.00206	4.706802	Up	Homo sapiens chaperonin containing TCP1, subunit 6A (zeta 1) pseudogene 1 (CCT6AP1) on chromosome 7 [NR_003110]
A_23_P204689	CLEC2D	0.295509	4.56E-05	0.002205	4.68006	Up	Homo sapiens C-type lectin domain family 2, member D (CLEC2D), transcript variant 2, mRNA [NM_001004419]
A_32_P109036	ZNF493	0.205008	4.57E-05	0.002208	4.679169	Up	Homo sapiens zinc finger protein 493 (ZNF493), transcript variant 1, mRNA [NM_175910]
A_23_P257063	PPP1R42	0.316755	4.59E-05	0.002209	4.67824	Up	protein phosphatase 1 regulatory subunit 42
A_32_P515920	TMEM238L	0.289694	4.74E-05	0.00227	4.667059	Up	transmembrane protein 238 like
A_24_P647163	BG777521	0.180443	4.83E-05	0.002306	4.660496	Up	602664870F1 NIH_MGC_60 Homo sapiens cDNA clone IMAGE:4804769 5', mRNA sequence [BG777521]
A_23_P162782	ARGLU1	0.199096	4.86E-05	0.002315	4.658711	Up	arginine and glutamate rich 1
A_23_P303417	ERICH1	0.196704	4.96E-05	0.002356	4.651594	Up	glutamate rich 1
A_24_P307384	LOC345041	0.201808	5.18E-05	0.002431	4.637036	Up	PREDICTED: Homo sapiens similar to 60 kDa heat shock protein, mitochondrial precursor (Hsp60) (60 kDa chaperonin) (CPN60) (Heat shock protein 60) (HSP-60) (Mitochondrial matrix protein P1) (P60 lymphocyte protein) (HuCHA60)

							(LOC345041), mRNA [XR_018747]
A_24_P19268	ZNF708	0.172365	5.29E-05	0.002473	4.629365	Up	Homo sapiens zinc finger protein 708 (ZNF708), mRNA [NM_021269]
A_32_P111293	LINC02212	0.241982	5.31E-05	0.002477	4.628138	Up	long intergenic non-protein coding RNA 2212
A_24_P758010	AJ318805	0.231475	5.43E-05	0.002523	4.620655	Up	AJ318805 Homo sapiens adipose tissue Homo sapiens cDNA clone 2040, mRNA sequence [AJ318805]
A_23_P385246	KCTD6	0.216564	5.49E-05	0.002539	4.617276	Up	Homo sapiens potassium channel tetramerisation domain containing 6 (KCTD6), mRNA [NM_153331]
A_24_P796428	A_24_P796428	0.171887	5.54E-05	0.002554	4.614127	Up	Unknown
A_32_P134301	A_32_P134301	0.188024	5.55E-05	0.002555	4.613567	Up	Unknown
A_24_P415260	DDX21	0.26825	5.72E-05	0.00261	4.602937	Up	Nucleolar RNA helicase 2 (EC 3.6.1.-) (Nucleolar RNA helicase II) (Nucleolar RNA helicase Gu) (RH II/Gu) (Gu-alpha) (DEAD box protein 21). [Source:Uniprot/SWISSPROT;Acc:Q9NR30] [ENST00000354185]
A_24_P926554	AK056365	0.233604	5.73E-05	0.002611	4.602472	Up	Homo sapiens cDNA FLJ31803 fis, clone NT2RI2009101. [AK056365]
A_23_P94911	ZNF283	0.182726	5.74E-05	0.002612	4.601889	Up	Homo sapiens cDNA FLJ40856 fis, clone TRACH2016498, moderately similar to ZINC FINGER PROTEIN 184. [AK098175]
A_24_P59053	TRBV3-1	0.271526	5.83E-05	0.002647	4.596603	Up	T-cell receptor beta V gene segment 3-1 [Source:IMGT/GENE_DB;Acc:TRBV3-1] [ENST00000390350]
A_32_P134580	HMGB1	0.250427	5.95E-05	0.002691	4.589836	Up	Homo sapiens high-mobility group box 1 (HMGB1), mRNA [NM_002128]
A_24_P863124	OR2A1-AS1	0.197548	5.99E-05	0.002707	4.587539	Up	Q44ZN8_9BURK (Q44ZN8) Acyl-CoA dehydrogenase, C-terminal:Acyl-CoA dehydrogenase, central region, partial (3%) [THC2776730]
A_23_P166023	PFDN4	0.197393	6.15E-05	0.002756	4.578148	Up	Homo sapiens prefoldin subunit 4 (PFDN4), mRNA [NM_002623]
A_24_P190894	ARF1	0.220545	6.17E-05	0.002756	4.57723	Up	Homo sapiens ADP-ribosylation factor 1 (ARF1), transcript variant 4, mRNA [NM_001658]
A_24_P6125	KCNJ4	0.179271	6.26E-05	0.002787	4.572355	Up	Homo sapiens potassium inwardly-rectifying channel, subfamily J, member 4 (KCNJ4), transcript variant 1, mRNA [NM_152868]
A_23_P83976	CCDC46	0.216406	6.58E-05	0.002906	4.555144	Up	Homo sapiens coiled-coil domain containing 46 (CCDC46), transcript variant 1, mRNA [NM_145036]
A_23_P168771	KIAA1505	0.230259	6.63E-05	0.00292	4.552753	Up	Homo sapiens KIAA1505 protein (KIAA1505), mRNA [NM_020879]
A_24_P33508	MARCH11	0.278157	6.71E-05	0.002937	4.548976	Up	membrane associated ring-CH-type finger 11
A_24_P766812	THC2693652	0.195709	6.79E-05	0.002959	4.544646	Up	ALU2_HUMAN (P39189) Alu subfamily SB sequence contamination warning entry, partial (7%) [THC2693652]
A_24_P916585	ENTPD6	0.182984	6.84E-05	0.002974	4.542123	Up	Homo sapiens IL-6SAG (IL-6SAG) mRNA, partial cds. [AF058296]
A_23_P320887	CDC42SE1	0.551171	7.06E-05	0.003051	4.531273	Up	Homo sapiens CDC42 small effector 1 (CDC42SE1), transcript variant 2, mRNA [NM_020239]
A_32_P117127	ODZ3	0.199	7.24E-05	0.003097	4.523007	Up	Homo sapiens odz, odd Oz/ten-m homolog 3 (Drosophila) (ODZ3), mRNA [NM_001080477]

A_24_P363005	UBE2D3	0.220911	7.28E-05	0.003106	4.52112	Up	Homo sapiens ubiquitin-conjugating enzyme E2D 3 (UBC4/5 homolog, yeast) (UBE2D3), transcript variant 2, mRNA [NM_181886]
A_24_P71904	HPGD	0.275684	7.49E-05	0.003168	4.511006	Up	Homo sapiens hydroxyprostaglandin dehydrogenase 15-(NAD) (HPGD), mRNA [NM_000860]
A_23_P139143	STX3	0.202864	7.98E-05	0.00332	4.489394	Up	Homo sapiens syntaxin 3 (STX3), mRNA [NM_004177]
A_32_P145769	BC043547	0.275453	8.21E-05	0.003396	4.47995	Up	Homo sapiens, clone IMAGE:5171873, mRNA. [BC043547]
A_24_P150361	HSP90B1	0.206502	8.22E-05	0.003398	4.479365	Up	Homo sapiens heat shock protein 90kDa beta (Grp94), member 1 (HSP90B1), mRNA [NM_003299]
A_24_P921390	A_24_P921390	0.195455	8.64E-05	0.003555	4.462606	Up	Unknown
A_32_P47543	PGGT1B	0.205142	8.82E-05	0.003614	4.45525	Up	protein geranylgeranyltransferase type I subunit beta
A_24_P188941	NPM1	0.174517	9.23E-05	0.003728	4.439848	Up	Homo sapiens nucleophosmin (nucleolar phosphoprotein B23, numatrin) (NPM1), transcript variant 1, mRNA [NM_002520]
A_32_P473302	VLDLR-AS1	0.216625	9.42E-05	0.003788	4.432717	Up	VLDLR antisense RNA 1
A_24_P921719	A_24_P921719	0.207547	0.0001	0.003986	4.412265	Up	Unknown
A_24_P108779	KCTD17	0.190094	0.000101	0.004027	4.408275	Up	Homo sapiens potassium channel tetramerisation domain containing 17 (KCTD17), mRNA [NM_024681]
A_32_P114215	COMMD6	0.191236	0.000103	0.004066	4.403474	Up	Homo sapiens COMM domain containing 6 (COMMD6), transcript variant 2, mRNA [NM_203495]
A_24_P617046	AW182715	0.185693	0.000103	0.004071	4.402726	Up	AW182715 xp96g01.x1 Soares_NHCeC_cervical_tumor Homo sapiens cDNA clone IMAGE:2748240 3', mRNA sequence [AW182715]
A_23_P13364	NUCB2	0.204288	0.00011	0.004256	4.380856	Up	Homo sapiens nucleobindin 2 (NUCB2), mRNA [NM_005013]
A_24_P13406	C12orf40	0.180588	0.000111	0.004301	4.376273	Up	Homo sapiens chromosome 12 open reading frame 40 (C12orf40), mRNA [NM_001031748]
A_24_P554331	AK025173	0.230044	0.000114	0.004381	4.367057	Up	Homo sapiens cDNA: FLJ21520 fis, clone COL05874. [AK025173]
A_24_P548795	CN358250	0.337356	0.000118	0.004488	4.356664	Up	CN358250 17000600106015 GRN_PRENEU Homo sapiens cDNA 5', mRNA sequence [CN358250]
A_24_P170593	CBWD3	0.179987	0.000118	0.004506	4.354522	Up	Homo sapiens clone 1659351 unknown mRNA. [AF293368]
A_32_P129660	GCC2	0.17659	0.000119	0.004519	4.353235	Up	Homo sapiens GRIP and coiled-coil domain containing 2 (GCC2), transcript variant 1, mRNA [NM_181453]
A_24_P376159	AK090720	0.256132	0.00012	0.004539	4.351037	Up	Homo sapiens cDNA FLJ33401 fis, clone BRACE2009886. [AK090720]
A_23_P87773	C12orf48	0.207137	0.000121	0.004591	4.345873	Up	Homo sapiens chromosome 12 open reading frame 48 (C12orf48), mRNA [NM_017915]
A_23_P381992	ITGAV	0.278831	0.000122	0.004594	4.345053	Up	Homo sapiens integrin, alpha V (vitronectin receptor, alpha polypeptide, antigen CD51) (ITGAV), mRNA [NM_002210]
A_24_P182929	KCNAB1	0.220724	0.000126	0.004674	4.334021	Up	Homo sapiens potassium voltage-gated channel, shaker-related subfamily, beta member 1 (KCNAB1), transcript variant 2, mRNA [NM_003471]
A_24_P33429	LOC442006	0.215266	0.000127	0.004689	4.331387	Up	PREDICTED: Homo sapiens similar to Nucleophosmin (NPM) (Nucleolar phosphoprotein B23) (Numatrin) (Nucleolar protein NO38) (LOC442006), mRNA [XR_018535]
A_23_P56304	ZNF430	0.177471	0.00013	0.004804	4.32181	Up	Homo sapiens zinc finger protein 430 (ZNF430), mRNA

							[NM_025189]
A_23_P118705	SDF2	0.209247	0.000133	0.004884	4.313313	Up	Homo sapiens stromal cell-derived factor 2 (SDF2), mRNA [NM_006923]
A_23_P113258	RASGRP1	0.22291	0.000138	0.005031	4.301588	Up	Homo sapiens RAS guanyl releasing protein 1 (calcium and DAG-regulated) (RASGRP1), mRNA [NM_005739]
A_24_P119413	HSF5	0.181351	0.000139	0.005041	4.300297	Up	Homo sapiens heat shock transcription factor family member 5 (HSF5), mRNA [NM_001080439]
A_24_P75888	A_24_P75888	0.171597	0.000145	0.005219	4.285324	Up	Unknown
A_32_P218030	BU633383	0.191545	0.000146	0.005259	4.282381	Up	BU633383 UI-H-FL1-bgu-h-03-0-UI.s1 NCI_CGAP_FL1 Homo sapiens cDNA clone UI-H-FL1-bgu-h-03-0-UI 3', mRNA sequence [BU633383]
A_24_P934497	AC090371.2	0.198093	0.000154	0.005491	4.264182	Up	novel transcript, antisense to C18orf34
A_23_P97860	LIPA	0.195366	0.000157	0.005548	4.257338	Up	Homo sapiens lipase A, lysosomal acid, cholesterol esterase (Wolman disease) (LIPA), mRNA [NM_000235]
A_32_P110604	THC2641678	0.174724	0.000165	0.005738	4.24091	Up	Unknown
A_32_P146382	BG181407	0.204121	0.000165	0.005738	4.24066	Up	RST251 Athersys RAGE Library Homo sapiens cDNA, mRNA sequence [BG181407]
A_23_P106322	CPEB1	0.196088	0.000169	0.00587	4.230824	Up	Homo sapiens cytoplasmic polyadenylation element binding protein 1 (CPEB1), transcript variant 1, mRNA [NM_030594]
A_24_P57889	KIAA0913	0.191913	0.000171	0.005925	4.226779	Up	Homo sapiens mRNA for KIAA0913 protein, partial cds. [AB020720]
A_24_P273284	A_24_P273284	0.21886	0.000173	0.005969	4.223223	Up	Unknown
A_23_P13548	CHRDL2	0.305895	0.000179	0.006125	4.212892	Up	Homo sapiens chordin-like 2 (CHRDL2), mRNA [NM_015424]
A_23_P103442	C1orf149	0.187263	0.000185	0.006299	4.201434	Up	Homo sapiens chromosome 1 open reading frame 149 (C1orf149), mRNA [NM_022756]
A_23_P207881	A_23_P207881	0.179704	0.000187	0.00635	4.196332	Up	Unknown
A_23_P366468	RPF2P1	0.239444	0.000188	0.006368	4.194146	Up	ribosome production factor 2 homolog pseudogene 1
A_23_P161338	PPA1	0.178036	0.000189	0.006372	4.193465	Up	Homo sapiens pyrophosphatase (inorganic) 1 (PPA1), mRNA [NM_021129]
A_23_P44857	TRAV26-1	0.189071	0.000189	0.006381	4.192411	Up	T cell receptor alpha variable 26-1
A_23_P500400	ABCA6	0.242372	0.00019	0.006391	4.191581	Up	Homo sapiens ATP-binding cassette, sub-family A (ABC1), member 6 (ABCA6), mRNA [NM_080284]
A_32_P150012	CHD7	0.193588	0.000204	0.006742	4.167386	Up	Homo sapiens chromodomain helicase DNA binding protein 7 (CHD7), mRNA [NM_017780]
A_32_P163805	C11orf64	0.20523	0.000206	0.006811	4.162715	Up	Homo sapiens cDNA FLJ25394 fis, clone TST02552. [AK058123]
A_23_P118660	DDX52	0.178225	0.00022	0.007155	4.140619	Up	Homo sapiens DEAD (Asp-Glu-Ala-Asp) box polypeptide 52 (DDX52), transcript variant 2, mRNA [NM_152300]
A_23_P92120	FLJ44379	0.175845	0.000228	0.007392	4.127675	Up	Homo sapiens similar to S-100 protein, alpha chain (FLJ44379), mRNA [NM_001080537]
A_23_P86195	SLC44A3	0.23878	0.000235	0.007581	4.116962	Up	Homo sapiens solute carrier family 44, member 3 (SLC44A3), mRNA [NM_152369]
A_32_P189790	DKFZp779M0652	0.20371	0.000236	0.007604	4.115638	Up	Homo sapiens mRNA; cDNA DKFZp779M0652 (from clone DKFZp779M0652). [BX640618]

A_23_P31536	SSBP1	0.171518	0.000245	0.00779	4.103144	Up	Homo sapiens single-stranded DNA binding protein 1 (SSBP1), mRNA [NM_003143]
A_23_P78518	CEACAM19	0.287267	0.000246	0.007807	4.10146	Up	Homo sapiens cDNA FLJ46368 fis, clone TESTI4051504. [AK128234]
A_23_P430818	HSPC159	0.206972	0.000249	0.007859	4.097424	Up	Homo sapiens galectin-related protein (HSPC159), mRNA [NM_014181]
A_32_P3021	TRIM56	0.178586	0.000254	0.007952	4.090815	Up	tripartite motif containing 56
A_32_P111492	A_32_P111492	0.183244	0.00026	0.008104	4.082983	Up	Unknown
A_23_P408341	TSPYL3	0.25788	0.000261	0.008131	4.080772	Up	Homo sapiens TSPY-like 3 (pseudogene) (TSPYL3) on chromosome 20 [NR_002781]
A_23_P118392	RASD1	0.475907	0.000265	0.00822	4.07565	Up	Homo sapiens RAS, dexamethasone-induced 1 (RASD1), mRNA [NM_016084]
A_24_P911071	A_24_P911071	0.181982	0.000273	0.008433	4.064911	Up	Unknown
A_24_P59327	NRP1	0.212513	0.000276	0.008501	4.061194	Up	Homo sapiens neuropilin-1 soluble isoform 11 (NRP1) mRNA, complete cds, alternatively spliced. [AF280547]
A_23_P123112	ABCB4	0.235841	0.00028	0.008571	4.056995	Up	Homo sapiens ATP-binding cassette, sub-family B (MDR/TAP), member 4 (ABCB4), transcript variant C, mRNA [NM_018850]
A_24_P270890	SPOP	0.16988	0.000285	0.008702	4.050014	Up	Homo sapiens speckle-type POZ protein (SPOP), transcript variant 1, mRNA [NM_001007226]
A_24_P400507	C3orf64	0.21445	0.000288	0.008758	4.046732	Up	Homo sapiens chromosome 3 open reading frame 64 (C3orf64), mRNA [NM_173654]
A_23_P309701	PTPN2	0.214213	0.000289	0.008764	4.045966	Up	Homo sapiens protein tyrosine phosphatase, non-receptor type 2 (PTPN2), transcript variant 1, mRNA [NM_002828]
A_24_P935407	A_24_P935407	0.219108	0.000304	0.009078	4.027511	Up	Unknown
A_24_P246777	Z73417.1	0.200116	0.000308	0.009157	4.023465	Up	TGF beta-inducible nuclear protein 1 (TINP1) pseudogene
A_24_P205263	ESF1	0.196353	0.000308	0.009164	4.022908	Up	Homo sapiens ESF1, nucleolar pre-rRNA processing protein, homolog (S. cerevisiae) (ESF1), mRNA [NM_016649]
A_24_P4426	INPP5F	0.217028	0.000318	0.009356	4.012146	Up	Homo sapiens inositol polyphosphate-5-phosphatase F (INPP5F), transcript variant 1, mRNA [NM_014937]
A_23_P8653	FLJ21062	0.174341	0.000319	0.009381	4.010964	Up	Homo sapiens hypothetical protein FLJ21062 (FLJ21062), mRNA [NM_001039706]
A_32_P170397	ENST00000309874	0.191897	0.000321	0.009445	4.008348	Up	Homo sapiens cDNA FLJ33063 fis, clone TRACH2000047. [AK057625]
A_24_P926648	TIMM17B	0.240737	0.000326	0.009558	4.00293	Up	Homo sapiens translocase of inner mitochondrial membrane 17 homolog B (yeast), mRNA (cDNA clone IMAGE:4696481), partial cds. [BC029446]
A_23_P357571	GSTT2	0.415637	0.000328	0.00961	4.000675	Up	Homo sapiens glutathione S-transferase theta 2 (GSTT2), mRNA [NM_000854]
A_23_P345678	FANCD2	0.174505	0.000331	0.00965	3.997812	Up	Homo sapiens Fanconi anemia, complementation group D2 (FANCD2), transcript variant 1, mRNA [NM_033084]
A_24_P278552	EVA1	0.174154	0.000336	0.009747	3.993054	Up	Homo sapiens epithelial V-like antigen 1 (EVA1), transcript variant 1, mRNA [NM_005797]
A_24_P450104	AK025363	0.212328	0.000338	0.009813	3.990449	Up	Homo sapiens cDNA: FLJ21710 fis, clone COL10087. [AK025363]
A_24_P136471	SLC14A2	0.171341	0.000342	0.009896	3.986027	Up	Homo sapiens cDNA FLJ23656 fis, clone COLF1004, highly similar to Mus musculus urea transporter isoform UTA-3 mRNA. [AK074236]

A_23_P308800	GLS	0.275705	0.000346	0.009958	3.9828	Up	Homo sapiens glutaminase C mRNA, complete cds. [AF158555]
A_24_P856273	A_24_P856273	0.17229	0.00035	0.010046	3.978512	Up	Unknown
A_23_P329452	AGPAT4	0.18663	0.000354	0.010108	3.97464	Up	1-acyl-sn-glycerol-3-phosphate acyltransferase delta (EC 2.3.1.51) (1- AGP acyltransferase 4) (1-AGPAT 4) (Lysophosphatidic acid acyltransferase delta) (LPAAT-delta) (1-acylglycerol-3-phosphate O- acyltransferase 4)....
A_23_P108082	CREB3L3	0.244225	0.000356	0.010164	3.972231	Up	Homo sapiens cAMP responsive element binding protein 3-like 3 (CREB3L3), mRNA [NM_032607]
A_24_P795106	BX090028	0.223903	0.000357	0.010174	3.971254	Up	BX090028 Barstead colon HPLRB7 Homo sapiens cDNA clone IMAGp998B225895 ; IMAGE:2374197, mRNA sequence [BX090028]
A_23_P358995	IBTK	0.17527	0.000358	0.010174	3.970854	Up	Homo sapiens inhibitor of Bruton agammaglobulinemia tyrosine kinase (IBTK), mRNA [NM_015525]
A_23_P158976	ABCC2	0.177246	0.000364	0.010288	3.964462	Up	Homo sapiens ATP-binding cassette, sub-family C (CFTR/MRP), member 2 (ABCC2), mRNA [NM_000392]
A_24_P286868	THC2528446	0.172756	0.000371	0.010399	3.9579	Up	Unknown
A_23_P141770	NAPG	0.177218	0.000374	0.010454	3.95533	Up	Homo sapiens N-ethylmaleimide-sensitive factor attachment protein, gamma (NAPG), mRNA [NM_003826]
A_23_P387856	NEDD4L	0.179329	0.000383	0.010635	3.946412	Up	Homo sapiens neural precursor cell expressed, developmentally down-regulated 4-like (NEDD4L), mRNA [NM_015277]
A_32_P87135	AF116664	0.203963	0.000387	0.010708	3.943304	Up	Homo sapiens PRO1478 mRNA, complete cds. [AF116664]
A_23_P327183	FLJ40606	0.228612	0.000389	0.010732	3.941658	Up	Homo sapiens cDNA FLJ40606 fis, clone THYMU2011939. [AK097925]
A_23_P143127	EML4	0.187271	0.000393	0.010787	3.938111	Up	Homo sapiens echinoderm microtubule associated protein like 4 (EML4), mRNA [NM_019063]
A_24_P943379	A_24_P943379	0.234597	0.000397	0.010891	3.933958	Up	Unknown
A_23_P382584	CHGB	0.188197	0.000398	0.01091	3.932941	Up	Homo sapiens chromogranin B (secretogranin 1) (CHGB), mRNA [NM_001819]
A_32_P101352	CXorf65	0.221288	0.000419	0.011322	3.915226	Up	chromosome X open reading frame 65
A_24_P193093	KLRK1	0.215657	0.000424	0.011385	3.911193	Up	Homo sapiens killer cell lectin-like receptor subfamily K, member 1 (KLRK1), mRNA [NM_007360]
A_23_P92972	TRPC7	0.172592	0.000425	0.0114	3.910482	Up	Homo sapiens transient receptor potential cation channel, subfamily C, member 7 (TRPC7), mRNA [NM_020389]
A_23_P213045	LEF1	0.181127	0.000448	0.011877	3.891229	Up	Homo sapiens lymphoid enhancer-binding factor 1 (LEF1), mRNA [NM_016269]
A_24_P928408	SATB2	0.19187	0.000449	0.011882	3.890844	Up	Homo sapiens cDNA: FLJ21474 fis, clone COL04941. [AK025127]
A_32_P13151	BC071797	0.250164	0.00045	0.011892	3.889639	Up	Homo sapiens cDNA clone IMAGE:4618441, **** WARNING: chimeric clone ****. [BC071797]
A_23_P152992	AK125672	0.178251	0.000471	0.012261	3.873884	Up	Homo sapiens cDNA FLJ43684 fis, clone TBAES2001492. [AK125672]
A_23_P403209	LINC00504	0.204383	0.00049	0.012636	3.859871	Up	long intergenic non-protein coding RNA 504
A_24_P601831	LINC00326	0.197066	0.000494	0.012701	3.85673	Up	long intergenic non-protein coding RNA 326
A_24_P337058	CCNL1	0.172521	0.000499	0.012794	3.853481	Up	Homo sapiens cyclin L1 (CCNL1), mRNA [NM_020307]

A_23_P119763	ABCG5	0.178588	0.0005	0.01282	3.852537	Up	Homo sapiens ATP-binding cassette, sub-family G (WHITE), member 5 (sterolin 1) (ABCG5), mRNA [NM_022436]
A_32_P38323	SERPINB9	0.432658	0.000514	0.013078	3.842634	Up	Homo sapiens serpin peptidase inhibitor, clade B (ovalbumin), member 9 (SERPINB9), mRNA [NM_004155]
A_23_P428298	UNC5CL	0.212108	0.000515	0.013084	3.842125	Up	Homo sapiens unc-5 homolog C (C. elegans)-like (UNC5CL), mRNA [NM_173561]
A_23_P48307	PABPC3	0.191278	0.000517	0.013086	3.841091	Up	Homo sapiens poly(A) binding protein, cytoplasmic 3 (PABPC3), mRNA [NM_030979]
A_24_P215628	DCUN1D2	0.175387	0.000524	0.013231	3.83589	Up	Homo sapiens DCN1, defective in cullin neddylation 1, domain containing 2 (S. cerevisiae) (DCUN1D2), mRNA [NM_001014283]
A_24_P897062	ZNF736	0.172138	0.000533	0.01339	3.830132	Up	zinc finger protein 736
A_24_P85317	CHD2	0.174888	0.000548	0.013649	3.820326	Up	Homo sapiens chromodomain helicase DNA binding protein 2 (CHD2), transcript variant 1, mRNA [NM_001271]
A_24_P221968	AC007342.3	0.192235	0.00055	0.013672	3.819263	Up	M-phase phosphoprotein 10 (U3 small nucleolar ribonucleoprotein)
A_24_P160927	ZNF355P	0.171328	0.000557	0.013761	3.81443	Up	zinc finger protein 355, pseudogene
A_24_P945375	A_24_P945375	0.170218	0.000567	0.013895	3.808384	Up	Unknown
A_23_P101185	NARS	0.202162	0.000582	0.014188	3.79887	Up	Homo sapiens asparaginyl-tRNA synthetase (NARS), mRNA [NM_004539]
A_23_P30687	SERPINB9	0.221763	0.000598	0.014449	3.789265	Up	serpin family B member 9
A_32_P69639	A_32_P69639	0.173287	0.0006	0.014457	3.788256	Up	Unknown
A_24_P943597	PHLDA1	0.304083	0.0006	0.014457	3.788202	Up	Homo sapiens pleckstrin homology-like domain, family A, member 1 (PHLDA1), mRNA [NM_007350]
A_32_P106194	TMEM92	0.289902	0.000601	0.01447	3.78752	Up	Homo sapiens transmembrane protein 92 (TMEM92), mRNA [NM_153229]
A_23_P83939	SYAP1	0.174671	0.000613	0.014628	3.78026	Up	Homo sapiens synapse associated protein 1, SAP47 homolog (Drosophila) (SYAP1), mRNA [NM_032796]
A_23_P392024	ARHGEF10	0.281825	0.000621	0.014771	3.77576	Up	Homo sapiens Rho guanine nucleotide exchange factor (GEF) 10, mRNA (cDNA clone MGC:34901 IMAGE:5103002), complete cds. [BC026965]
A_23_P385790	FAM9C	0.214686	0.00063	0.014873	3.770728	Up	Homo sapiens family with sequence similarity 9, member C (FAM9C), mRNA [NM_174901]
A_32_P203280	THC2656487	0.17157	0.000636	0.014988	3.767495	Up	Unknown
A_24_P4877	ZCRB1	0.205823	0.000651	0.01528	3.759174	Up	Homo sapiens zinc finger CCHC-type and RNA binding motif 1 (ZCRB1), mRNA [NM_033114]
A_23_P212535	CHMP2B	0.17229	0.000651	0.015284	3.758874	Up	Homo sapiens chromatin modifying protein 2B (CHMP2B), mRNA [NM_014043]
A_23_P16370	RFPL4AP1	0.197942	0.000673	0.015619	3.747285	Up	ret finger protein like 4A pseudogene 1
A_24_P372959	CCDC91	0.217616	0.00069	0.015855	3.738114	Up	Homo sapiens coiled-coil domain containing 91 (CCDC91), mRNA [NM_018318]
A_24_P346859	TMEM273	0.22796	0.000694	0.015906	3.735984	Up	transmembrane protein 273
A_24_P204257	A_24_P204257	0.169765	0.000704	0.016062	3.731083	Up	Unknown
A_24_P934435	AF086077	0.325145	0.000707	0.016117	3.729472	Up	Homo sapiens full length insert cDNA clone YZ60H05. [AF086077]

A_23_P41025	GNL3	0.20641	0.000731	0.0165	3.717508	Up	Homo sapiens guanine nucleotide binding protein-like 3 (nucleolar) (GNL3), transcript variant 1, mRNA [NM_014366]
A_24_P312072	LYRM4	0.186253	0.000743	0.016676	3.711767	Up	Homo sapiens LYR motif containing 4 (LYRM4), mRNA [NM_020408]
A_23_P52207	BAMBI	0.202463	0.000746	0.016722	3.710376	Up	Homo sapiens BMP and activin membrane-bound inhibitor homolog (Xenopus laevis) (BAMBI), mRNA [NM_012342]
A_23_P60373	IFNW1	0.203441	0.00075	0.016777	3.708467	Up	Homo sapiens interferon, omega 1 (IFNW1), mRNA [NM_002177]
A_32_P68148	ZNF738	0.17066	0.000762	0.016938	3.702867	Up	PREDICTED: Homo sapiens hypothetical protein LOC148203 (LOC148203), misc RNA [XR_015756]
A_24_P287613	BC066984	0.17836	0.000773	0.017145	3.697417	Up	Homo sapiens cDNA clone IMAGE:4826623. [BC066984]
A_24_P337796	STK17A	0.190263	0.000774	0.017145	3.697144	Up	Homo sapiens serine/threonine kinase 17a (STK17A), mRNA [NM_004760]
A_24_P176733	CCDC47	0.182661	0.000794	0.017448	3.688037	Up	Homo sapiens coiled-coil domain containing 47 (CCDC47), mRNA [NM_020198]
A_23_P215956	MYC	0.224151	0.000811	0.017732	3.680279	Up	Homo sapiens v-myc myelocytomatosis viral oncogene homolog (avian) (MYC), mRNA [NM_002467]
A_23_P23924	CAPN2	0.220031	0.000817	0.017808	3.677973	Up	Homo sapiens calpain 2, (m/II) large subunit (CAPN2), mRNA [NM_001748]
A_23_P125705	NAP1L2	0.174532	0.000827	0.017947	3.673284	Up	Homo sapiens nucleosome assembly protein 1-like 2 (NAP1L2), mRNA [NM_021963]
A_32_P109604	ENST00000369381	0.265661	0.00083	0.017982	3.672247	Up	Homo sapiens cDNA clone MGC:23978 IMAGE:4768142, complete cds. [BC022380]
A_23_P214876	JARID2	0.249206	0.000841	0.018189	3.667334	Up	Homo sapiens jumonji, AT rich interactive domain 2 (JARID2), mRNA [NM_004973]
A_24_P261691	HIST4H4	0.186979	0.000842	0.018195	3.667019	Up	Homo sapiens histone cluster 4, H4, mRNA (cDNA clone MGC:24116 IMAGE:4619662), complete cds. [BC020884]
A_23_P52499	SLC18A2	0.183081	0.000851	0.018356	3.663095	Up	Homo sapiens solute carrier family 18 (vesicular monoamine), member 2 (SLC18A2), mRNA [NM_003054]
A_24_P910230	THC2735775	0.209482	0.000903	0.019145	3.641682	Up	ALU8_HUMAN (P39195) Alu subfamily SX sequence contamination warning entry, partial (6%) [THC2735775]
A_23_P5761	NFE2L2	0.184034	0.000918	0.019385	3.636048	Up	Homo sapiens nuclear factor (erythroid-derived 2)-like 2 (NFE2L2), mRNA [NM_006164]
A_23_P157795	CTNNAL1	0.177319	0.000927	0.019491	3.632345	Up	Homo sapiens catenin (cadherin-associated protein), alpha-like 1 (CTNNAL1), mRNA [NM_003798]
A_24_P921343	SCAF11	0.252518	0.001029	0.021051	3.594694	Up	SR-related CTD associated factor 11
A_23_P7185	BRDG1	0.176049	0.001034	0.021098	3.593046	Up	Homo sapiens BCR downstream signaling 1 (BRDG1), mRNA [NM_012108]
A_23_P43415	HSD17B3	0.197183	0.001059	0.021491	3.584249	Up	Homo sapiens hydroxysteroid (17-beta) dehydrogenase 3 (HSD17B3), mRNA [NM_000197]
A_32_P87872	IMMP2L	0.17367	0.001068	0.021586	3.581312	Up	Homo sapiens IMP2 inner mitochondrial membrane peptidase-like (S. cerevisiae) (IMMP2L), mRNA [NM_032549]
A_24_P668821	MIS18BP1	0.233136	0.00107	0.021609	3.580379	Up	MIS18 binding protein 1
A_24_P531720	A_24_P531720	0.228527	0.001072	0.021624	3.579959	Up	Unknown
A_32_P18470	TCEAL5	0.214652	0.001096	0.021935	3.571768	Up	Homo sapiens transcription elongation factor A (SII)-like 5 (TCEAL5), mRNA [NM_001012979]
A_24_P59239	A_24_P59239	0.258651	0.00112	0.022297	3.563896	Up	Unknown

A_24_P101128	DMXL1	0.246639	0.001131	0.022417	3.560449	Up	Homo sapiens Dmx-like 1 (DMXL1), mRNA [NM_005509]
A_24_P336931	KIAA1641	0.181662	0.001131	0.022417	3.560355	Up	Homo sapiens KIAA1641 (KIAA1641), mRNA [NM_025190]
A_23_P88119	HSPH1	0.278216	0.001138	0.02246	3.558312	Up	Homo sapiens heat shock 105kDa/110kDa protein 1 (HSPH1), mRNA [NM_006644]
A_23_P415510	LAD1	0.202096	0.001138	0.02246	3.558266	Up	Homo sapiens ladinin 1 (LAD1), mRNA [NM_005558]
A_23_P157766	C9orf68	0.241385	0.001163	0.022906	3.550252	Up	Homo sapiens chromosome 9 open reading frame 68 (C9orf68), mRNA [NM_001039395]
A_32_P65303	FAM49B	0.173058	0.001169	0.022974	3.548432	Up	family with sequence similarity 49 member B
A_24_P467568	BF801387	0.186426	0.001182	0.023133	3.544371	Up	BF801387 IL5-CI0001-251000-187-c10 CI0001 Homo sapiens cDNA, mRNA sequence [BF801387]
A_32_P75867	THC2691419	0.277386	0.001201	0.023396	3.53875	Up	ALU1_HUMAN (P39188) Alu subfamily J sequence contamination warning entry, partial (5%) [THC2691419]
A_23_P207940	PTPN2	0.246063	0.001202	0.023406	3.538418	Up	Homo sapiens protein tyrosine phosphatase, non-receptor type 2 (PTPN2), transcript variant 2, mRNA [NM_080422]
A_23_P89145	ZNF83	0.188104	0.001207	0.02348	3.536894	Up	Homo sapiens zinc finger protein 83 (ZNF83), mRNA [NM_018300]
A_24_P334248	PLCH1	0.169878	0.001215	0.023566	3.53451	Up	Homo sapiens phospholipase C, eta 1 (PLCH1), mRNA [NM_014996]
A_24_P82094	BAG5	0.214854	0.001219	0.023602	3.533356	Up	Homo sapiens BCL2-associated athanogene 5 (BAG5), transcript variant 1, mRNA [NM_001015049]
A_24_P12438	NCOA7	0.250042	0.001223	0.023658	3.532036	Up	Homo sapiens nuclear receptor coactivator 7 (NCOA7), mRNA [NM_181782]
A_24_P233256	ZNF649	0.230318	0.001231	0.023701	3.529551	Up	Homo sapiens zinc finger protein 649 (ZNF649), mRNA [NM_023074]
A_23_P91619	MIF	0.199534	0.001232	0.023701	3.529454	Up	Homo sapiens macrophage migration inhibitory factor (glycosylation-inhibiting factor) (MIF), mRNA [NM_002415]
A_24_P914649	CLNS1A	0.263167	0.001241	0.023808	3.52674	Up	chloride nucleotide-sensitive channel 1A
A_32_P117322	AV707592	0.234357	0.001248	0.023906	3.524558	Up	AV707592 ADB Homo sapiens cDNA clone ADBAJA03 5', mRNA sequence [AV707592]
A_24_P105648	BX111927	0.199248	0.001252	0.02395	3.523416	Up	BX111927 Soares_multiple_sclerosis_2NbHMSP Homo sapiens cDNA clone IMAGp998G05619, mRNA sequence [BX111927]
A_23_P432598	CHMP4C	0.29256	0.001311	0.024736	3.506728	Up	Homo sapiens chromatin modifying protein 4C (CHMP4C), mRNA [NM_152284]
A_23_P109143	PRNP	0.191672	0.001324	0.024871	3.503056	Up	Homo sapiens prion protein (p27-30) (Creutzfeldt-Jakob disease, Gerstmann-Strausler-Scheinker syndrome, fatal familial insomnia) (PRNP), transcript variant 1, mRNA [NM_000311]
A_24_P501285	AL080072	0.191271	0.001331	0.024928	3.501304	Up	Homo sapiens mRNA; cDNA DKFZp564M0616 (from clone DKFZp564M0616). [AL080072]
A_23_P202361	HPS1	0.173832	0.00135	0.0252	3.495938	Up	Homo sapiens Hermansky-Pudlak syndrome 1 (HPS1), transcript variant 3, mRNA [NM_182639]
A_24_P923591	PPIL2	0.28182	0.001362	0.025392	3.492658	Up	peptidylprolyl isomerase like 2
A_24_P175188	SAMD9	0.169739	0.00137	0.025485	3.490615	Up	Homo sapiens sterile alpha motif domain containing 9 (SAMD9), mRNA [NM_017654]
A_23_P253622	KIAA1641	0.372215	0.001371	0.025485	3.490332	Up	Homo sapiens cDNA: FLJ21281 fis, clone COL01904. [AK024934]
A_23_P137984	S100A10	0.262634	0.001379	0.025577	3.488361	Up	Homo sapiens S100 calcium binding protein A10 (S100A10), mRNA [NM_002966]

A_32_P120257	MEG8	0.252142	0.001385	0.025627	3.486745	Up	maternally expressed 8, small nucleolar RNA host gene
A_23_P122443	HIST1H1C	0.206489	0.001428	0.026223	3.475579	Up	Homo sapiens histone cluster 1, H1c (HIST1H1C), mRNA [NM_005319]
A_23_P258221	ABCC5	0.205066	0.001432	0.026258	3.474365	Up	Homo sapiens ATP-binding cassette, sub-family C (CFTR/MRP), member 5 (ABCC5), transcript variant 1, mRNA [NM_005688]
A_23_P209195	C19orf12	0.169478	0.001442	0.026335	3.471927	Up	Homo sapiens chromosome 19 open reading frame 12 (C19orf12), transcript variant 2, mRNA [NM_031448]
A_32_P209230	CITED4	0.176015	0.001466	0.026637	3.465768	Up	Homo sapiens Cbp/p300-interacting transactivator, with Glu/Asp-rich carboxy-terminal domain, 4 (CITED4), mRNA [NM_133467]
A_32_P170736	CCDC71L	0.234661	0.001476	0.026729	3.463407	Up	coiled-coil domain containing 71 like
A_32_P58215	CD84	0.227648	0.001485	0.026841	3.461235	Up	SLAM family member 5 precursor (Signaling lymphocytic activation molecule 5) (Leukocyte differentiation antigen CD84) (CD84 antigen) (Cell surface antigen MAX.3) (Hly9-beta). [Source:Uniprot/SWISSPROT;Acc:Q9UIB8] [ENST00000368054]
A_23_P92370	MAP9	0.294897	0.001515	0.02726	3.453749	Up	Homo sapiens microtubule-associated protein 9 (MAP9), mRNA [NM_001039580]
A_23_P30315	TRIM7	0.205852	0.001522	0.027349	3.452102	Up	Homo sapiens tripartite motif-containing 7 (TRIM7), transcript variant 6, mRNA [NM_033342]
A_23_P305335	RBM8A	0.197255	0.00155	0.027622	3.445415	Up	Homo sapiens RNA binding motif protein 8A, mRNA (cDNA clone IMAGE:4687764), complete cds. [BC017770]
A_24_P418086	RBM34	0.18145	0.001566	0.027824	3.441693	Up	Homo sapiens RNA binding motif protein 34 (RBM34), mRNA [NM_015014]
A_32_P39944	AK095791	0.177825	0.001575	0.02792	3.439683	Up	Homo sapiens cDNA FLJ38472 fis, clone FEBRA2022148. [AK095791]
A_24_P920418	STXBP3	0.261086	0.001593	0.02814	3.435429	Up	Homo sapiens syntaxin binding protein 3, mRNA (cDNA clone IMAGE:5219755), partial cds. [BC028028]
A_23_P57856	BCL6	0.21144	0.001593	0.02814	3.43537	Up	Homo sapiens B-cell CLL/lymphoma 6 (zinc finger protein 51) (BCL6), transcript variant 2, mRNA [NM_138931]
A_24_P178154	A_24_P178154	0.188013	0.001642	0.028749	3.424337	Up	Unknown
A_24_P175909	MARS	0.270565	0.001653	0.028875	3.421851	Up	Homo sapiens methionyl-tRNA synthetase (MARS), mRNA [NM_004990]
A_23_P389371	MRGPRX3	0.255211	0.001654	0.02889	3.421498	Up	Homo sapiens MAS-related GPR, member X3 (MRGPRX3), mRNA [NM_054031]
A_24_P298616	A_24_P298616	0.21683	0.00167	0.029091	3.418015	Up	Unknown
A_24_P153853	TRIM37	0.175626	0.001686	0.029299	3.41461	Up	Homo sapiens tripartite motif-containing 37 (TRIM37), transcript variant 2, mRNA [NM_001005207]
A_23_P82550	STK17A	0.239672	0.001692	0.029365	3.413308	Up	Homo sapiens serine/threonine kinase 17a (STK17A), mRNA [NM_004760]
A_32_P150086	LOC644339	0.270156	0.001705	0.029511	3.410396	Up	PREDICTED: Homo sapiens similar to ankyrin repeat domain 20A (LOC644339), mRNA [XR_016677]
A_32_P177953	GCLM	0.21502	0.001739	0.029991	3.403214	Up	Glutamate--cysteine ligase regulatory subunit (EC 6.3.2.2) (Gamma- glutamylcysteine synthetase) (Gamma-ECS) (GCS light chain) (Glutamate--cysteine ligase modifier subunit). [Source:Uniprot/SWISSPROT;Acc:P48507] [ENST00000370238]

A_23_P328298	ODF2L	0.183788	0.001754	0.030235	3.399915	Up	Homo sapiens outer dense fiber of sperm tails 2-like (ODF2L), transcript variant 1, mRNA [NM_020729]
A_32_P107797	LOC730753	0.174883	0.001756	0.030259	3.39946	Up	PREDICTED: Homo sapiens hypothetical protein LOC730753 (LOC730753), mRNA [XR_015400]
A_23_P78958	CAPS	0.214766	0.001757	0.030263	3.399266	Up	Homo sapiens calcyphosine (CAPS), transcript variant 1, mRNA [NM_004058]
A_23_P14255	GUCY1B2	0.382091	0.001767	0.030402	3.39726	Up	guanylate cyclase 1 soluble subunit beta 2 (pseudogene)
A_24_P928112	A_24_P928112	0.18222	0.001805	0.030807	3.389508	Up	Unknown
A_24_P294851	TRIM38	0.215415	0.001805	0.030807	3.389468	Up	Homo sapiens tripartite motif-containing 38 (TRIM38), mRNA [NM_006355]
A_32_P219520	TNFAIP8	0.207174	0.001806	0.030807	3.389252	Up	Homo sapiens tumor necrosis factor, alpha-induced protein 8 (TNFAIP8), transcript variant 1, mRNA [NM_014350]
A_23_P23765	ITGB3BP	0.209733	0.001852	0.031294	3.379979	Up	Homo sapiens integrin beta 3 binding protein (beta3-endonexin) (ITGB3BP), mRNA [NM_014288]
A_23_P409648	SP140L	0.216669	0.001852	0.031294	3.379851	Up	SP140 nuclear body protein like
A_32_P806841	ARL4A	0.209973	0.001864	0.031434	3.377574	Up	Homo sapiens ADP-ribosylation factor-like 4A (ARL4A), transcript variant 1, mRNA [NM_005738]
A_23_P2216	KRR1	0.193805	0.001874	0.031541	3.375596	Up	Homo sapiens KRR1, small subunit (SSU) processome component, homolog (yeast) (KRR1), mRNA [NM_007043]
A_24_P937445	TAOK1	0.201086	0.001882	0.031616	3.37392	Up	Homo sapiens cDNA FLJ14314 fis, clone PLACE3000350, weakly similar to SERINE/THREONINE-PROTEIN KINASE SULU (EC 2.7.1.-). [AK024376]
A_32_P121549	BC034319	0.211187	0.001886	0.031658	3.373208	Up	Homo sapiens cDNA clone IMAGE:4837650. [BC034319]
A_23_P139434	PRB2	0.233041	0.001946	0.032302	3.361627	Up	Homo sapiens proline-rich protein BstNI subfamily 2 (PRB2), mRNA [NM_006248]
A_23_P156957	NCOA7	0.266736	0.001946	0.032302	3.361606	Up	Homo sapiens nuclear receptor coactivator 7 (NCOA7), mRNA [NM_181782]
A_23_P11119	A_23_P11119	0.175289	0.001969	0.032588	3.357237	Up	Unknown
A_24_P247006	DENND2C	0.187826	0.001972	0.032588	3.356622	Up	Homo sapiens DENN/MADD domain containing 2C (DENND2C), mRNA [NM_198459]
A_24_P192485	TNFRSF11B	0.214531	0.00207	0.033533	3.338695	Up	Homo sapiens tumor necrosis factor receptor superfamily, member 11b (osteoprotegerin) (TNFRSF11B), mRNA [NM_002546]
A_23_P252306	ID1	0.227538	0.002077	0.033613	3.337472	Up	Homo sapiens inhibitor of DNA binding 1, dominant negative helix-loop-helix protein (ID1), transcript variant 1, mRNA [NM_002165]
A_32_P855872	AK094117	0.195064	0.002089	0.033722	3.335298	Up	Homo sapiens cDNA FLJ36798 fis, clone ADRGL2007283. [AK094117]
A_23_P17998	HES1	0.238216	0.002094	0.033748	3.334502	Up	Homo sapiens hairy and enhancer of split 1, (Drosophila) (HES1), mRNA [NM_005524]
A_32_P8120	GNL3	0.199733	0.002095	0.033748	3.334158	Up	Homo sapiens guanine nucleotide binding protein-like 3 (nucleolar) (GNL3), transcript variant 1, mRNA [NM_014366]
A_23_P410862	ENST00000360738	0.189297	0.002096	0.033748	3.334075	Up	Homo sapiens partial mRNA for hypothetical protein (ORF1), clone pT-Adv JuaX22. [AJ276510]
A_23_P343221	LILRB1	0.197399	0.002096	0.033748	3.333984	Up	Homo sapiens leukocyte immunoglobulin-like receptor, subfamily B (with TM and ITIM domains), member 1 (LILRB1), transcript variant 1, mRNA [NM_006669]

A_32_P231143	A_32_P231143	0.28322	0.002112	0.03391	3.331188	Up	Unknown
A_24_P186124	MTERFD2	0.176904	0.002113	0.033914	3.331	Up	Homo sapiens MTERF domain containing 2 (MTERFD2), mRNA [NM_182501]
A_23_P163737	OR2C1	0.170508	0.002129	0.034092	3.328182	Up	Homo sapiens olfactory receptor, family 2, subfamily C, member 1 (OR2C1), mRNA [NM_012368]
A_23_P98410	CD3G	0.293182	0.002148	0.034259	3.324891	Up	Homo sapiens CD3g molecule, gamma (CD3-TCR complex) (CD3G), mRNA [NM_000073]
A_23_P317591	SEMA3A	0.237015	0.002159	0.03432	3.323099	Up	Homo sapiens sema domain, immunoglobulin domain (Ig), short basic domain, secreted, (semaphorin) 3A (SEMA3A), mRNA [NM_006080]
A_23_P171296	MPP1	0.198732	0.002168	0.034444	3.321475	Up	Homo sapiens membrane protein, palmitoylated 1, 55kDa (MPP1), mRNA [NM_002436]
A_23_P320070	CARD14	0.170418	0.002207	0.034774	3.314851	Up	Homo sapiens caspase recruitment domain family, member 14 (CARD14), transcript variant 2, mRNA [NM_052819]
A_24_P324749	KIAA1641	0.20399	0.002221	0.034931	3.312448	Up	Homo sapiens CLL-associated antigen KW-1 splice variant 2 mRNA, partial cds; alternatively spliced. [AF432209]
A_24_P941450	A_24_P941450	0.174564	0.002222	0.034931	3.312318	Up	Unknown
A_23_P354151	ITK	0.230497	0.002246	0.035171	3.308395	Up	Homo sapiens IL2-inducible T-cell kinase (ITK), mRNA [NM_005546]
A_24_P137563	SCLT1	0.215494	0.00225	0.03522	3.30774	Up	Homo sapiens sodium channel and clathrin linker 1 (SCLT1), mRNA [NM_144643]
A_32_P177955	STX17-AS1	0.170455	0.002256	0.035247	3.306737	Up	STX17 antisense RNA 1
A_23_P8649	FAM133B	0.191399	0.002286	0.035542	3.301789	Up	Homo sapiens hypothetical protein MGC40405 (MGC40405), transcript variant 1, mRNA [NM_152789]
A_23_P44155	CD96	0.269008	0.002318	0.035878	3.296531	Up	Homo sapiens CD96 molecule (CD96), transcript variant 1, mRNA [NM_198196]
A_24_P355816	DRAM	0.207166	0.00235	0.036194	3.291524	Up	Homo sapiens damage-regulated autophagy modulator (DRAM), mRNA [NM_018370]
A_32_P77102	LINC00892	0.26887	0.002352	0.036194	3.29121	Up	long intergenic non-protein coding RNA 892
A_32_P144634	A_32_P144634	0.182628	0.00236	0.036277	3.289937	Up	Unknown
A_24_P922979	A_24_P922979	0.198217	0.002371	0.036393	3.288182	Up	Unknown
A_23_P132454	CCNL1	0.179758	0.002545	0.038195	3.261674	Up	Homo sapiens cyclin L1 (CCNL1), mRNA [NM_020307]
A_23_P391586	TPM1	0.183573	0.002551	0.038272	3.260782	Up	Homo sapiens tropomyosin 1 (alpha) (TPM1), transcript variant 3, mRNA [NM_001018004]
A_23_P326760	MYRIP	0.171079	0.002569	0.03841	3.258201	Up	Homo sapiens myosin VIIA and Rab interacting protein (MYRIP), mRNA [NM_015460]
A_23_P68601	CST7	0.224015	0.002614	0.038942	3.251679	Up	Homo sapiens cystatin F (leukocystatin) (CST7), mRNA [NM_003650]
A_23_P32253	NFIL3	0.183352	0.002619	0.03897	3.251001	Up	Homo sapiens nuclear factor, interleukin 3 regulated (NFIL3), mRNA [NM_005384]
A_23_P153745	IFI30	0.180373	0.002634	0.039112	3.248792	Up	Homo sapiens interferon, gamma-inducible protein 30 (IFI30), mRNA [NM_006332]
A_23_P101671	ATCAY	0.187284	0.002636	0.039112	3.2485	Up	Homo sapiens ataxia, cerebellar, Cayman type (caytaxin) (ATCAY), mRNA [NM_033064]
A_32_P72758	MCTP2	0.267093	0.002637	0.039112	3.248338	Up	multiple C2 and transmembrane domain containing 2

A_24_P364296	STX2	0.172079	0.002639	0.039112	3.248117	Up	Homo sapiens syntaxin 2 (STX2), transcript variant 1, mRNA [NM_001980]
A_23_P84154	ARHGAP15	0.174474	0.002649	0.039166	3.24669	Up	Homo sapiens Rho GTPase activating protein 15 (ARHGAP15), mRNA [NM_018460]
A_23_P20660	DIRAS2	0.268503	0.002653	0.039196	3.246121	Up	Homo sapiens DIRAS family, GTP-binding RAS-like 2 (DIRAS2), mRNA [NM_017594]
A_23_P417475	STK31	0.178298	0.002666	0.039327	3.244191	Up	Homo sapiens cDNA FLJ32868 fis, clone TESTI2003781. [AK057430]
A_23_P10025	NELL2	0.310914	0.002693	0.039625	3.240412	Up	Homo sapiens NEL-like 2 (chicken) (NELL2), mRNA [NM_006159]
A_23_P332413	SLFN13	0.2079	0.00274	0.04009	3.233965	Up	Homo sapiens schlafen family member 13 (SLFN13), mRNA [NM_144682]
A_24_P740549	ARHGEF26-AS1	0.184577	0.002741	0.04009	3.233787	Up	ARHGEF26 antisense RNA 1
A_24_P167473	ARPC3	0.234036	0.002749	0.040145	3.232693	Up	Homo sapiens actin related protein 2/3 complex, subunit 3, 21kDa (ARPC3), mRNA [NM_005719]
A_23_P436284	SLC51B	0.46887	0.002757	0.040204	3.231649	Up	solute carrier family 51 beta subunit
A_23_P168188	SYTL3	0.201413	0.002765	0.04027	3.230584	Up	Homo sapiens synaptotagmin-like 3 (SYTL3), mRNA [NM_001009991]
A_23_P94397	OMD	0.25064	0.00278	0.040435	3.228516	Up	Homo sapiens osteomodulin (OMD), mRNA [NM_005014]
A_32_P17343	THC2667726	0.295515	0.002813	0.040711	3.224086	Up	zinc finger protein 728
A_24_P358868	ZNF728	0.193957	0.002833	0.040916	3.221409	Up	PREDICTED: Homo sapiens similar to Zinc finger protein 208 (LOC388523), mRNA [XR_017338]
A_32_P148627	AX775927	0.182678	0.002904	0.041708	3.212071	Up	Sequence 197 from Patent WO03048202. [AX775927]
A_23_P53856	PFAAP5	0.206739	0.00296	0.042211	3.204925	Up	Homo sapiens phosphonoformate immuno-associated protein 5 (PFAAP5), mRNA [NM_014887]
A_24_P45481	AKT3	0.218529	0.002983	0.042456	3.20195	Up	Homo sapiens v-akt murine thymoma viral oncogene homolog 3 (protein kinase B, gamma) (AKT3), transcript variant 1, mRNA [NM_005465]
A_32_P89709	TPM1	0.268594	0.003032	0.042962	3.19578	Up	Homo sapiens tropomyosin 1 (alpha) (TPM1), transcript variant 3, mRNA [NM_001018004]
A_32_P163036	C2orf74	0.169412	0.003055	0.043157	3.19303	Up	chromosome 2 open reading frame 74
A_23_P398491	CCDC32	0.169859	0.00307	0.043249	3.191184	Up	coiled-coil domain containing 32
A_23_P122210	LINC01949	0.21723	0.003073	0.04328	3.190784	Up	long intergenic non-protein coding RNA 1949
A_24_P162597	AF116705	0.236523	0.00313	0.043854	3.18384	Up	Homo sapiens PRO2489 mRNA, complete cds. [AF116705]
A_32_P62276	LINC00943	0.195151	0.003197	0.044516	3.175744	Up	long intergenic non-protein coding RNA 943
A_23_P501183	NSUN5C	0.202183	0.003343	0.045887	3.158915	Up	Homo sapiens NOL1/NOP2/Sun domain family, member 5C (NSUN5C), transcript variant 1, mRNA [NM_032158]
A_23_P3552	LOC730092	0.214883	0.003348	0.045891	3.158343	Up	Homo sapiens RRN3 RNA polymerase I transcription factor homolog (S. cerevisiae) pseudogene (LOC730092) on chromosome 16 [NR_003370]
A_23_P356216	ARHGEF10	0.237527	0.003392	0.046288	3.153389	Up	Homo sapiens Rho guanine nucleotide exchange factor (GEF) 10, mRNA (cDNA clone MGC:34901 IMAGE:5103002), complete cds. [BC026965]

A_23_P147025	RAB33A	0.262208	0.003422	0.046556	3.149973	Up	Homo sapiens RAB33A, member RAS oncogene family (RAB33A), mRNA [NM_004794]
A_24_P772004	AA005355	0.200844	0.003437	0.046671	3.148367	Up	AA005355 zh97a03.s1 Soares_fetal_liver_spleen_1NFLS_S1 Homo sapiens cDNA clone IMAGE:429196 3' similar to contains Alu repetitive element;, mRNA sequence [AA005355]
A_23_P154155	MFSD9	0.185247	0.003442	0.0467	3.147793	Up	Homo sapiens major facilitator superfamily domain containing 9 (MFSD9), mRNA [NM_032718]
A_24_P180151	ROBO2	0.174812	0.003452	0.046773	3.146653	Up	Homo sapiens roundabout, axon guidance receptor, homolog 2 (Drosophila) (ROBO2), mRNA [NM_002942]
A_23_P74778	C1orf54	0.206926	0.003456	0.046786	3.146299	Up	Homo sapiens chromosome 1 open reading frame 54 (C1orf54), mRNA [NM_024579]
A_32_P499005	LINC01658	0.169919	0.003497	0.047254	3.141733	Up	long intergenic non-protein coding RNA 1658
A_24_P324581	KIAA1466	0.219854	0.003508	0.047312	3.140593	Up	Homo sapiens mRNA for KIAA1466 protein, partial cds. [AB040899]
A_32_P235358	AK124173	0.233351	0.003572	0.047957	3.133653	Up	Homo sapiens cDNA FLJ42179 fis, clone THYMU2030796. [AK124173]
A_23_P164210	TBC1D3	0.183026	0.00363	0.048334	3.12755	Up	Homo sapiens TBC1 domain family, member 3 (TBC1D3), mRNA [NM_032258]
A_32_P761454	C21orf74	0.170907	0.003702	0.049058	3.120043	Up	Homo sapiens clone qd65g07 PRED16 protein (PRED16) mRNA, complete cds. [AY077696]
A_23_P156826	C6orf105	0.324876	0.00374	0.04938	3.116236	Up	Homo sapiens chromosome 6 open reading frame 105 (C6orf105), mRNA [NM_032744]
A_24_P924183	MYO10	0.198391	0.003766	0.049489	3.113519	Up	Homo sapiens myosin X, mRNA (cDNA clone IMAGE:6164790), complete cds. [BC108736]
A_23_P72680	IFT81	0.202022	0.003767	0.049489	3.113489	Up	Homo sapiens intraflagellar transport 81 homolog (Chlamydomonas) (IFT81), transcript variant 1, mRNA [NM_014055]
A_32_P195850	DPY19L2	0.196234	0.00377	0.049492	3.113159	Up	Homo sapiens dpy-19-like 2 (C. elegans) (DPY19L2), mRNA [NM_173812]
A_24_P920388	LINC02361	0.171127	0.00381	0.049835	3.1091	Up	long intergenic non-protein coding RNA 2361
A_23_P256504	AMBP	-0.75461	5.74E-23	2.35E-18	-24.4448	Down	Homo sapiens alpha-1-microglobulin/bikunin precursor (AMBP), mRNA [NM_001633]
A_24_P113109	UBB	-0.60099	1.17E-22	2.4E-18	-23.9037	Down	Homo sapiens ubiquitin B (UBB), mRNA [NM_018955]
A_24_P681218	THC2544911	-0.84428	1.14E-21	1.56E-17	-22.2427	Down	MUSEFTU elongation factor Tu {Mus musculus} (exp=-1; wgp=0; cg=0), partial (37%) [THC2544911]
A_23_P360213	ND3	-0.75172	3.67E-21	3.76E-17	-21.4311	Down	NADH-ubiquinone oxidoreductase chain 3 (EC 1.6.5.3) (NADH dehydrogenase subunit 3). [Source:Uniprot/SWISSPROT;Acc:P03897] [ENST00000361227]
A_23_P321892	HRG	-0.77392	5.53E-21	4.53E-17	-21.1527	Down	Homo sapiens histidine-rich glycoprotein (HRG), mRNA [NM_000412]
A_23_P415974	ALDOB	-0.84706	2.14E-20	1.39E-16	-20.2549	Down	Homo sapiens aldolase B, fructose-bisphosphate (ALDOB), mRNA [NM_000035]
A_23_P257834	ALB	-0.80176	2.37E-20	1.39E-16	-20.1895	Down	Homo sapiens albumin (ALB), mRNA [NM_000477]
A_23_P167349	GC	-0.54705	3.19E-20	1.54E-16	-19.9968	Down	Homo sapiens group-specific component (vitamin D binding protein) (GC), mRNA [NM_000583]
A_32_P206123	PLG	-0.58561	3.37E-20	1.54E-16	-19.9606	Down	Homo sapiens plasminogen (PLG), mRNA [NM_000301]

A_23_P121926	SEPP1	-0.75215	8.52E-20	3.49E-16	-19.3721	Down	Homo sapiens selenoprotein P, plasma, 1 (SEPP1), mRNA [NM_005410]
A_23_P36745	ALDH2	-0.82101	9.65E-20	3.6E-16	-19.2942	Down	Homo sapiens aldehyde dehydrogenase 2 family (mitochondrial) (ALDH2), nuclear gene encoding mitochondrial protein, mRNA [NM_000690]
A_24_P763243	EEF1A1	-0.74934	1.22E-19	4.16E-16	-19.1493	Down	Homo sapiens eukaryotic translation elongation factor 1 alpha 1 (EEF1A1), mRNA [NM_001402]
A_23_P114626	SERPINC1	-0.67403	1.84E-19	5.81E-16	-18.8939	Down	Homo sapiens serpin peptidase inhibitor, clade C (antithrombin), member 1 (SERPINC1), mRNA [NM_000488]
A_23_P101208	CYB5A	-0.78339	2.47E-19	6.92E-16	-18.7136	Down	Homo sapiens cytochrome b5 type A (microsomal) (CYB5A), transcript variant 2, mRNA [NM_001914]
A_24_P179351	TPT1	-0.9151	2.53E-19	6.92E-16	-18.6992	Down	Homo sapiens tumor protein, translationally-controlled 1 (TPT1), mRNA [NM_003295]
A_32_P96213	TPT1	-0.62826	4.9E-19	1.25E-15	-18.3011	Down	Homo sapiens tumor protein, translationally-controlled 1 (TPT1), mRNA [NM_003295]
A_23_P218111	SERPINA1	-0.67697	8.17E-19	1.97E-15	-17.9971	Down	Homo sapiens serpin peptidase inhibitor, clade A (alpha-1 antiproteinase, antitrypsin), member 1 (SERPINA1), transcript variant 2, mRNA [NM_001002236]
A_24_P179336	AY029066	-0.99157	9.95E-19	2.27E-15	-17.8809	Down	Homo sapiens Humanin (HN1) mRNA, complete cds. [AY029066]
A_23_P101407	C3	-0.65798	1.77E-18	3.65E-15	-17.5446	Down	Homo sapiens complement component 3 (C3), mRNA [NM_000064]
A_23_P212258	KNG1	-0.54305	1.78E-18	3.65E-15	-17.5419	Down	Homo sapiens kininogen 1 (KNG1), mRNA [NM_000893]
A_24_P302249	APOA2	-0.67049	2.29E-18	4.46E-15	-17.3981	Down	Homo sapiens apolipoprotein A-II (APOA2), mRNA [NM_001643]
A_23_P30693	PLG	-0.59563	4.77E-18	8.89E-15	-16.9801	Down	Homo sapiens plasminogen (PLG), mRNA [NM_000301]
A_24_P213783	RPL31	-0.50539	6.58E-18	1.17E-14	-16.8	Down	Homo sapiens ribosomal protein L31 (RPL31), mRNA [NM_000993]
A_23_P79562	FABP1	-0.80957	1.01E-17	1.72E-14	-16.5647	Down	Homo sapiens fatty acid binding protein 1, liver (FABP1), mRNA [NM_001443]
A_32_P220307	RPL39	-0.35932	1.33E-17	2.18E-14	-16.4116	Down	Homo sapiens ribosomal protein L39 (RPL39), mRNA [NM_001000]
A_32_P231391	LDHA	-0.49006	1.62E-17	2.55E-14	-16.3038	Down	Homo sapiens lactate dehydrogenase A (LDHA), mRNA [NM_005566]
A_23_P109881	ITIH4	-0.38638	2.35E-17	3.57E-14	-16.1015	Down	Homo sapiens inter-alpha (globulin) inhibitor H4 (plasma Kallikrein-sensitive glycoprotein) (ITIH4), mRNA [NM_002218]
A_24_P350196	A_24_P350196	-0.82086	2.52E-17	3.69E-14	-16.0639	Down	Unknown
A_24_P350200	AK057071	-0.81746	3.21E-17	4.53E-14	-15.934	Down	Homo sapiens cDNA FLJ32509 fis, clone SMINT1000054. [AK057071]
A_23_P360209	ND3	-0.7006	4.22E-17	5.77E-14	-15.7875	Down	NADH-ubiquinone oxidoreductase chain 3 (EC 1.6.5.3) (NADH dehydrogenase subunit 3). [Source:Uniprot/SWISSPROT;Acc:P03897] [ENST00000361227]
A_24_P291658	ADH1A	-0.66103	4.46E-17	5.87E-14	-15.7584	Down	Homo sapiens alcohol dehydrogenase 1A (class I), alpha polypeptide (ADH1A), mRNA [NM_000667]
A_23_P148088	FGG	-0.74574	4.58E-17	5.87E-14	-15.7442	Down	Homo sapiens fibrinogen gamma chain (FGG), transcript variant gamma-A, mRNA [NM_000509]

A_23_P45945	EIF1	-0.45504	5.48E-17	6.81E-14	-15.6493	Down	Homo sapiens eukaryotic translation initiation factor 1 (EIF1), mRNA [NM_005801]
A_23_P402751	BC000228	-0.86772	7.59E-17	9.16E-14	-15.4777	Down	Homo sapiens cDNA clone IMAGE:3353308, **** WARNING: chimeric clone ****. [BC000228]
A_23_P331028	THRAP2	-0.89446	1.01E-16	1.19E-13	-15.3272	Down	Homo sapiens cDNA: FLJ21627 fis, clone COL08058. [AK025280]
A_23_P97541	C4BPA	-0.74847	1.66E-16	1.89E-13	-15.0719	Down	Homo sapiens complement component 4 binding protein, alpha (C4BPA), mRNA [NM_000715]
A_23_P38244	APOH	-0.65212	1.8E-16	2E-13	-15.0295	Down	Homo sapiens apolipoprotein H (beta-2-glycoprotein I) (APOH), mRNA [NM_000042]
A_23_P37445	RPLP1	-0.50429	2.28E-16	2.46E-13	-14.9098	Down	Homo sapiens ribosomal protein, large, P1 (RPLP1), transcript variant 1, mRNA [NM_001003]
A_23_P7342	UGT2B10	-0.52733	2.52E-16	2.65E-13	-14.859	Down	Homo sapiens UDP glucuronosyltransferase 2 family, polypeptide B10 (UGT2B10), mRNA [NM_001075]
A_24_P151464	SOD1	-0.39192	2.73E-16	2.79E-13	-14.8193	Down	Homo sapiens superoxide dismutase 1, soluble (amyotrophic lateral sclerosis 1 (adult)) (SOD1), mRNA [NM_000454]
A_23_P375372	FGA	-0.70662	4.62E-16	4.62E-13	-14.5536	Down	Homo sapiens fibrinogen alpha chain (FGA), transcript variant alpha, mRNA [NM_021871]
A_23_P95221	LPA	-0.49638	5.06E-16	4.94E-13	-14.5083	Down	Homo sapiens lipoprotein, Lp(a) (LPA), mRNA [NM_005577]
A_24_P179339	AY029066	-0.70587	6.87E-16	6.55E-13	-14.3568	Down	Homo sapiens Humanin (HN1) mRNA, complete cds. [AY029066]
A_23_P203183	APOC3	-0.55369	7.21E-16	6.69E-13	-14.3324	Down	Homo sapiens apolipoprotein C-III (APOC3), mRNA [NM_000040]
A_23_P164196	DLX4	-1.7117	7.34E-16	6.69E-13	-14.3234	Down	Homo sapiens distal-less homeobox 4 (DLX4), transcript variant 1, mRNA [NM_138281]
A_23_P79591	APOB	-0.44361	7.88E-16	7.02E-13	-14.2889	Down	Homo sapiens apolipoprotein B (including Ag(x) antigen) (APOB), mRNA [NM_000384]
A_23_P169494	ORM1	-0.7455	1.02E-15	8.88E-13	-14.1629	Down	Homo sapiens orosomucoid 1 (ORM1), mRNA [NM_000607]
A_23_P155509	AHSG	-0.58582	1.08E-15	9.21E-13	-14.1346	Down	Homo sapiens alpha-2-HS-glycoprotein (AHSG), mRNA [NM_001622]
A_24_P315986	FAM153C	-0.38728	1.32E-15	1.1E-12	-14.0365	Down	family with sequence similarity 153 member C
A_23_P13604	PEBP1	-0.44092	1.62E-15	1.33E-12	-13.9374	Down	Homo sapiens phosphatidylethanolamine binding protein 1 (PEBP1), mRNA [NM_002567]
A_23_P27215	UBB	-0.46198	1.75E-15	1.41E-12	-13.8977	Down	Homo sapiens ubiquitin B (UBB), mRNA [NM_018955]
A_24_P361896	MT2A	-0.70478	1.82E-15	1.43E-12	-13.8807	Down	Homo sapiens metallothionein 2A (MT2A), mRNA [NM_005953]
A_24_P264956	AK090417	-1.64103	2.14E-15	1.66E-12	-13.8012	Down	Homo sapiens mRNA for FLJ00325 protein. [AK090417]
A_23_P408249	PCK1	-0.76432	2.3E-15	1.75E-12	-13.7672	Down	Homo sapiens phosphoenolpyruvate carboxykinase 1 (soluble) (PCK1), mRNA [NM_002591]
A_24_P402415	SYT14	-0.71824	2.94E-15	2.19E-12	-13.6494	Down	Homo sapiens synaptotagmin XIV (SYT14), mRNA [NM_153262]
A_23_P106844	MT2A	-0.72074	3.35E-15	2.45E-12	-13.5874	Down	Homo sapiens metallothionein 2A (MT2A), mRNA [NM_005953]
A_23_P2492	C1S	-0.47378	3.86E-15	2.78E-12	-13.5196	Down	Homo sapiens complement component 1, s subcomponent (C1S), transcript variant 1, mRNA [NM_001734]
A_23_P110811	COX7C	-0.35469	4.48E-15	3.17E-12	-13.4491	Down	Homo sapiens cytochrome c oxidase subunit VIIc (COX7C), nuclear gene encoding mitochondrial protein, mRNA

							[NM_001867]
A_23_P36753	ALDH2	-0.65961	6.11E-15	4.25E-12	-13.3035	Down	Homo sapiens aldehyde dehydrogenase 2 family (mitochondrial) (ALDH2), nuclear gene encoding mitochondrial protein, mRNA [NM_000690]
A_24_P557534	CYTB	-0.81152	7.55E-15	5.16E-12	-13.2045	Down	Cytochrome b. [Source:Uniprot/SWISSPROT;Acc:P00156] [ENST00000361789]
A_23_P98002	CYP2E1	-0.6687	8.89E-15	5.98E-12	-13.1283	Down	Homo sapiens cytochrome P450, family 2, subfamily E, polypeptide 1 (CYP2E1), mRNA [NM_000773]
A_32_P15320	EEF1A1	-0.44562	9.14E-15	6.05E-12	-13.1154	Down	Homo sapiens eukaryotic translation elongation factor 1 alpha 1 (EEF1A1), mRNA [NM_001402]
A_23_P203191	APOA1	-0.51679	9.91E-15	6.45E-12	-13.0783	Down	Homo sapiens apolipoprotein A-I (APOA1), mRNA [NM_000039]
A_23_P75283	RBP4	-0.54887	1.3E-14	8.34E-12	-12.9517	Down	Homo sapiens retinol binding protein 4, plasma (RBP4), mRNA [NM_006744]
A_32_P190488	hCG_26523	-0.32399	1.39E-14	8.78E-12	-12.9214	Down	PREDICTED: Homo sapiens similar to 60S ribosomal protein L26 (LOC646161), mRNA [XR_018048]
A_23_P30098	ADH4	-0.73964	1.52E-14	9.46E-12	-12.8801	Down	Homo sapiens alcohol dehydrogenase 4 (class II), pi polypeptide (ADH4), mRNA [NM_000670]
A_23_P202053	ITIH2	-0.36794	1.55E-14	9.46E-12	-12.8731	Down	Homo sapiens inter-alpha (globulin) inhibitor H2 (ITIH2), mRNA [NM_002216]
A_23_P12767	CYP2C9	-0.58511	1.61E-14	9.68E-12	-12.8557	Down	Homo sapiens cytochrome P450, family 2, subfamily C, polypeptide 9 (CYP2C9), mRNA [NM_000771]
A_24_P213684	P4HB	-0.29396	2.11E-14	1.25E-11	-12.732	Down	Homo sapiens mRNA for OK/SW-CL.24, complete cds. [AB062434]
A_32_P47701	EEF1A1	-0.53612	2.16E-14	1.26E-11	-12.7209	Down	Homo sapiens eukaryotic translation elongation factor 1 alpha 1 (EEF1A1), mRNA [NM_001402]
A_23_P130333	TTR	-0.73805	2.92E-14	1.69E-11	-12.5837	Down	Homo sapiens transthyretin (prealbumin, amyloidosis type I) (TTR), mRNA [NM_000371]
A_23_P4649	APOC1	-0.59154	3.14E-14	1.79E-11	-12.5515	Down	Homo sapiens apolipoprotein C-I (APOC1), mRNA [NM_001645]
A_32_P342064	FTH1	-0.62374	3.71E-14	2.08E-11	-12.4756	Down	Homo sapiens ferritin, heavy polypeptide 1 (FTH1), mRNA [NM_002032]
A_23_P160354	AKT3	-0.89547	3.95E-14	2.19E-11	-12.4476	Down	Homo sapiens v-akt murine thymoma viral oncogene homolog 3 (protein kinase B, gamma) (AKT3), transcript variant 2, mRNA [NM_181690]
A_23_P303242	MT1X	-0.45468	4.14E-14	2.26E-11	-12.4268	Down	Homo sapiens metallothionein 1X (MT1X), mRNA [NM_005952]
A_24_P571937	LOC401717	-0.35739	5.08E-14	2.74E-11	-12.3356	Down	PREDICTED: Homo sapiens similar to eukaryotic translation elongation factor 1 alpha 2 (LOC401717), mRNA [XR_018189]
A_23_P77779	RPL19	-0.36516	5.45E-14	2.9E-11	-12.3036	Down	Homo sapiens ribosomal protein L19 (RPL19), mRNA [NM_000981]
A_24_P934592	CLEC18A	-1.27042	5.54E-14	2.91E-11	-12.2962	Down	C-type lectin domain family 18 member A
A_24_P388528	ST6GAL1	-0.34699	5.79E-14	3.01E-11	-12.2768	Down	Homo sapiens ST6 beta-galactosamide alpha-2,6-sialyltranferase 1 (ST6GAL1), transcript variant 1, mRNA [NM_173216]
A_23_P74943	KCNH1	-0.56721	5.94E-14	3.04E-11	-12.2658	Down	Homo sapiens potassium voltage-gated channel, subfamily H (eag-related), member 1 (KCNH1), transcript variant 1, mRNA [NM_172362]
A_23_P60933	MT1G	-0.59241	6.07E-14	3.08E-11	-12.2556	Down	Homo sapiens metallothionein 1G (MT1G), mRNA [NM_005950]

A_24_P50567	A_24_P50567	-0.47391	7.11E-14	3.56E-11	-12.1858	Down	Unknown
A_24_P149704	DAB2IP	-0.6399	1.01E-13	4.92E-11	-12.0326	Down	Homo sapiens DAB2 interacting protein (DAB2IP), transcript variant 2, mRNA [NM_138709]
A_23_P427703	MT1L	-0.46209	1.16E-13	5.58E-11	-11.9719	Down	H.sapiens mRNA for metallothionein isoform 1R. [X97261]
A_23_P9485	ORM2	-0.62264	1.27E-13	6.04E-11	-11.9322	Down	Homo sapiens orosomucoid 2 (ORM2), mRNA [NM_000608]
A_23_P337726	AK131288	-0.68538	1.34E-13	6.32E-11	-11.9071	Down	Homo sapiens cDNA FLJ16241 fis, clone HCASM2008536, weakly similar to DNA-REPAIR PROTEIN XRCC1. [AK131288]
A_23_P81158	ADH1C	-0.65412	1.48E-13	6.91E-11	-11.8634	Down	Homo sapiens alcohol dehydrogenase 1C (class I), gamma polypeptide (ADH1C), mRNA [NM_000669]
A_24_P916534	AF458587	-0.49473	1.56E-13	7.17E-11	-11.8423	Down	Homo sapiens hepatoma-derived growth factor HGDF5 mRNA, complete cds. [AF458587]
A_23_P125176	C15orf31	-1.14373	1.6E-13	7.31E-11	-11.8293	Down	Human pre-T/NK cell associated protein (6H9A) mRNA, complete cds. [L17330]
A_23_P414343	MT1H	-0.44887	2.19E-13	9.85E-11	-11.6954	Down	Homo sapiens metallothionein 1H (MT1H), mRNA [NM_005951]
A_23_P36901	CPB2	-0.34322	2.34E-13	1.04E-10	-11.6661	Down	Homo sapiens carboxypeptidase B2 (plasma) (CPB2), transcript variant 1, mRNA [NM_001872]
A_24_P334208	A_24_P334208	-0.43875	3.27E-13	1.44E-10	-11.5223	Down	Unknown
A_23_P62605	RPL11	-0.26817	4.27E-13	1.85E-10	-11.4095	Down	Homo sapiens ribosomal protein L11 (RPL11), mRNA [NM_000975]
A_23_P206760	HP	-0.59564	4.3E-13	1.85E-10	-11.4067	Down	Homo sapiens haptoglobin (HP), mRNA [NM_005143]
A_24_P237631	A_24_P237631	-0.7723	4.58E-13	1.96E-10	-11.3791	Down	Unknown
A_24_P681301	UBC	-0.50891	4.74E-13	2.01E-10	-11.3646	Down	Homo sapiens ubiquitin C (UBC), mRNA [NM_021009]
A_24_P610387	BE168511	-0.75249	5.02E-13	2.1E-10	-11.3405	Down	BE168511 QV3-HT0514-220300-127-d06 HT0514 Homo sapiens cDNA, mRNA sequence [BE168511]
A_32_P53049	EIF1	-0.39108	5.5E-13	2.28E-10	-11.3019	Down	Homo sapiens eukaryotic translation initiation factor 1 (EIF1), mRNA [NM_005801]
A_32_P212272	BE932552	-0.74309	5.59E-13	2.29E-10	-11.2955	Down	BE932552 QV3-HT0637-310800-319-h10 HT0637 Homo sapiens cDNA, mRNA sequence [BE932552]
A_24_P109214	APOC1	-0.47849	5.63E-13	2.29E-10	-11.292	Down	Homo sapiens apolipoprotein C-I (APOC1), mRNA [NM_001645]
A_23_P94879	F2	-0.3079	6.19E-13	2.49E-10	-11.2525	Down	Homo sapiens coagulation factor II (thrombin) (F2), mRNA [NM_000506]
A_24_P145787	A_24_P145787	-0.28304	6.44E-13	2.56E-10	-11.2359	Down	Unknown
A_23_P156687	CFB	-0.52358	6.68E-13	2.63E-10	-11.2205	Down	Homo sapiens complement factor B (CFB), mRNA [NM_001710]
A_23_P115261	AGT	-0.74884	6.8E-13	2.63E-10	-11.2131	Down	Homo sapiens angiotensinogen (serpin peptidase inhibitor, clade A, member 8) (AGT), mRNA [NM_000029]
A_23_P10500	ATP9B	-0.45278	6.8E-13	2.63E-10	-11.2131	Down	Homo sapiens ATPase, Class II, type 9B (ATP9B), mRNA [NM_198531]
A_23_P212500	TF	-0.51642	7.06E-13	2.71E-10	-11.1969	Down	Homo sapiens transferrin (TF), mRNA [NM_001063]
A_24_P889720	UBC	-0.3762	8.08E-13	3.07E-10	-11.1407	Down	Homo sapiens ubiquitin C (UBC), mRNA [NM_021009]

A_23_P95536	ADAM29	-0.94732	8.81E-13	3.32E-10	-11.1042	Down	Homo sapiens ADAM metallopeptidase domain 29 (ADAM29), mRNA [NM_014269]
A_24_P548354	RPS29	-0.3274	8.94E-13	3.33E-10	-11.0984	Down	Homo sapiens ribosomal protein S29 (RPS29), transcript variant 1, mRNA [NM_001032]
A_32_P111565	FTH1	-0.42789	9.28E-13	3.43E-10	-11.0828	Down	Homo sapiens ferritin, heavy polypeptide 1 (FTH1), mRNA [NM_002032]
A_23_P132611	VHL	-0.58149	1.25E-12	4.57E-10	-10.9597	Down	Homo sapiens full length insert cDNA clone ZD86C03. [AF088066]
A_23_P163782	MT1H	-0.61294	1.39E-12	5.05E-10	-10.9141	Down	Homo sapiens metallothionein 1H (MT1H), mRNA [NM_005951]
A_23_P6381	MN1	-0.72851	1.43E-12	5.12E-10	-10.9036	Down	Homo sapiens meningioma (disrupted in balanced translocation) 1 (MN1), mRNA [NM_002430]
A_23_P252413	MT2A	-0.67379	1.43E-12	5.12E-10	-10.9017	Down	Metallothionein-2 (MT-2) (Metallothionein-II) (MT-II) (Metallothionein-2A). [Source:Uniprot/SWISSPROT;Acc:P02795] [ENST00000245185]
A_23_P25030	HSD17B6	-0.42121	1.53E-12	5.42E-10	-10.8743	Down	Homo sapiens hydroxysteroid (17-beta) dehydrogenase 6 homolog (mouse) (HSD17B6), mRNA [NM_003725]
A_23_P18223	ITIH1	-0.33217	1.59E-12	5.59E-10	-10.8582	Down	Homo sapiens inter-alpha (globulin) inhibitor H1 (ITIH1), mRNA [NM_002215]
A_23_P428738	ANG	-0.55528	1.63E-12	5.66E-10	-10.8497	Down	Homo sapiens angiogenin, ribonuclease, RNase A family, 5 (ANG), mRNA [NM_001145]
A_23_P89649	KRTAP3-3	-0.73264	1.82E-12	6.24E-10	-10.803	Down	Homo sapiens keratin associated protein 3-3 (KRTAP3-3), mRNA [NM_033185]
A_32_P46722	BX647614	-0.54024	1.83E-12	6.24E-10	-10.8025	Down	BC004272 RAN protein {Homo sapiens} (exp=-1; wgp=0; cg=0), partial (98%) [THC2491566]
A_24_P800604	FAM83H	-0.66687	2.01E-12	6.82E-10	-10.7623	Down	Homo sapiens family with sequence similarity 83, member H (FAM83H), mRNA [NM_198488]
A_23_P252664	FUT6	-0.43955	2.08E-12	7E-10	-10.7485	Down	Human alpha (1,3) fucosyltransferase (FUT6) mRNA, major transcript I, complete cds. [U27333]
A_23_P136671	UGT2B7	-0.56483	2.6E-12	8.67E-10	-10.6577	Down	Homo sapiens UDP glucuronosyltransferase 2 family, polypeptide B7 (UGT2B7), mRNA [NM_001074]
A_23_P13244	OR8J1	-1.15488	2.83E-12	9.29E-10	-10.6227	Down	Homo sapiens olfactory receptor, family 8, subfamily J, member 1 (OR8J1), mRNA [NM_001005205]
A_23_P421493	HPR	-0.53293	2.91E-12	9.48E-10	-10.6111	Down	Homo sapiens haptoglobin-related protein (HPR), mRNA [NM_020995]
A_24_P126691	LOC645693	-0.39865	3.05E-12	9.83E-10	-10.5931	Down	PREDICTED: Homo sapiens similar to eukaryotic translation elongation factor 1 alpha 2 (LOC645693), mRNA [XR_017498]
A_32_P184796	RPLP0	-0.27524	3.09E-12	9.89E-10	-10.5878	Down	Homo sapiens ribosomal protein, large, P0 (RPLP0), transcript variant 2, mRNA [NM_053275]
A_32_P201292	BU587941	-0.92614	3.28E-12	1.04E-09	-10.5625	Down	AGENCOURT_8829785 NIH_MGC_141 Homo sapiens cDNA clone IMAGE:6386006 5', mRNA sequence [BU587941]
A_32_P110243	BC071734	-0.26554	3.43E-12	1.08E-09	-10.5445	Down	Homo sapiens cDNA clone MGC:88072 IMAGE:5549882, complete cds. [BC071734]
A_24_P306726	TPT1	-0.49335	3.48E-12	1.09E-09	-10.5393	Down	Homo sapiens tumor protein, translationally-controlled 1 (TPT1), mRNA [NM_003295]
A_23_P54840	MT1A	-0.56024	3.8E-12	1.18E-09	-10.5036	Down	Homo sapiens metallothionein 1A (MT1A), mRNA [NM_005946]
A_24_P299210	C1orf34	-0.71263	4.18E-12	1.28E-09	-10.465	Down	Homo sapiens mRNA for KIAA0452 protein, partial cds. [AB007921]

A_32_P429687	TRIM72	-0.97102	4.4E-12	1.33E-09	-10.4443	Down	Homo sapiens tripartite motif-containing 72 (TRIM72), mRNA [NM_001008274]
A_32_P47554	HINT1	-0.29079	4.41E-12	1.33E-09	-10.443	Down	Homo sapiens histidine triad nucleotide binding protein 1 (HINT1), mRNA [NM_005340]
A_24_P298939	EP400	-1.09646	4.78E-12	1.43E-09	-10.4107	Down	Homo sapiens E1A binding protein p400 (EP400), mRNA [NM_015409]
A_23_P36658	MGST1	-0.35471	4.84E-12	1.44E-09	-10.4061	Down	Homo sapiens microsomal glutathione S-transferase 1 (MGST1), transcript variant 1c, mRNA [NM_145791]
A_23_P333227	ERGIC1	-0.51499	4.97E-12	1.47E-09	-10.3948	Down	Homo sapiens endoplasmic reticulum-golgi intermediate compartment (ERGIC) 1 (ERGIC1), transcript variant 1, mRNA [NM_001031711]
A_24_P255252	A_24_P255252	-0.37915	5.01E-12	1.47E-09	-10.3916	Down	Unknown
A_23_P37441	B2M	-0.34912	5.09E-12	1.48E-09	-10.3857	Down	Homo sapiens beta-2-microglobulin (B2M), mRNA [NM_004048]
A_24_P336137	C22orf23	-0.95715	6.01E-12	1.73E-09	-10.3189	Down	Homo sapiens chromosome 22 open reading frame 23 (C22orf23), mRNA [NM_032561]
A_24_P148235	RPS27	-0.31021	6.13E-12	1.76E-09	-10.3106	Down	Homo sapiens ribosomal protein S27 (metallopanstimulin 1) (RPS27), mRNA [NM_001030]
A_23_P415021	METTL7A	-0.38556	6.2E-12	1.77E-09	-10.3062	Down	Homo sapiens methyltransferase like 7A (METTL7A), mRNA [NM_014033]
A_23_P129064	GATM	-0.70899	7.14E-12	2.02E-09	-10.2499	Down	Homo sapiens glycine amidinotransferase (L-arginine:glycine amidinotransferase) (GATM), nuclear gene encoding mitochondrial protein, mRNA [NM_001482]
A_32_P85676	STK32B	-0.7599	9.33E-12	2.62E-09	-10.1435	Down	Homo sapiens serine/threonine kinase 32B (STK32B), mRNA [NM_018401]
A_23_P212508	TF	-0.55902	1.05E-11	2.92E-09	-10.0972	Down	Homo sapiens transferrin (TF), mRNA [NM_001063]
A_24_P71373	SLC9A1	-0.44592	1.12E-11	3.11E-09	-10.0703	Down	Homo sapiens solute carrier family 9 (sodium/hydrogen exchanger), member 1 (antiporter, Na+/H+, amiloride sensitive) (SLC9A1), mRNA [NM_003047]
A_23_P32143	ALDOB	-0.59219	1.25E-11	3.42E-09	-10.0289	Down	Homo sapiens aldolase B, fructose-bisphosphate (ALDOB), mRNA [NM_000035]
A_24_P551842	CYTB	-0.67068	1.25E-11	3.42E-09	-10.027	Down	Cytochrome b. [Source:Uniprot/SWISSPROT;Acc:P00156] [ENST00000361789]
A_23_P416536	THC2539425	-0.85542	1.26E-11	3.43E-09	-10.0231	Down	XM_799236 mucin-associated surface protein (MASP) {Trypanosoma cruzi strain CL Brener} (exp=-1; wgp=0; cg=0), partial (6%) [THC2539425]
A_24_P314290	A_24_P314290	-0.92568	1.37E-11	3.71E-09	-9.99018	Down	Unknown
A_24_P318002	ZNF215	-1.10945	1.42E-11	3.8E-09	-9.97753	Down	Homo sapiens zinc finger protein 215 (ZNF215), mRNA [NM_013250]
A_23_P81926	PSORS1C2	-0.49434	1.46E-11	3.88E-09	-9.96757	Down	Homo sapiens psoriasis susceptibility 1 candidate 2 (PSORS1C2), mRNA [NM_014069]
A_23_P135084	RPL7A	-0.24394	1.5E-11	3.96E-09	-9.95673	Down	Homo sapiens ribosomal protein L7a (RPL7A), mRNA [NM_000972]
A_23_P395954	SSH2	-1.19834	1.51E-11	3.96E-09	-9.95438	Down	Homo sapiens mRNA for hSSH-2, complete cds. [AB072358]
A_23_P410115	ENTHD1	-0.82232	1.58E-11	4.13E-09	-9.93519	Down	Homo sapiens ENTH domain containing 1 (ENTHD1), mRNA [NM_152512]
A_23_P399921	SEC61A2	-0.46871	1.72E-11	4.46E-09	-9.90247	Down	Homo sapiens Sec61 alpha 2 subunit (S. cerevisiae), mRNA (cDNA clone MGC:32910 IMAGE:4823130), complete cds.

							[BC026179]
A_24_P927569	AW294929	-0.85346	1.84E-11	4.72E-09	-9.87526	Down	UI-H-BI2-ahr-f-04-0-UI.s1 NCI_CGAP_Sub4 Homo sapiens cDNA clone IMAGE:2727703 3', mRNA sequence [AW294929]
A_23_P203577	RPL27A	-0.25047	1.86E-11	4.74E-09	-9.87117	Down	Homo sapiens ribosomal protein L27a (RPL27A), mRNA [NM_000990]
A_32_P126214	OVCH1-AS1	-0.7215	2.04E-11	5.15E-09	-9.83607	Down	OVCH1 antisense RNA 1
A_32_P857658	RPLP1	-0.40839	2.32E-11	5.74E-09	-9.78555	Down	Homo sapiens ribosomal protein, large, P1 (RPLP1), transcript variant 1, mRNA [NM_001003]
A_23_P114740	CFHR1	-0.53797	2.32E-11	5.74E-09	-9.78455	Down	Homo sapiens complement factor H-related 1 (CFHR1), mRNA [NM_002113]
A_23_P111321	ARG1	-0.44227	2.45E-11	5.97E-09	-9.76433	Down	Homo sapiens arginase, liver (ARG1), mRNA [NM_000045]
A_24_P204334	A_24_P204334	-0.54249	2.45E-11	5.97E-09	-9.76423	Down	Unknown
A_24_P487296	N24703	-1.32523	2.51E-11	6.08E-09	-9.75499	Down	N24703 yx92a12.s1 Soares melanocyte 2NbHM Homo sapiens cDNA clone IMAGE:269182 3', mRNA sequence [N24703]
A_32_P155247	FTL	-0.29472	2.53E-11	6.1E-09	-9.75144	Down	Homo sapiens ferritin, light polypeptide (FTL), mRNA [NM_000146]
A_32_P144381	A_32_P144381	-0.53958	2.58E-11	6.18E-09	-9.74411	Down	Unknown
A_23_P38860	EXOC3L2	-0.43329	2.67E-11	6.38E-09	-9.72955	Down	protein 7 transactivated by hepatitis B virus X antigen [Source:RefSeq_peptide;Acc:NP_612635] [ENST00000252482]
A_32_P118731	STK32A	-0.75313	2.8E-11	6.63E-09	-9.71206	Down	serine/threonine kinase 32A
A_24_P266880	UBC	-0.35153	3.13E-11	7.36E-09	-9.66911	Down	Homo sapiens ubiquitin C (UBC), mRNA [NM_021009]
A_24_P522075	H97628	-1.12148	3.19E-11	7.47E-09	-9.66155	Down	H97628 yv93d03.s1 Soares melanocyte 2NbHM Homo sapiens cDNA clone IMAGE:250277 3', mRNA sequence [H97628]
A_23_P414793	CP	-0.42421	3.25E-11	7.56E-09	-9.65445	Down	Homo sapiens ceruloplasmin (ferroxidase) (CP), mRNA [NM_000096]
A_32_P198978	TMC1	-0.72815	4.29E-11	9.92E-09	-9.54713	Down	transmembrane channel like 1
A_24_P159648	BAIAP2	-0.34385	4.31E-11	9.92E-09	-9.54529	Down	Homo sapiens BAI1-associated protein 2 (BAIAP2), transcript variant 3, mRNA [NM_006340]
A_23_P7212	CFI	-0.30756	4.45E-11	1.02E-08	-9.53306	Down	Homo sapiens complement factor I (CFI), mRNA [NM_000204]
A_23_P32536	GNG2	-0.73403	4.63E-11	1.06E-08	-9.51701	Down	Homo sapiens guanine nucleotide binding protein (G protein), gamma 2 (GNG2), mRNA [NM_053064]
A_24_P937904	A_24_P937904	-1.17347	4.86E-11	1.1E-08	-9.49898	Down	Unknown
A_24_P929042	A_24_P929042	-0.55889	5.45E-11	1.23E-08	-9.45486	Down	Unknown
A_23_P2501	PAH	-0.4233	5.52E-11	1.24E-08	-9.44991	Down	Homo sapiens phenylalanine hydroxylase (PAH), mRNA [NM_000277]
A_23_P34018	RPL39	-0.24155	6.77E-11	1.51E-08	-9.37203	Down	Homo sapiens ribosomal protein L39 (RPL39), mRNA [NM_001000]
A_23_P151120	ACRBP	-0.3964	7.01E-11	1.55E-08	-9.35854	Down	Homo sapiens acrosin binding protein (ACRBP), mRNA [NM_032489]
A_23_P3054	LRRC9	-0.59551	8.19E-11	1.8E-08	-9.29935	Down	Leucine-rich repeat-containing protein 9. [Source:Uniprot/SPTREMBL;Acc:Q6ZRR7] [ENST00000254271]

A_23_P216812	CDKN2B	-0.58314	9.42E-11	2.05E-08	-9.24592	Down	Homo sapiens cyclin-dependent kinase inhibitor 2B (p15, inhibits CDK4) (CDKN2B), transcript variant 1, mRNA [NM_004936]
A_23_P18292	RPL14	-0.25636	9.46E-11	2.05E-08	-9.24451	Down	Homo sapiens ribosomal protein L14 (RPL14), transcript variant 2, mRNA [NM_003973]
A_32_P820503	FTH1	-0.42256	9.73E-11	2.1E-08	-9.23359	Down	Homo sapiens ferritin, heavy polypeptide 1 (FTH1), mRNA [NM_002032]
A_23_P94967	BX458358	-0.86778	1.13E-10	2.41E-08	-9.178	Down	BX458358 BX458358 Homo sapiens PLACENTA Homo sapiens cDNA clone CS0DE002YA02 3-PRIME, mRNA sequence [BX458358]
A_23_P110961	BRP44L	-0.33126	1.13E-10	2.41E-08	-9.17695	Down	Homo sapiens brain protein 44-like (BRP44L), mRNA [NM_016098]
A_23_P205348	TCL6	-1.13221	1.28E-10	2.72E-08	-9.12953	Down	Homo sapiens T-cell leukemia/lymphoma 6 (TCL6), transcript variant TCL6b1, mRNA [NM_020552]
A_23_P58359	ADH1A	-0.56857	1.3E-10	2.74E-08	-9.12482	Down	Homo sapiens alcohol dehydrogenase 1A (class I), alpha polypeptide (ADH1A), mRNA [NM_000667]
A_23_P83098	ALDH1A1	-0.32254	1.38E-10	2.9E-08	-9.10191	Down	Homo sapiens aldehyde dehydrogenase 1 family, member A1 (ALDH1A1), mRNA [NM_000689]
A_24_P931201	AY358179	-0.86152	1.5E-10	3.14E-08	-9.07041	Down	Homo sapiens clone DNA147310 GSQS6193 (UNQ6193) mRNA, complete cds. [AY358179]
A_24_P51167	BC031957	-0.35309	1.73E-10	3.59E-08	-9.0175	Down	Homo sapiens cDNA clone IMAGE:4838390. [BC031957]
A_23_P110299	TACR3	-0.94534	1.76E-10	3.65E-08	-9.0101	Down	Homo sapiens tachykinin receptor 3 (TACR3), mRNA [NM_001059]
A_24_P37962	HK3	-0.54648	2.01E-10	4.11E-08	-8.9613	Down	Homo sapiens hexokinase 3 (white cell) (HK3), nuclear gene encoding mitochondrial protein, mRNA [NM_002115]
A_24_P303118	RPL34	-0.33614	2.6E-10	5.31E-08	-8.86391	Down	Homo sapiens ribosomal protein L34 (RPL34), transcript variant 2, mRNA [NM_033625]
A_23_P68942	RPL3	-0.28583	2.67E-10	5.42E-08	-8.85446	Down	Homo sapiens ribosomal protein L3 (RPL3), transcript variant 1, mRNA [NM_000967]
A_24_P111547	MDH1B	-0.42097	2.93E-10	5.92E-08	-8.82009	Down	Homo sapiens malate dehydrogenase 1B, NAD (soluble) (MDH1B), mRNA [NM_001039845]
A_24_P378202	ARL11	-0.69593	3.07E-10	6.18E-08	-8.80216	Down	Homo sapiens ADP-ribosylation factor-like 11 (ARL11), mRNA [NM_138450]
A_23_P24515	ACAT1	-0.28624	3.4E-10	6.8E-08	-8.76451	Down	Homo sapiens acetyl-Coenzyme A acetyltransferase 1 (acetoacetyl Coenzyme A thiolase) (ACAT1), nuclear gene encoding mitochondrial protein, mRNA [NM_000019]
A_24_P119745	FN1	-0.32521	3.47E-10	6.89E-08	-8.75727	Down	Homo sapiens fibronectin 1 (FN1), transcript variant 1, mRNA [NM_212482]
A_23_P49849	SEZ6	-0.51769	3.48E-10	6.89E-08	-8.75616	Down	Homo sapiens seizure related 6 homolog (mouse) (SEZ6), mRNA [NM_178860]
A_23_P425197	IL23R	-0.54776	4.48E-10	8.75E-08	-8.66248	Down	Homo sapiens interleukin 23 receptor (IL23R), mRNA [NM_144701]
A_23_P2258	CCDC62	-0.37266	4.63E-10	9.01E-08	-8.65018	Down	Homo sapiens coiled-coil domain containing 62 (CCDC62), transcript variant 1, mRNA [NM_032573]
A_23_P155514	AHSG	-0.48448	4.88E-10	9.45E-08	-8.63081	Down	Homo sapiens alpha-2-HS-glycoprotein (AHSG), mRNA [NM_001622]
A_23_P139600	RASAL1	-0.67568	5.31E-10	1.02E-07	-8.59988	Down	Homo sapiens RAS protein activator like 1 (GAP1 like) (RASAL1), mRNA [NM_004658]
A_23_P323196	MDS2	-0.59333	5.47E-10	1.05E-07	-8.58884	Down	myelodysplastic syndrome 2 translocation associated

A_23_P81581	BHMT	-0.44039	5.53E-10	1.05E-07	-8.58514	Down	Homo sapiens betaine-homocysteine methyltransferase (BHMT), mRNA [NM_001713]
A_23_P50498	FTL	-0.26748	6.3E-10	1.2E-07	-8.53754	Down	Homo sapiens ferritin, light polypeptide (FTL), mRNA [NM_000146]
A_24_P931768	AW405469	-0.51681	7.93E-10	1.49E-07	-8.45289	Down	UI-HF-BL0-adh-c-11-0-UI.r1 NIH_MGC_37 Homo sapiens cDNA clone IMAGE:3061628 5', mRNA sequence [AW405469]
A_23_P154840	SOD1	-0.36963	8.15E-10	1.52E-07	-8.44287	Down	Homo sapiens superoxide dismutase 1, soluble (amyotrophic lateral sclerosis 1 (adult)) (SOD1), mRNA [NM_000454]
A_24_P928467	A_24_P928467	-0.75957	8.17E-10	1.52E-07	-8.44218	Down	Unknown
A_23_P315252	AK097322	-0.52017	8.27E-10	1.53E-07	-8.43766	Down	Homo sapiens cDNA FLJ40003 fis, clone STOMA2003716. [AK097322]
A_24_P392230	LOC641784	-0.39049	8.84E-10	1.63E-07	-8.41335	Down	xr55h07.x1 NCI_CGAP_Ov26 Homo sapiens cDNA clone IMAGE:2764093 3' similar to gb:X69181 60S RIBOSOMAL PROTEIN L31 (HUMAN);, mRNA sequence [AW302767]
A_23_P169249	SLC2A6	-0.32596	9.1E-10	1.67E-07	-8.40302	Down	Homo sapiens solute carrier family 2 (facilitated glucose transporter), member 6 (SLC2A6), mRNA [NM_017585]
A_23_P127584	NNMT	-0.52515	9.75E-10	1.78E-07	-8.37773	Down	Homo sapiens nicotinamide N-methyltransferase (NNMT), mRNA [NM_006169]
A_32_P227496	A_32_P227496	-0.39683	1.03E-09	1.87E-07	-8.357	Down	Unknown
A_23_P37718	CNGB1	-0.44285	1.05E-09	1.9E-07	-8.34911	Down	Homo sapiens cyclic nucleotide gated channel beta 1 (CNGB1), mRNA [NM_001297]
A_23_P206724	MT1E	-0.51028	1.06E-09	1.9E-07	-8.34878	Down	Homo sapiens metallothionein 1E (MT1E), mRNA [NM_175617]
A_23_P87860	MYF5	-0.59988	1.12E-09	1.99E-07	-8.32771	Down	Homo sapiens myogenic factor 5 (MYF5), mRNA [NM_005593]
A_23_P385017	G6PC	-0.51411	1.21E-09	2.14E-07	-8.30068	Down	Homo sapiens glucose-6-phosphatase, catalytic subunit (G6PC), mRNA [NM_000151]
A_32_P93894	A_32_P93894	-0.46808	1.29E-09	2.27E-07	-8.27613	Down	Unknown
A_32_P229965	THC2597697	-0.3298	1.33E-09	2.34E-07	-8.26403	Down	AA662695 nv06h02.s1 NCI_CGAP_Pr22 Homo sapiens cDNA clone IMAGE:1219443 3', mRNA sequence [AA662695]
A_32_P199664	THC2660197	-0.371	1.44E-09	2.52E-07	-8.23546	Down	Unknown
A_24_P297480	RNF113B	-0.38884	1.62E-09	2.82E-07	-8.19307	Down	Homo sapiens ring finger protein 113B (RNF113B), mRNA [NM_178861]
A_23_P37983	MT1B	-0.47827	1.64E-09	2.83E-07	-8.18957	Down	Homo sapiens metallothionein 1B (MT1B), mRNA [NM_005947]
A_24_P185368	ZSCAN4	-0.57594	1.83E-09	3.14E-07	-8.14949	Down	Homo sapiens zinc finger and SCAN domain containing 4 (ZSCAN4), mRNA [NM_152677]
A_23_P154037	AOX1	-0.35715	1.97E-09	3.37E-07	-8.12288	Down	Homo sapiens aldehyde oxidase 1 (AOX1), mRNA [NM_001159]
A_23_P68773	CECR1	-0.50386	2.19E-09	3.7E-07	-8.08573	Down	Homo sapiens cat eye syndrome chromosome region, candidate 1 (CECR1), transcript variant 1, mRNA [NM_017424]
A_23_P7221	RPL34	-0.27796	2.2E-09	3.71E-07	-8.08357	Down	Homo sapiens ribosomal protein L34 (RPL34), transcript variant 2, mRNA [NM_033625]
A_23_P126057	SCP2	-0.3272	2.23E-09	3.76E-07	-8.07767	Down	Homo sapiens sterol carrier protein 2 (SCP2), transcript variant 1, mRNA [NM_002979]
A_32_P221832	CD630738	-0.39525	2.27E-09	3.8E-07	-8.07153	Down	CD630738 56066364H1 FLP Homo sapiens cDNA, mRNA sequence [CD630738]

A_24_P118512	LOC645207	-0.40856	2.52E-09	4.14E-07	-8.03405	Down	PREDICTED: Homo sapiens similar to Eukaryotic translation initiation factor 4E type 3 (eIF4E type 3) (eIF-4E type 3) (mRNA cap-binding protein type 3) (Eukaryotic translation initiation factor 4E-like 3) (Eukaryotic translation initiation factor 4E h...
A_24_P418126	MLLT1	-0.33156	2.6E-09	4.25E-07	-8.02283	Down	Homo sapiens myeloid/lymphoid or mixed-lineage leukemia (trithorax homolog, Drosophila); translocated to, 1 (MLLT1), mRNA [NM_005934]
A_32_P44316	EEF1A1	-0.2438	3.04E-09	4.93E-07	-7.96747	Down	Homo sapiens eukaryotic translation elongation factor 1 alpha 1 (EEF1A1), mRNA [NM_001402]
A_23_P102172	CPO	-0.71574	3.28E-09	5.29E-07	-7.94038	Down	Homo sapiens carboxypeptidase O (CPO), mRNA [NM_173077]
A_32_P72927	THC2665094	-0.41987	3.34E-09	5.37E-07	-7.93345	Down	Unknown
A_23_P116898	A2M	-0.38996	3.48E-09	5.58E-07	-7.91884	Down	Homo sapiens alpha-2-macroglobulin (A2M), mRNA [NM_000014]
A_23_P149398	AQP10	-0.34418	3.55E-09	5.66E-07	-7.91202	Down	Homo sapiens small intestine aquaporin mRNA, complete cds. [AF159174]
A_23_P2601	HSP90B1	-0.30799	3.77E-09	6E-07	-7.89015	Down	Homo sapiens heat shock protein 90kDa beta (Grp94), member 1 (HSP90B1), mRNA [NM_003299]
A_23_P434968	KLF17	-0.6195	3.86E-09	6.09E-07	-7.88245	Down	Homo sapiens Kruppel-like factor 17 (KLF17), mRNA [NM_173484]
A_23_P251765	BANP	-0.27613	3.86E-09	6.09E-07	-7.88201	Down	Homo sapiens BTG3 associated nuclear protein (BANP), transcript variant 1, mRNA [NM_017869]
A_23_P258525	MC3R	-0.36075	4.04E-09	6.35E-07	-7.86575	Down	Homo sapiens melanocortin 3 receptor (MC3R), mRNA [NM_019888]
A_24_P221778	DUTP8	-0.51419	4.11E-09	6.43E-07	-7.86004	Down	deoxyuridine triphosphatase pseudogene 8
A_24_P170275	NKAIN3-IT1	-0.78779	4.15E-09	6.46E-07	-7.85671	Down	NKAIN3 intronic transcript
A_23_P42282	C4B	-0.23153	4.37E-09	6.78E-07	-7.83803	Down	Homo sapiens complement component 4B (Childo blood group) (C4B), mRNA [NM_001002029]
A_23_P119936	REG3A	-0.87747	4.41E-09	6.82E-07	-7.8347	Down	Homo sapiens regenerating islet-derived 3 alpha (REG3A), transcript variant 2, mRNA [NM_138938]
A_23_P207783	TBC1D16	-0.33336	4.76E-09	7.33E-07	-7.8079	Down	Homo sapiens cDNA FLJ34604 fis, clone KIDNE2013413. [AK091923]
A_23_P50504	FTL	-0.26652	5.1E-09	7.81E-07	-7.78282	Down	Homo sapiens ferritin, light polypeptide (FTL), mRNA [NM_000146]
A_32_P23113	AA903523	-0.32458	5.59E-09	8.48E-07	-7.75079	Down	AA903523 ok50h02.s1 NCI_CGAP_Lei2 Homo sapiens cDNA clone IMAGE:1517427 3', mRNA sequence [AA903523]
A_24_P58337	FTH1	-0.31918	6.06E-09	9.16E-07	-7.72198	Down	Homo sapiens ferritin, heavy polypeptide 1 (FTH1), mRNA [NM_002032]
A_23_P98294	SLC29A2	-0.43579	6.08E-09	9.16E-07	-7.72083	Down	Homo sapiens solute carrier family 29 (nucleoside transporters), member 2 (SLC29A2), mRNA [NM_001532]
A_23_P52749	ADAMTS15	-0.56018	6.2E-09	9.3E-07	-7.71409	Down	Homo sapiens ADAM metallopeptidase with thrombospondin type 1 motif, 15 (ADAMTS15), mRNA [NM_139055]
A_32_P201434	AA719641	-0.62805	7.14E-09	1.06E-06	-7.66394	Down	AA719641 zg52f01.s1 Soares_pineal_gland_N3HPG Homo sapiens cDNA clone IMAGE:396985 3', mRNA sequence [AA719641]
A_32_P1076	A_32_P1076	-0.54956	7.52E-09	1.11E-06	-7.6455	Down	Unknown

A_24_P217628	PRDM9	-0.32911	7.52E-09	1.11E-06	-7.64544	Down	Homo sapiens PR domain containing 9 (PRDM9), mRNA [NM_020227]
A_24_P125894	PPM1F	-0.47995	7.67E-09	1.13E-06	-7.63875	Down	Homo sapiens protein phosphatase 1F (PP2C domain containing) (PPM1F), mRNA [NM_014634]
A_23_P348911	LINC01126	-0.30925	8.12E-09	1.19E-06	-7.6183	Down	long intergenic non-protein coding RNA 1126
A_24_P394940	CYP2E1	-0.40208	8.89E-09	1.3E-06	-7.58665	Down	Homo sapiens cytochrome P450, family 2, subfamily E, polypeptide 1 (CYP2E1), mRNA [NM_000773]
A_23_P360114	A_23_P360114	-0.39475	9.33E-09	1.36E-06	-7.56946	Down	Unknown
A_24_P915591	COL4A6	-0.6999	9.4E-09	1.36E-06	-7.56683	Down	Homo sapiens collagen, type IV, alpha 6, mRNA (cDNA clone IMAGE:3951220), complete cds. [BC005305]
A_23_P134714	HRSP12	-0.32485	9.85E-09	1.42E-06	-7.55048	Down	Homo sapiens heat-responsive protein 12 (HRSP12), mRNA [NM_005836]
A_23_P128868	OR11H12	-0.47193	1.01E-08	1.46E-06	-7.54117	Down	Homo sapiens olfactory receptor, family 11, subfamily H, member 12 (OR11H12), mRNA [NM_001013354]
A_24_P300394	GSTA2	-0.36899	1.09E-08	1.56E-06	-7.51386	Down	Homo sapiens glutathione S-transferase A2 (GSTA2), mRNA [NM_000846]
A_23_P116694	RPS26	-0.27465	1.23E-08	1.75E-06	-7.47243	Down	Homo sapiens ribosomal protein S26 (RPS26), mRNA [NM_001029]
A_23_P140207	PCK2	-0.2908	1.24E-08	1.75E-06	-7.46948	Down	Homo sapiens phosphoenolpyruvate carboxykinase 2 (mitochondrial) (PCK2), nuclear gene encoding mitochondrial protein, transcript variant 1, mRNA [NM_004563]
A_23_P81484	ENST00000327625	-0.60615	1.36E-08	1.9E-06	-7.4372	Down	Unknown
A_23_P42868	IGFBP1	-0.63093	1.43E-08	1.99E-06	-7.41972	Down	Homo sapiens insulin-like growth factor binding protein 1 (IGFBP1), transcript variant 1, mRNA [NM_000596]
A_23_P33384	CIITA	-0.55008	1.53E-08	2.11E-06	-7.39693	Down	Homo sapiens class II, major histocompatibility complex, transactivator (CIITA), mRNA [NM_000246]
A_23_P200874	CCDC21	-0.45601	1.62E-08	2.24E-06	-7.37586	Down	Homo sapiens coiled-coil domain containing 21 (CCDC21), mRNA [NM_022778]
A_23_P266	PIN1L	-0.3166	1.66E-08	2.28E-06	-7.36807	Down	Homo sapiens PIN1 peptidyl-prolyl cis/trans isomerase-like (PIN1L) mRNA, complete cds. [U82382]
A_24_P24848	PSORS1C1	-0.43378	1.69E-08	2.32E-06	-7.36113	Down	Homo sapiens psoriasis susceptibility 1 candidate 1 (PSORS1C1), mRNA [NM_014068]
A_24_P911171	A_24_P911171	-0.49847	1.89E-08	2.57E-06	-7.32188	Down	Unknown
A_23_P126613	AQP10	-0.43074	2.08E-08	2.81E-06	-7.289	Down	Homo sapiens aquaporin 10 (AQP10), mRNA [NM_080429]
A_24_P125096	MT1X	-0.4035	2.25E-08	3.02E-06	-7.26132	Down	Homo sapiens metallothionein 1X (MT1X), mRNA [NM_005952]
A_23_P89799	ACAA2	-0.38421	2.28E-08	3.05E-06	-7.25691	Down	Homo sapiens acetyl-Coenzyme A acyltransferase 2 (mitochondrial 3-oxoacyl-Coenzyme A thiolase) (ACAA2), nuclear gene encoding mitochondrial protein, mRNA [NM_006111]
A_24_P298409	HLA-C	-0.34649	2.39E-08	3.18E-06	-7.23993	Down	Homo sapiens major histocompatibility complex, class I, C (HLA-C), mRNA [NM_002117]
A_24_P208721	KLHL18	-0.38054	2.4E-08	3.18E-06	-7.23953	Down	Homo sapiens kelch-like 18 (Drosophila) (KLHL18), mRNA [NM_025010]
A_23_P112698	PNMA2	-0.44902	2.42E-08	3.2E-06	-7.23635	Down	Homo sapiens paraneoplastic antigen MA2 (PNMA2), mRNA [NM_007257]
A_24_P930469	A_24_P930469	-0.37564	2.46E-08	3.24E-06	-7.23058	Down	Unknown

A_24_P401491	C9orf18	-0.58298	2.47E-08	3.24E-06	-7.22925	Down	Homo sapiens chromosome 9 open reading frame 18 (C9orf18), mRNA [NM_198469]
A_23_P405287	SEMA3D	-0.91247	2.6E-08	3.4E-06	-7.21161	Down	Homo sapiens sema domain, immunoglobulin domain (Ig), short basic domain, secreted, (semaphorin) 3D (SEMA3D), mRNA [NM_152754]
A_23_P60599	UGT1A6	-0.31104	2.63E-08	3.43E-06	-7.20729	Down	Homo sapiens UDP glucuronosyltransferase 1 family, polypeptide A6 (UGT1A6), transcript variant 1, mRNA [NM_001072]
A_24_P930647	S75896	-0.49334	2.77E-08	3.59E-06	-7.18926	Down	clone 39-1 [human, brain, mRNA Mitochondrial Partial Mutant, 201 nt]. [S75896]
A_23_P4653	KLC3	-0.3304	2.87E-08	3.71E-06	-7.17675	Down	Homo sapiens kinesin light chain 3, mRNA (cDNA clone IMAGE:4310027), complete cds. [BC025318]
A_24_P313418	CCL22	-0.56598	2.94E-08	3.8E-06	-7.1681	Down	Homo sapiens chemokine (C-C motif) ligand 22 (CCL22), mRNA [NM_002990]
A_32_P74771	A_32_P74771	-0.42342	2.98E-08	3.83E-06	-7.16375	Down	Unknown
A_23_P146134	DUSP26	-0.39615	3.08E-08	3.95E-06	-7.15226	Down	Homo sapiens dual specificity phosphatase 26 (putative) (DUSP26), mRNA [NM_024025]
A_23_P150609	IGF2	-0.23276	3.35E-08	4.26E-06	-7.12353	Down	Homo sapiens insulin-like growth factor 2 (somatomedin A) (IGF2), transcript variant 2, mRNA [NM_001007139]
A_24_P134235	KHSRP	-0.34305	3.41E-08	4.33E-06	-7.11719	Down	Homo sapiens KH-type splicing regulatory protein (FUSE binding protein 2) (KHSRP), mRNA [NM_003685]
A_23_P203150	TMPRSS13	-0.26396	3.5E-08	4.43E-06	-7.10789	Down	Homo sapiens cDNA FLJ14892 fis, clone PLACE1004270, weakly similar to TRANSMEMBRANE PROTEASE, SERINE 2 (EC 3.4.21.-). [AK027798]
A_23_P305321	BC031867	-0.58307	3.71E-08	4.68E-06	-7.08831	Down	Homo sapiens cDNA clone IMAGE:4825132. [BC031867]
A_23_P1575	TRIM49	-0.41512	3.79E-08	4.76E-06	-7.08109	Down	Homo sapiens tripartite motif-containing 49 (TRIM49), mRNA [NM_020358]
A_24_P220947	AKR1C1	-0.24742	3.95E-08	4.95E-06	-7.06644	Down	Homo sapiens aldo-keto reductase family 1, member C1 (dihydrodiol dehydrogenase 1; 20-alpha (3-alpha)-hydroxysteroid dehydrogenase) (AKR1C1), mRNA [NM_001353]
A_24_P263384	CEP89	-0.3913	4.11E-08	5.13E-06	-7.05303	Down	centrosomal protein 89
A_24_P376224	AK098514	-0.26477	4.56E-08	5.68E-06	-7.0167	Down	Homo sapiens cDNA FLJ25648 fis, clone SYN01015. [AK098514]
A_23_P66241	MT1M	-0.47875	4.69E-08	5.83E-06	-7.00709	Down	Homo sapiens metallothionein 1M (MT1M), mRNA [NM_176870]
A_24_P933965	CN480368	-0.46251	4.93E-08	6.11E-06	-6.98966	Down	UI-H-EU0-azt-m-22-0-UI.s1 NCI_CGAP_Car1 Homo sapiens cDNA clone UI-H-EU0-azt-m-22-0-UI 3', mRNA sequence [CN480368]
A_32_P122240	ASCL5	-0.52402	5.98E-08	7.36E-06	-6.92336	Down	PREDICTED: Homo sapiens similar to Achaete-scute homolog 3 (bHLH transcriptional regulator Sgn-1) (Mash-3) (LOC647219), mRNA [XM_940966]
A_23_P29747	RPL23A	-0.32533	6.08E-08	7.45E-06	-6.91743	Down	Homo sapiens ribosomal protein L23a (RPL23A), mRNA [NM_000984]
A_24_P940738	A_24_P940738	-0.31451	6.65E-08	8.12E-06	-6.88667	Down	Unknown
A_23_P353667	C19orf30	-0.32998	6.77E-08	8.24E-06	-6.88044	Down	Homo sapiens chromosome 19 open reading frame 30 (C19orf30), mRNA [NM_174947]

A_23_P127627	DGKZ	-0.39607	7.02E-08	8.49E-06	-6.86827	Down	Homo sapiens diacylglycerol kinase, zeta 104kDa (DGKZ), transcript variant 2, mRNA [NM_003646]
A_23_P200203	ECHDC2	-0.32363	7.68E-08	9.24E-06	-6.83732	Down	Homo sapiens enoyl Coenzyme A hydratase domain containing 2 (ECHDC2), mRNA [NM_018281]
A_24_P57426	COL18A1	-0.2291	8.15E-08	9.74E-06	-6.81717	Down	Homo sapiens collagen, type XVIII, alpha 1 (COL18A1), transcript variant 1, mRNA [NM_030582]
A_23_P136125	FGB	-0.38498	8.23E-08	9.81E-06	-6.81361	Down	Homo sapiens fibrinogen beta chain (FGB), mRNA [NM_005141]
A_24_P83462	C9orf47	-0.26586	8.87E-08	1.05E-05	-6.78777	Down	Homo sapiens cDNA FLJ37523 fis, clone BRCAN2006401. [AK094842]
A_24_P170726	LOC645427	-0.52247	9.07E-08	1.07E-05	-6.78027	Down	Homo sapiens cDNA FLJ37088 fis, clone BRACE2017124. [AK094407]
A_24_P348594	UPK3A	-0.35892	1.13E-07	1.33E-05	-6.70459	Down	Homo sapiens uroplakin 3A (UPK3A), mRNA [NM_006953]
A_32_P175198	ACTG1	-0.32766	1.29E-07	1.5E-05	-6.65879	Down	Homo sapiens actin, gamma 1 (ACTG1), mRNA [NM_001614]
A_32_P228521	THC2715131	-0.32038	1.34E-07	1.55E-05	-6.64727	Down	Unknown
A_24_P344251	ITGAX	-0.47405	1.46E-07	1.67E-05	-6.61754	Down	Homo sapiens integrin, alpha X (complement component 3 receptor 4 subunit) (ITGAX), mRNA [NM_000887]
A_24_P389251	CYP3A4	-0.54	1.49E-07	1.69E-05	-6.61164	Down	Homo sapiens cytochrome P450, family 3, subfamily A, polypeptide 4 (CYP3A4), mRNA [NM_017460]
A_24_P564576	NLF2	-0.28662	1.7E-07	1.93E-05	-6.56468	Down	Homo sapiens nuclear localized factor 2 (NLF2), mRNA [NM_001007595]
A_32_P28309	AI916036	-0.31923	1.72E-07	1.94E-05	-6.5614	Down	AI916036 wi44c02.x1 NCI_CGAP_Co16 Homo sapiens cDNA clone IMAGE:2393090 3' similar to TR:O75300 O75300 ES/130. ;, mRNA sequence [AI916036]
A_32_P13728	HSPA8	-0.27695	1.95E-07	2.18E-05	-6.5184	Down	Homo sapiens heat shock 70kDa protein 8 (HSPA8), transcript variant 1, mRNA [NM_006597]
A_24_P282251	FGA	-0.34102	1.97E-07	2.18E-05	-6.51624	Down	Homo sapiens fibrinogen alpha chain (FGA), transcript variant alpha, mRNA [NM_021871]
A_24_P918575	THC2729414	-0.74493	2.42E-07	2.64E-05	-6.44512	Down	Unknown
A_24_P153490	EVI1	-0.51301	2.65E-07	2.88E-05	-6.41459	Down	Homo sapiens mRNA; cDNA DKFZp686J18113 (from clone DKFZp686J18113). [BX640908]
A_23_P951	CFHR2	-0.39873	2.68E-07	2.89E-05	-6.4111	Down	Homo sapiens complement factor H-related 2 (CFHR2), mRNA [NM_005666]
A_23_P123330	RPL30	-0.25568	2.85E-07	3.05E-05	-6.38972	Down	Homo sapiens ribosomal protein L30 (RPL30), mRNA [NM_000989]
A_24_P397204	AK054756	-0.62887	2.93E-07	3.13E-05	-6.38057	Down	Homo sapiens cDNA FLJ30194 fis, clone BRACE2001352. [AK054756]
A_24_P579482	AF334588	-0.52739	3.16E-07	3.37E-05	-6.35453	Down	Homo sapiens P25 mRNA, complete cds. [AF334588]
A_23_P130187	A_23_P130187	-0.33809	3.46E-07	3.66E-05	-6.32381	Down	Unknown
A_24_P268729	HEMK1	-0.2676	3.51E-07	3.69E-05	-6.31919	Down	Homo sapiens HemK methyltransferase family member 1 (HEMK1), mRNA [NM_016173]
A_24_P273180	LOC649179	-0.74162	4.38E-07	4.53E-05	-6.2445	Down	Homo sapiens cDNA FLJ16327 fis, clone SYNOV1000256, weakly similar to Human preferentially expressed antigen of melanoma (PRAME) mRNA. [AK131325]
A_23_P259357	SLC35E4	-0.28654	4.58E-07	4.71E-05	-6.22927	Down	Homo sapiens solute carrier family 35, member E4 (SLC35E4), mRNA [NM_001001479]

A_23_P123692	C9orf7	-0.26701	4.92E-07	5.03E-05	-6.20507	Down	Homo sapiens chromosome 9 open reading frame 7 (C9orf7), mRNA [NM_017586]
A_23_P34382	CLCA3	-0.37097	4.96E-07	5.05E-05	-6.20268	Down	Homo sapiens chloride channel, calcium activated, family member 3 (CLCA3), mRNA [NM_004921]
A_23_P46470	ERRFI1	-0.31045	5.09E-07	5.18E-05	-6.19366	Down	Homo sapiens ERBB receptor feedback inhibitor 1 (ERRFI1), mRNA [NM_018948]
A_32_P63729	BX117044	-0.26468	5.31E-07	5.36E-05	-6.17922	Down	BX117044 NCI_CGAP_Lu5 Homo sapiens cDNA clone IMAGp998D123852, mRNA sequence [BX117044]
A_24_P922440	A_24_P922440	-0.43617	6.49E-07	6.38E-05	-6.11163	Down	Unknown
A_23_P315318	DCAF8L2	-0.34946	6.74E-07	6.57E-05	-6.0987	Down	DDB1 and CUL4 associated factor 8 like 2
A_32_P144852	CR622110	-0.29785	7.31E-07	7.09E-05	-6.07125	Down	full-length cDNA clone CS0DC025YP03 of Neuroblastoma Cot 25-normalized of Homo sapiens (human) [CR622110]
A_23_P202034	THC2662526	-0.40056	8.54E-07	8.07E-05	-6.01894	Down	GUC2G_RAT (P55205) Guanylate cyclase 2G precursor (Guanylyl cyclase receptor G) (GC-G) (Kinase-like domain-containing soluble guanylyl cyclase) (ksGC) , partial (31%) [THC2662526]
A_24_P321581	SLC38A4	-0.27947	9.08E-07	8.56E-05	-5.99846	Down	Homo sapiens solute carrier family 38, member 4 (SLC38A4), mRNA [NM_018018]
A_32_P198177	PALM3	-0.35013	9.71E-07	9.07E-05	-5.97573	Down	paralemmin 3
A_23_P17814	PLA2G3	-0.24117	9.75E-07	9.09E-05	-5.97423	Down	Homo sapiens phospholipase A2, group III (PLA2G3), mRNA [NM_015715]
A_23_P311346	DENND2D	-0.22939	1.07E-06	9.77E-05	-5.94445	Down	Unknown
A_24_P479364	LINC01503	-0.25816	1.09E-06	9.96E-05	-5.93668	Down	long intergenic non-protein coding RNA 1503
A_23_P59950	SLC39A14	-0.23923	1.13E-06	0.000102	-5.9252	Down	Homo sapiens solute carrier family 39 (zinc transporter), member 14 (SLC39A14), mRNA [NM_015359]
A_24_P912396	A_24_P912396	-0.56288	1.2E-06	0.000108	-5.90467	Down	Unknown
A_23_P15174	MT1F	-0.3459	1.23E-06	0.000111	-5.89478	Down	Homo sapiens metallothionein 1F (MT1F), mRNA [NM_005949]
A_23_P26426	CPNE7	-0.23014	1.35E-06	0.00012	-5.86606	Down	Homo sapiens copine VII (CPNE7), transcript variant 2, mRNA [NM_014427]
A_24_P937062	A_24_P937062	-0.4845	1.39E-06	0.000123	-5.85428	Down	Unknown
A_23_P6822	ITIH3	-0.23667	1.88E-06	0.000161	-5.75424	Down	Homo sapiens inter-alpha (globulin) inhibitor H3 (ITIH3), mRNA [NM_002217]
A_24_P307498	ANKRD45	-0.35934	1.88E-06	0.000161	-5.75332	Down	Homo sapiens ankyrin repeat domain 45 (ANKRD45), mRNA [NM_198493]
A_24_P929289	A_24_P929289	-0.29099	1.9E-06	0.000161	-5.75062	Down	Unknown
A_24_P306994	A_24_P306994	-0.32819	1.9E-06	0.000161	-5.75053	Down	Unknown
A_24_P336510	CFHR1	-0.27527	2.19E-06	0.000183	-5.70198	Down	Homo sapiens complement factor H-related 1 (CFHR1), mRNA [NM_002113]
A_32_P97243	LINC01753	-0.3594	2.29E-06	0.00019	-5.68744	Down	long intergenic non-protein coding RNA 1753
A_23_P208302	APOC2	-0.35302	2.47E-06	0.000202	-5.66178	Down	Homo sapiens apolipoprotein C-II (APOC2), mRNA [NM_000483]
A_32_P166135	DB301332	-0.27552	2.52E-06	0.000205	-5.65479	Down	DB301332 BRAMY1 Homo sapiens cDNA clone BRAMY1000317 3', mRNA sequence [DB301332]

A_24_P937695	S81294	-0.23956	3.11E-06	0.000248	-5.58401	Down	DCC=deleted in colorectal cancer {alternatively spliced, exon 1A} [human, brain tumor, tumor no. 245, mRNA Partial, 216 nt]. [S81294]
A_23_P218717	UBOX5	-0.31023	3.13E-06	0.000249	-5.58229	Down	Homo sapiens U-box domain containing 5 (UBOX5), transcript variant 1, mRNA [NM_014948]
A_24_P641742	A_24_P641742	-0.42744	3.35E-06	0.000262	-5.55967	Down	Unknown
A_23_P29965	SMR3B	-0.24868	4.3E-06	0.000325	-5.47583	Down	Homo sapiens submaxillary gland androgen regulated protein 3 homolog B (mouse) (SMR3B), mRNA [NM_006685]
A_23_P130743	DKKL1	-0.42051	4.57E-06	0.00034	-5.45543	Down	Homo sapiens dickkopf-like 1 (soggy) (DKKL1), mRNA [NM_014419]
A_32_P390781	REXO1	-0.25972	4.57E-06	0.00034	-5.45511	Down	Homo sapiens REX1, RNA exonuclease 1 homolog (S. cerevisiae) (REXO1), mRNA [NM_020695]
A_23_P211079	OLIG2	-0.28006	4.82E-06	0.000356	-5.43756	Down	Homo sapiens oligodendrocyte lineage transcription factor 2 (OLIG2), mRNA [NM_005806]
A_24_P408715	RFT1	-0.36951	5.3E-06	0.000384	-5.40564	Down	RFT1 homolog
A_32_P88240	KLHDC6	-0.30728	5.7E-06	0.000408	-5.38082	Down	Homo sapiens kelch domain containing 6 (KLHDC6), mRNA [NM_207335]
A_32_P78623	LOC401098	-0.27744	5.75E-06	0.000409	-5.37838	Down	Homo sapiens hypothetical LOC401098, mRNA (cDNA clone IMAGE:4821395). [BC034803]
A_24_P374513	TMSB4XP4	-0.2563	6.07E-06	0.00043	-5.36004	Down	TMSB4X pseudogene 4
A_32_P74588	THC2612626	-0.30782	6.11E-06	0.000433	-5.3575	Down	Unknown
A_23_P155453	UCN2	-0.44245	6.7E-06	0.000467	-5.32653	Down	Homo sapiens urocortin 2 (UCN2), mRNA [NM_033199]
A_24_P152356	LOC127099	-0.24846	7.11E-06	0.000491	-5.30669	Down	PREDICTED: Homo sapiens similar to 60S acidic ribosomal protein P1 (LOC127099), mRNA [XM_060328]
A_23_P215549	PON3	-0.24478	7.16E-06	0.000493	-5.30448	Down	Homo sapiens paraoxonase 3 (PON3), mRNA [NM_000940]
A_23_P254688	TMEM108	-0.26219	7.38E-06	0.000505	-5.29442	Down	Homo sapiens transmembrane protein 108 (TMEM108), mRNA [NM_023943]
A_24_P42453	BC000845	-0.49083	7.46E-06	0.00051	-5.29079	Down	Homo sapiens cDNA clone IMAGE:3457769, partial cds. [BC000845]
A_24_P659090	BX093289	-0.32238	1.03E-05	0.000662	-5.18093	Down	BX093289 NCI_CGAP_Lu5 Homo sapiens cDNA clone IMAGp998C224070 ; IMAGE:1605069, mRNA sequence [BX093289]
A_23_P212968	UGT2B11	-0.27743	1.05E-05	0.00067	-5.17532	Down	Homo sapiens UDP glucuronosyltransferase 2 family, polypeptide B11 (UGT2B11), mRNA [NM_001073]
A_23_P319466	CYSLTR2	-0.23313	1.39E-05	0.000843	-5.08061	Down	Homo sapiens cysteinyl leukotriene receptor 2 (CYSLTR2), mRNA [NM_020377]
A_24_P926915	THC2510672	-0.23269	1.78E-05	0.001034	-4.99725	Down	Unknown
A_24_P16606	ZFP41	-0.28963	1.87E-05	0.001073	-4.98071	Down	Homo sapiens mRNA for FLJ00028 protein, partial cds. [AK024438]
A_24_P486924	A_24_P486924	-0.22973	2.32E-05	0.001287	-4.90874	Down	Unknown
A_24_P205074	AY033611	-0.79836	2.73E-05	0.001459	-4.85355	Down	Homo sapiens placenta immunoregulatory factor PLIF mRNA, complete cds. [AY033611]
A_24_P384239	A_24_P384239	-0.37799	2.88E-05	0.001516	-4.83543	Down	Unknown
A_24_P925901	A_24_P925901	-0.37649	2.91E-05	0.001528	-4.83212	Down	Unknown

A_32_P154139	A_32_P154139	-0.25324	3.06E-05	0.001595	-4.81562	Down	Unknown
A_23_P250164	HGD	-0.2472	3.25E-05	0.001675	-4.79517	Down	Homo sapiens homogentisate 1,2-dioxygenase (homogentisate oxidase) (HGD), mRNA [NM_000187]
A_24_P940375	A_24_P940375	-0.30239	3.27E-05	0.001684	-4.79238	Down	Unknown
A_24_P335092	SAA1	-0.64748	3.8E-05	0.001913	-4.74174	Down	Homo sapiens serum amyloid A1 (SAA1), transcript variant 1, mRNA [NM_000331]
A_23_P306203	SAA2	-0.72555	4.05E-05	0.002005	-4.72065	Down	Homo sapiens serum amyloid A2 (SAA2), mRNA [NM_030754]
A_23_P301925	COX1	-1.03224	4.08E-05	0.002015	-4.71793	Down	Cytochrome c oxidase subunit 1 (EC 1.9.3.1) (Cytochrome c oxidase polypeptide I). [Source:Uniprot/SWISSPROT;Acc:P00395] [ENST00000361624]
A_23_P307274	OR2H2	-0.26344	4.38E-05	0.002132	-4.6939	Down	Homo sapiens olfactory receptor, family 2, subfamily H, member 2 (OR2H2), mRNA [NM_007160]
A_24_P205184	LINC00907	-0.36215	4.5E-05	0.002182	-4.6848	Down	long intergenic non-protein coding RNA 907
A_23_P153827	MLLT1	-0.2994	5.43E-05	0.002523	-4.62061	Down	Homo sapiens myeloid/lymphoid or mixed-lineage leukemia (trithorax homolog, Drosophila); translocated to, 1 (MLLT1), mRNA [NM_005934]
A_23_P150979	SBEM	-0.31187	6.09E-05	0.002744	-4.58174	Down	Homo sapiens small breast epithelial mucin (SBEM), mRNA [NM_058173]
A_32_P102519	LOC541473	-0.36817	6.21E-05	0.002767	-4.57533	Down	Homo sapiens FKBP6-like (LOC541473), mRNA [NM_001013748]
A_24_P901778	BM926140	-0.25621	6.51E-05	0.002879	-4.55903	Down	AGENCOURT_6600679 NIH_MGC_114 Homo sapiens cDNA clone IMAGE:5764503 5', mRNA sequence [BM926140]
A_24_P94101	PRO2949	-0.24597	6.79E-05	0.002959	-4.54448	Down	Homo sapiens PRO2949 mRNA, complete cds. [AF119907]
A_23_P7194	LGI2	-0.29635	7.57E-05	0.003195	-4.50777	Down	Homo sapiens leucine-rich repeat LGI family, member 2 (LGI2), mRNA [NM_018176]
A_24_P750992	AL133564	-0.25083	7.59E-05	0.003198	-4.50672	Down	Homo sapiens mRNA; cDNA DKFZp434J186 (from clone DKFZp434J186). [AL133564]
A_32_P100379	PDGFRA	-0.31194	8.96E-05	0.003648	-4.44996	Down	AA599881 ag32e07.s1 Human bone marrow stromal cells Homo sapiens cDNA clone IMAGE:1091268 3' similar to gb:M21574 ALPHA PLATELET-DERIVED GROWTH FACTOR RECEPTOR PRECURSOR (HUMAN);, mRNA sequence [AA599881]
A_24_P922624	A_24_P922624	-0.24708	9.07E-05	0.003681	-4.44592	Down	Unknown
A_23_P23996	MAT1A	-0.26395	9.83E-05	0.003923	-4.41836	Down	Homo sapiens methionine adenosyltransferase I, alpha (MAT1A), mRNA [NM_000429]
A_32_P50357	BU580882	-0.30443	0.000104	0.004111	-4.39863	Down	in33g06.y1 Human Fetal Pancreas 1B Homo sapiens cDNA 5', mRNA sequence [BU580882]
A_32_P115451	SPG7	-0.24654	0.000108	0.004212	-4.38744	Down	SPG7, paraplegin matrix AAA peptidase subunit
A_23_P20484	FGL1	-0.27998	0.000138	0.005037	-4.30092	Down	Homo sapiens fibrinogen-like 1 (FGL1), transcript variant 4, mRNA [NM_201553]
A_24_P852099	A_24_P852099	-0.72451	0.000157	0.005548	-4.2576	Down	Unknown
A_24_P273214	A_24_P273214	-0.24458	0.000164	0.005731	-4.24199	Down	Unknown
A_32_P160200	THC2699786	-0.25443	0.00019	0.006401	-4.19045	Down	Unknown

A_32_P151317	BI818647	-0.25856	0.000199	0.006609	-4.17597	Down	BI818647 603033232F1 NIH_MGC_115 Homo sapiens cDNA clone IMAGE:5174623 5', mRNA sequence [BI818647]
A_32_P62963	MGC102966	-0.25476	0.000219	0.00714	-4.14247	Down	Homo sapiens similar to Keratin, type I cytoskeletal 16 (Cytokeratin-16) (CK-16) (Keratin-16) (K16), mRNA (cDNA clone MGC:102966 IMAGE:4752428), complete cds. [BC110641]
A_24_P932875	UNQ6490	-0.24309	0.000229	0.007419	-4.12589	Down	Homo sapiens clone DNA147309 YPLR6490 (UNQ6490) mRNA, complete cds. [AY358209]
A_24_P228228	B4GALT6	-0.24036	0.000307	0.009144	-4.02447	Down	Homo sapiens UDP-Gal:betaGlcNAc beta 1,4-galactosyltransferase, polypeptide 6 (B4GALT6), mRNA [NM_004775]
A_23_P201521	A_23_P201521	-0.23647	0.000308	0.009164	-4.02268	Down	Unknown
A_23_P358714	KIAA1324	-0.73872	0.000326	0.009558	-4.00296	Down	Homo sapiens clone DNA59770 AEPG2426 (UNQ2426) mRNA, complete cds. [AY358366]
A_23_P104318	DDIT4	-0.26491	0.00033	0.00963	-3.9993	Down	Homo sapiens DNA-damage-inducible transcript 4 (DDIT4), mRNA [NM_019058]
A_23_P413456	CIRBP	-0.23072	0.00034	0.009838	-3.98907	Down	Homo sapiens cDNA FLJ46566 fis, clone THYMU3040829, moderately similar to Cold-inducible RNA-binding protein. [AK128423]
A_24_P914134	PRNP	-0.29239	0.000366	0.010329	-3.96268	Down	Homo sapiens prion protein (p27-30) (Creutzfeldt-Jakob disease, Gerstmann-Strausler-Scheinker syndrome, fatal familial insomnia) (PRNP), transcript variant 4, mRNA [NM_001080122]
A_32_P402924	LOC400794	-0.24429	0.000421	0.011333	-3.91373	Down	Homo sapiens hypothetical gene supported by BC030596, mRNA (cDNA clone IMAGE:4816496). [BC020945]
A_24_P257511	BHMT	-0.23028	0.000439	0.011729	-3.89838	Down	Homo sapiens betaine-homocysteine methyltransferase (BHMT), mRNA [NM_001713]
A_23_P52569	NUDT8	-0.24077	0.000458	0.012031	-3.88349	Down	Homo sapiens nudix (nucleoside diphosphate linked moiety X)-type motif 8 (NUDT8), mRNA [NM_181843]
A_23_P321501	DHRS2	-0.36996	0.000467	0.012197	-3.87685	Down	Homo sapiens dehydrogenase/reductase (SDR family) member 2 (DHRS2), transcript variant 1, mRNA [NM_182908]
A_24_P710024	AK097322	-0.33711	0.000494	0.012701	-3.85675	Down	Homo sapiens cDNA FLJ40003 fis, clone STOMA2003716. [AK097322]
A_23_P38235	ACE	-0.33957	0.000509	0.012978	-3.84623	Down	Homo sapiens angiotensin I converting enzyme (peptidyl-dipeptidase A) 1 (ACE), transcript variant 1, mRNA [NM_000789]
A_23_P44274	FGA	-0.29273	0.000517	0.013089	-3.8408	Down	Homo sapiens fibrinogen alpha chain (FGA), transcript variant alpha-E, mRNA [NM_000508]
A_24_P32178	ENST00000359589	-0.40749	0.000544	0.013609	-3.82259	Down	Unknown
A_32_P481631	LINC01144	-0.27413	0.000554	0.013757	-3.81642	Down	long intergenic non-protein coding RNA 1144
A_23_P58407	UGT2B15	-0.28859	0.000604	0.014502	-3.78583	Down	Homo sapiens UDP glucuronosyltransferase 2 family, polypeptide B15 (UGT2B15), mRNA [NM_001076]
A_24_P119685	OBSCN	-0.24039	0.000685	0.015752	-3.74106	Down	Homo sapiens obscurin, cytoskeletal calmodulin and titin-interacting RhoGEF (OBSCN), mRNA [NM_052843]
A_23_P320622	TTTY10	-0.30932	0.000695	0.015911	-3.73567	Down	Homo sapiens testis-specific transcript, Y-linked 10 (TTTY10) on chromosome Y [NR_001542]
A_24_P649507	THC2680667	-0.52334	0.000896	0.019091	-3.64453	Down	Q9BVX4_HUMAN (Q9BVX4) MGC5566 protein, partial (23%) [THC2680667]

A_24_P68183	C3orf50	-0.26276	0.000985	0.020333	-3.6105	Down	Homo sapiens cDNA clone IMAGE:4156795. [BC011266]
A_23_P135417	GSTA1	-0.22946	0.001034	0.021098	-3.593	Down	Homo sapiens glutathione S-transferase A1 (GSTA1), mRNA [NM_145740]
A_23_P131139	DIRC1	-0.40147	0.001182	0.023133	-3.54442	Down	Homo sapiens disrupted in renal carcinoma 1 (DIRC1), mRNA [NM_052952]
A_23_P430718	SPATA19	-0.23871	0.001191	0.023248	-3.54157	Down	Homo sapiens spermatogenesis associated 19 (SPATA19), mRNA [NM_174927]
A_23_P48570	DHRS2	-0.37802	0.001304	0.024631	-3.50861	Down	Homo sapiens dehydrogenase/reductase (SDR family) member 2 (DHRS2), transcript variant 1, mRNA [NM_182908]
A_24_P396662	GSTM4	-0.23422	0.001469	0.02666	-3.46516	Down	Homo sapiens glutathione S-transferase M4 (GSTM4), transcript variant 2, mRNA [NM_147148]
A_24_P252057	RBM24	-0.27051	0.001665	0.029057	-3.41906	Down	Homo sapiens RNA binding motif protein 24 (RBM24), mRNA [NM_153020]
A_32_P118896	A_32_P118896	-0.41145	0.001978	0.03263	-3.35547	Down	Unknown
A_24_P153002	A_24_P153002	-0.25879	0.002088	0.033722	-3.33546	Down	Unknown
A_24_P713185	THC2595309	-0.24185	0.002137	0.034149	-3.32683	Down	HLMITCSEQ Hylobates lar complete mitochondrial DNA sequence, partial (3%) [THC2595309]
A_23_P110764	MYOT	-0.27123	0.002195	0.034664	-3.31695	Down	Homo sapiens myotilin (MYOT), mRNA [NM_006790]
A_32_P122623	BC004387	-0.25387	0.002319	0.035878	-3.29642	Down	Homo sapiens cDNA clone IMAGE:3619551. [BC004387]
A_32_P71864	A_32_P71864	-0.22977	0.002507	0.037829	-3.26734	Down	Unknown
A_23_P134405	A_23_P134405	-0.47515	0.002814	0.040711	-3.22394	Down	Unknown
A_23_P124905	NPTX1	-0.26796	0.00334	0.045873	-3.15925	Down	Homo sapiens neuronal pentraxin I (NPTX1), mRNA [NM_002522]
A_23_P55716	BCAM	-0.25177	0.003493	0.047215	-3.14225	Down	Homo sapiens basal cell adhesion molecule (Lutheran blood group) (BCAM), transcript variant 1, mRNA [NM_005581]

Table 2 The enriched pathway terms of the up-regulated differentially expressed genes

BIOCYC							
**Pathway ID**	**Pathway Name**	**P-value**	**FDR B&H**	**FDR B&Y**	**Bonferroni**	**Gene Count**	**Gene**
907943	superpathway of steroid hormone biosynthesis	1.23E-02	1.90E-01	6.85E-01	2.47E-01	02	HSD17B3,HSD17B11
545345	ascorbate recycling (cytosolic)	2.86E-02	1.90E-01	6.85E-01	5.71E-01	01	GLRX
547502	glutamine degradation/glutamate biosynthesis	2.86E-02	1.90E-01	6.85E-01	5.71E-01	01	GLS
142159	methionine salvage	4.25E-02	2.13E-01	7.65E-01	8.50E-01	01	MTR

			**KEGG**				
83035	ABC transporters	4.46E-04	4.46E-04	8.82E-02	5.18E-01	05	ABCB4,ABCC2,ABCG5,ABCC5,ABCA6
1026136	Signaling pathways regulating pluripotency of stem cells	1.52E-02	1.52E-02	6.15E-01	1.00E+00	06	ID1,WNT10A,MYC,JARID2,AKT3,DUSP9
193146	Bile secretion	1.90E-02	1.90E-02	6.15E-01	1.00E+00	04	ABCB4,ABCC2,SLC51B,ABCG5
1404797	Platinum drug resistance	2.09E-02	2.09E-02	6.15E-01	1.00E+00	04	ABCC2,ATM,AKT3,GSTT2
82969	Selenocompound metabolism	2.43E-02	2.43E-02	6.15E-01	1.00E+00	02	MARS,MTR
83109	Endometrial cancer	3.50E-02	3.50E-02	6.15E-01	1.00E+00	03	MYC,AKT3,LEF1
83111	Prostate cancer	3.66E-02	3.66E-02	6.15E-01	1.00E+00	04	HSP90B1,CREB3L3,AKT3,LEF1
413393	Glutathione biosynthesis, glutamate => glutathione	4.25E-02	4.25E-02	6.15E-01	1.00E+00	01	GCLM
83117	Acute myeloid leukemia	4.45E-02	4.45E-02	6.15E-01	1.00E+00	03	MYC,AKT3,LEF1
			**Pathway Interaction Database**				
137959	BARD1 signaling events	7.41E-04	5.26E-02	2.55E-01	5.26E-02	04	FANCD2,UBE2D3,ATM,NPM1
137998	TCR signaling in naive CD4+ T cells	1.08E-02	3.83E-01	1.00E+00	7.65E-01	04	ITK,RASSF5,RASGRP1,CD3G
138055	TCR signaling in naive CD8+ T cells	3.15E-02	6.53E-01	1.00E+00	1.00E+00	03	RASSF5,RASGRP1,CD3G
138030	Canonical NF-kappaB pathway	4.27E-02	6.53E-01	1.00E+00	1.00E+00	02	UBE2D3,ATM
			**REACTOME**				
1269904	ABC-family proteins mediated transport	1.04E-03	6.38E-01	1.00E+00	6.65E-01	07	ABCB4,ABCC2,PSMA3,PSMC6,ABCG5,ABCC5,ABCA6
1383087	rRNA modification in the nucleus and cytosol	2.08E-03	6.38E-01	1.00E+00	1.00E+00	05	RPS14,NOP58,UTP14A,DDX52,KRR1
1269603	Binding of TCF/LEF:CTNNB1 to target gene promoters	4.12E-03	6.38E-01	1.00E+00	1.00E+00	02	MYC,LEF1

1269649	Gene Expression	4.35E-03	6.38E-01	1.00E+00	1.00E+00	40	MARS,RPS14,FANCD2,UBE2D3,CLNS1A,NRBF2,PPA1,ZNF430,ZNF708,NOP58,ZNF804B,ZCRB1,ATM,MYC,PSMA3,UTP14A,NARS,PSMC6,BCL6,LSM3,JARID2,HIST4H4,GLS,RBM8A,ZNF649,ZNF738,ZNF697,NPM1,AKT3,ZNF354A,ZNF493,NEDD4L,DDX52,ZNF736,ZNF101,KRR1,ZNF716,CITED4,DDX21,RPL5
1269090	Budding and maturation of HIV virion	5.33E-03	6.38E-01	1.00E+00	1.00E+00	03	CHMP4C,CHMP2B,NEDD4L
1269710	Cytosolic tRNAaminoacylation	5.96E-03	6.38E-01	1.00E+00	1.00E+00	03	MARS,PPA1,NARS
1269650	Generic Transcription Pathway	7.66E-03	6.59E-01	1.00E+00	1.00E+00	22	FANCD2,UBE2D3,NRBF2,ZNF430,ZNF708,ZNF804B,ATM,MYC,BCL6,GLS,ZNF649,ZNF738,ZNF697,NPM1,AKT3,ZNF354A,ZNF493,NEDD4L,ZNF736,ZNF101,ZNF716,CITED4
1269201	Immunoregulatory interactions between a Lymphoid and a non-Lymphoid cell	1.37E-02	6.59E-01	1.00E+00	1.00E+00	06	KLRK1,CLEC2D,LILRB1,CLEC2B,CD3G,CD96
1270293	Activation of DNA fragmentation factor	1.45E-02	6.59E-01	1.00E+00	1.00E+00	02	HMGB1,HIST1H1C
1269171	Adaptive Immune System	2.85E-02	6.59E-01	1.00E+00	1.00E+00	19	KCTD6,UBE2D3,IFI30,NPEPPS,ARF1,KLRK1,CLEC2D,TRIM37,PSMA3,PSMC6,ITGAV,LILRB1,ITK,CLEC2B,AKT3,NEDD4L,RASGRP1,CD3G,CD96
			**GenMAPP**				
MAP00271	Methionine metabolism	5.44E-03	5.98E-02	1.81E-01	5.98E-02	02	MARS,MTR

MAP00970	AminoacyltRNA biosynthesis	2.70E-02	1.44E-01	4.36E-01	2.98E-01	02	MARS,NARS
MAP00480	Glutathione metabolism	3.93E-02	1.44E-01	4.36E-01	4.33E-01	02	GCLM,GSTT2
			**MSigDB C2 BIOCARTA (v6.0)**				
M10401	Telomeres, Telomerase, Cellular Aging, and Immortality	2.70E-02	5.06E-01	1.00E+00	1.00E+00	02	MYC,TERF1
M1467	The Co-Stimulatory Signal During T-cell Activation	3.61E-02	5.06E-01	1.00E+00	1.00E+00	02	ITK,CD3G
M9703	Role of BRCA1, BRCA2 and ATR in Cancer Susceptibility	3.61E-02	5.06E-01	1.00E+00	1.00E+00	02	FANCD2,ATM
			**PantherDB**				
P02727	Androgen/estrogene/progesterone biosynthesis	1.21E-03	6.54E-02	2.99E-01	6.54E-02	02	HSD17B3,LIPA
P02753	Methionine biosynthesis	1.46E-02	2.91E-01	1.00E+00	7.88E-01	01	MTR
P04375	5HT3 type receptor mediated signaling pathway	2.16E-02	2.91E-01	1.00E+00	1.00E+00	02	SLC18A2,STX3
P04398	p53 pathway feedback loops 2	2.67E-02	2.91E-01	1.00E+00	1.00E+00	03	ATM,MYC,AKT3
P00007	Axon guidance mediated by semaphorins	2.99E-02	2.91E-01	1.00E+00	1.00E+00	02	SEMA3A,NRP1
P00029	Huntington disease	3.23E-02	2.91E-01	1.00E+00	1.00E+00	05	TNFAIP8,ARF1,AKT3,CAPN2,ARL4A
P02773	S-adenosylmethionine biosynthesis	4.25E-02	3.28E-01	1.00E+00	1.00E+00	01	MTR
			**Pathway Ontology**				
PW:0000381	multidrug resistance-associated protein mediated transport	2.00E-03	5.99E-02	2.39E-01	5.99E-02	02	ABCC2,ABCC5
PW:0000027	glutamate metabolic	1.45E-02	1.22E-01	4.89E-01	1.22E-01	02	GCLM,GLS
PW:0000382	multidrug resistance protein mediated	1.46E-02	1.22E-01	4.89E-01	1.22E-01	01	ABCB4

	transport						
PW:0000142	ubiquinone biosynthetic	2.86E-02	1.22E-01	4.89E-01	1.22E-01	01	PGGT1B
PW:0000192	N-Glycans biosynthetic	2.86E-02	1.22E-01	4.89E-01	1.22E-01	01	PGGT1B
PW:0000081	D-glutamine and D-glutamate metabolic	2.86E-02	1.22E-01	4.89E-01	1.22E-01	01	GLS
PW:0000399	remethylation of homocysteine metabolism - cobalamin dependent	2.86E-02	1.22E-01	4.89E-01	1.22E-01	01	MTR
PW:0000183	the proteolytic involving calcium-dependent proteases	4.25E-02	1.59E-01	6.37E-01	1.59E-01	01	CAPN2
**SMPDB**							
SMP00319	Lysosomal Acid Lipase Deficiency (Wolman Disease)	1.46E-02	3.18E-01	1.00E+00	7.30E-01	01	LIPA
SMP00356	17-Beta Hydroxysteroid Dehydrogenase III Deficiency	1.46E-02	3.18E-01	1.00E+00	7.30E-01	01	HSD17B3
SMP00033	Methionine Metabolism	1.91E-02	3.18E-01	1.00E+00	9.54E-01	02	MARS,MTR

Table 3 The enriched pathway terms of the down-regulated differentially expressed genes

**BIOCYC**							
**Pathway ID**	**Pathway Name**	**P-value**	**FDR B&H**	**FDR B&Y**	**Bonferroni**	**Gene Count**	**Gene**
142307	oxidative ethanol degradation III	2.30E-03	1.06E-01	4.66E-01	1.06E-01	2	ALDH2,CYP2E1
545295	retinoate biosynthesis I	6.23E-03	1.22E-01	5.37E-01	2.87E-01	2	ALDH1A1,RBP4
545354	noradrenaline and adrenaline degradation	7.93E-03	1.22E-01	5.37E-01	3.65E-01	2	ADH4,ALDH2
545316	bile acid biosynthesis, neutral pathway	1.41E-02	1.39E-01	6.12E-01	6.49E-01	2	SCP2,ACAA2
142421	fatty acid beta-oxidation	1.91E-02	1.39E-01	6.12E-01	8.77E-01	2	SCP2,ACAA2
782396	glycolysis	2.18E-02	1.39E-01	6.12E-01	1.00E+00	2	HK3,ALDOB
142451	lactate fermentation (reoxidation of cytosolic NADH)	3.06E-02	1.39E-01	6.12E-01	1.00E+00	1	LDHA

142372	methionine degradation	4.56E-02	1.39E-01	6.12E-01	1.00E+00	1	MAT1A
547500	phenylethylamine degradation I	4.56E-02	1.39E-01	6.12E-01	1.00E+00	1	ALDH2
142208	S-adenosyl-L-methionine biosynthesis	4.56E-02	1.39E-01	6.12E-01	1.00E+00	1	MAT1A
			**KEGG**				
83073	Complement and coagulation cascades	2.68E-15	6.35E-13	3.84E-12	6.35E-13	17	A2M,F2,SERPINA1,FGA,FGB,FGG,PLG,CPB2,CFI,SERPINC1,ITGAX,CFB,C1S,C3,C4B,C4BPA,KNG1
212237	Mineral absorption	1.20E-11	1.43E-09	8.63E-09	2.85E-09	12	MT1A,MT1B,MT1E,MT1F,MT1G,MT1H,MT1M,MT1X,MT2A,FTH1,FTL,TF
83032	Drug metabolism - cytochrome P450	4.09E-11	3.23E-09	1.95E-08	9.69E-09	13	ADH1A,ADH1C,ADH4,MGST1,UGT2B7,UGT2B10,UGT1A6,AOX1,UGT2B11,CYP2C9,CYP2E1,CYP3A4,GSTA2
83031	Metabolism of xenobiotics by cytochrome P450	8.54E-11	5.06E-09	3.06E-08	2.02E-08	13	ADH1A,ADH1C,ADH4,MGST1,UGT2B7,UGT2B10,UGT1A6,UGT2B11,CYP2C9,CYP2E1,CYP3A4,AKR1C1,GSTA2
83020	Retinol metabolism	2.53E-10	1.20E-08	7.25E-08	6.00E-08	12	ADH1A,ADH1C,ADH4,UGT2B7,UGT2B10,ALDH1A1,UGT1A6,AOX1,HSD17B6,UGT2B11,CYP2C9,CYP3A4
83036	Ribosome	1.82E-09	7.18E-08	4.34E-07	4.31E-07	16	RPL23A,RPL30,RPL27A,RPL31,RPL34,RPL39,RPLP0,RPLP1,RPS26,RPS27,RPS29,RPL14,RPL3,RPL7A,RPL11,RPL19
673221	Chemical carcinogenesis	4.14E-09	1.40E-07	8.48E-07	9.81E-07	12	ADH1A,ADH1C,ADH4,MGST1,UGT2B7,UGT2B10,UGT1A6,UGT2B11,CYP2C9,CYP2E

							1,CYP3A4,GSTA2
82926	Glycolysis / Gluconeogenesis	6.96E-08	2.06E-06	1.25E-05	1.65E-05	10	HK3,ADH1A,ADH1C,ADH4,ALDH2,ALDOB,G6PC,LDHA,PCK1,PCK2
82940	Steroid hormone biosynthesis	2.74E-06	7.22E-05	4.36E-04	6.50E-04	8	UGT2B7,UGT2B10,UGT1A6,HSD17B6,UGT2B11,CYP2E1,CYP3A4,AKR1C1
132956	Metabolic pathways	8.54E-06	2.02E-04	1.22E-03	2.02E-03	40	HGD,HK3,ACAT1,MAT1A,ADH1A,ADH1C,ADH4,UGT2B7,UGT2B10,SCP2,ACAA2,ALDH1A1,ALDH2,ALDOB,UGT1A6,PLA2G3,AOX1,COX7C,PON3,DGKZ,ST6GAL1,ARG1,MT-CO1,MT-CYB,HSD17B6,MT-ND3,FUT6,UGT2B11,G6PC,CYP2C9,CYP2E1,CYP3A4,GATM,BHMT,NNMT,ADAM29,LDHA,PAH,PCK1,PCK2
**Pathway Interaction Database**							
137911	FOXA2 and FOXA3 transcription factor networks	3.05E-07	1.65E-05	7.53E-05	1.65E-05	8	F2,TTR,ALB,ALDOB,APOA1,IGFBP1,G6PC,PCK1
137945	amb2 Integrin signaling	8.91E-05	2.41E-03	1.10E-02	4.81E-03	6	HP,PLG,FN1,APOB,KNG1,LPA
137979	FOXA1 transcription factor network	4.23E-03	7.61E-02	3.48E-01	2.28E-01	4	SERPINA1,APOB,SOD1,COL18A1
138064	Syndecan-4-mediated signaling events	1.18E-02	1.60E-01	7.30E-01	6.38E-01	3	F2,PLG,FN1
138045	HIF-1-alpha transcription factor network	1.79E-02	1.93E-01	8.83E-01	9.65E-01	4	CP,IGFBP1,LDHA,TF
137932	IL6-mediated signaling events	2.84E-02	2.55E-01	1.00E+00	1.00E+00	3	A2M,HSP90B1,FGG
137917	Angiopoietin receptor Tie2-mediated signaling	3.76E-02	2.90E-01	1.00E+00	1.00E+00	3	F2,PLG,FN1
**REACTOME**							
1339158	Metallothioneins bind metals	2.19E-15	7.83E-13	5.60E-12	1.57E-12	9	MT1A,MT1B,MT1E,MT1F,MT1G,

							MT1H,MT1M,MT1X,MT2A
1269367	Platelet degranulation	1.11E-14	2.64E-12	1.89E-11	7.92E-12	20	A2M,SERPINA1,FGA,FGB,AHSG,HRG,ALB,FGG,PLG,FN1,APOA1,APOH,IGF2,SOD1,ITIH3,ITIH4,KNG1,TF,ORM1,ORM2
1268691	Peptide chain elongation	4.79E-14	6.86E-12	4.90E-11	3.43E-11	17	RPL23A,RPL30,RPL27A,RPL31,RPL34,RPL39,RPLP0,RPLP1,RPS26,RPS27,RPS29,RPL14,EEF1A1,RPL3,RPL7A,RPL11,RPL19
1269350	Platelet activation, signaling and aggregation	9.44E-12	4.82E-10	3.45E-09	6.75E-09	24	A2M,GNG2,F2,SERPINA1,FGA,FGB,AHSG,HRG,ALB,FGG,PLG,FN1,DGKZ,APOA1,APOH,IGF2,SOD1,ITIH3,ITIH4,KNG1,AKT3,TF,ORM1,ORM2
1269897	Binding and Uptake of Ligands by Scavenger Receptors	2.94E-09	8.07E-08	5.77E-07	2.10E-06	10	HSP90B1,SAA1,HP,HPR,ALB,AMBP,APOA1,APOB,FTH1,FTL
1339147	Metabolism of fat-soluble vitamins	1.01E-07	2.41E-06	1.72E-05	7.22E-05	9	TTR,APOA1,APOA2,APOB,APOC2,APOC3,GC,AKR1C1,RBP4
1270005	Lipoprotein metabolism	1.84E-07	4.24E-06	3.03E-05	1.31E-04	10	A2M,ALB,APOA1,APOA2,APOB,APOC1,APOC2,APOC3,P4HB,LPA
1270189	Biological oxidations	4.96E-07	1.07E-05	7.68E-05	3.55E-04	16	MAT1A,ADH1A,ADH1C,ADH4,MGST1,UGT2B7,UGT2B10,ALDH1A1,ALDH2,UGT1A6,UGT2B11,CYP2C9,CYP2E1,CYP3A4,NNMT,GSTA2
1269340	Hemostasis	5.47E-07	1.15E-05	8.22E-05	3.91E-04	28	A2M,GNG2,F2,SERPINA1,FGA,FGB,AHSG,HRG,AL

							B,FGG,PLG,KLC3,FN1,DGKZ,APOA1,APOB,APOH,IGF2,SERPINC1,SOD1,ITGAX,ITIH3,ITIH4,KNG1,AKT3,TF,ORM1,ORM2
1269203	Innate Immune System	1.78E-05	2.70E-04	1.93E-03	1.27E-02	40	HSP90B1,HK3,HLA-C,PDGFRA,ACTG1,TTR,SAA1,SERPINA1,UBB,UBC,MGST1,HP,FGA,FGB,AHSG,FGG,BAIAP2,METTL7A,HSPA8,RASAL1,FN1,APOB,CFI,ARG1,FTH1,FTL,DAB2IP,B2M,ITGAX,CFB,C1S,C3,C4B,C4BPA,AKT3,EEF1A1,ORM1,ORM2,PEBP1,REG3A
**GenMAPP**							
MAP00071	MAP00071	1.52E-09	5.46E-08	2.28E-07	5.46E-08	10	ACAT1,ADH1A,ADH1C,ADH4,ACAA2,ALDH1A1,ALDH2,CYP2C9,CYP2E1,CYP3A4
MAP00010	MAP00010	8.49E-08	1.53E-06	6.38E-06	3.06E-06	9	HK3,ADH1A,ADH1C,ADH4,ALDH1A1,ALDH2,ALDOB,G6PC,LDHA
MAP00120	MAP00120	1.01E-06	1.21E-05	5.06E-05	3.63E-05	6	ADH1A,ADH1C,ADH4,ACAA2,ALDH1A1,ALDH2
MAP00380	MAP00380	5.98E-06	5.39E-05	2.25E-04	2.15E-04	7	ACAT1,ALDH1A1,ALDH2,AOX1,CYP2C9,CYP2E1,CYP3A4
MAP00350	MAP00350	8.62E-05	6.09E-04	2.54E-03	3.11E-03	5	HGD,ADH1A,ADH1C,ADH4,AOX1
MAP00620	MAP00620	1.02E-04	6.09E-04	2.54E-03	3.66E-03	5	ACAT1,ALDH1A1,ALDH2,LDHA,PCK1
MAP00640	MAP00640	2.19E-04	1.13E-03	4.70E-03	7.88E-03	4	ACAT1,ALDH1A1,ALDH2,LDHA

MAP00280	MAP00280	6.28E-04	2.83E-03	1.18E-02	2.26E-02	4	ACAA2,ALDH1A1,ALDH2,AOX1
MAP00561	MAP00561	7.51E-04	3.00E-03	1.25E-02	2.70E-02	5	ADH1A,ADH1C,ADH4,ALDH1A1,ALDH2
MAP00310	MAP00310	1.74E-03	6.28E-03	2.62E-02	6.28E-02	3	ACAT1,ALDH1A1,ALDH2
**MSigDB C2 BIOCARTA (v6.0)**							
M2842	Fibrinolysis Pathway	1.07E-08	4.44E-07	2.17E-06	7.77E-07	6	F2,FGA,FGB,FGG,PLG,CPB2
M15394	Acute Myocardial Infarction	1.22E-08	4.44E-07	2.17E-06	8.88E-07	7	F2,FGA,FGB,FGG,PLG,COL4A6,SERPINC1
M15997	Intrinsic Prothrombin Activation Pathway	3.70E-08	9.00E-07	4.39E-06	2.70E-06	7	F2,FGA,FGB,FGG,COL4A6,SERPINC1,KNG1
M3468	Genes encoding enzymes and their regulators involved in the remodeling of the extracellular matrix	8.31E-07	1.41E-05	6.86E-05	6.07E-05	16	A2M,F2,SERPINA1,AGT,HRG,PLG,AMBP,SERPINC1,ITIH1,ITIH2,ITIH3,ITIH4,ADAMTS15,KNG1,ADAM29,LPA
M4470	Extrinsic Prothrombin Activation Pathway	9.64E-07	1.41E-05	6.86E-05	7.04E-05	5	F2,FGA,FGB,FGG,SERPINC1
M917	Complement Pathway	1.77E-04	2.16E-03	1.05E-02	1.29E-02	4	CFB,C1S,C3,C4B
M5889	Ensemble of genes encoding extracellular matrix and extracellular matrix-associated proteins	4.98E-04	5.19E-03	2.53E-02	3.63E-02	30	A2M,CLEC18A,F2,SERPINA1,AGT,FGA,FGB,HRG,FGG,FGL1,PLG,CCL22,AMBP,COL4A6,FN1,IGF2,IGFBP1,SERPINC1,ITIH1,ITIH2,ITIH3,ITIH4,ADAMTS15,KNG1,ADAM29,COL18A1,SEMA3D,LGI2,LPA,REG3A
M6487	Platelet Amyloid Precursor Protein Pathway	1.16E-03	9.41E-03	4.58E-02	8.46E-02	3	F2,PLG,COL4A6
M7146	Classical Complement Pathway	1.16E-03	9.41E-03	4.58E-02	8.46E-02	3	C1S,C3,C4B
M13324	Hypoxia-Inducible Factor in the Cardiovascular System	1.43E-03	1.05E-02	5.10E-02	1.05E-01	3	VHL,LDHA,P4HB
**PantherDB**							
P00050	Plasminogen activating cascade	1.34E-07	3.90E-06	1.80E-05	7.49E-06	6	FGA,FGB,FGG,PLG,CPB2,LPA

P00011	Blood coagulation	1.39E-07	3.90E-06	1.80E-05	7.81E-06	8	F2,SERPINA1,FGA,FGB,FGG,PLG,SERPINC1,KNG1
P05914	Nicotine degradation	2.09E-03	3.91E-02	1.80E-01	1.17E-01	3	UGT2B7,UGT1A6,AOX1
P04372	5-Hydroxytryptamine degredation	3.08E-02	4.31E-01	1.00E+00	1.00E+00	2	ALDH1A1,ALDH2
P02773	S-adenosylmethionine biosynthesis	4.56E-02	5.10E-01	1.00E+00	1.00E+00	1	MAT1A
**Pathway Ontology**							
PW:0000484	altered lipoprotein metabolic	1.70E-08	7.51E-07	3.70E-06	1.31E-06	5	APOA1,APOB,APOC1,APOC2,APOC3
PW:0000482	lipoprotein metabolic	1.95E-08	7.51E-07	3.70E-06	1.50E-06	6	APOA1,APOA2,APOB,APOC1,APOC2,APOC3
PW:0000025	glycolysis/gluconeogenesis	4.40E-06	1.13E-04	5.56E-04	3.39E-04	6	HK3,ADH4,ALDH2,G6PC,LDHA,PCK1
PW:0000641	gluconeogenesis pathway	1.12E-04	2.15E-03	1.06E-02	8.58E-03	4	ALDOB,G6PC,PCK1,PCK2
PW:0000727	nateglinide pharmacokinetics pathway	7.03E-04	1.08E-02	5.33E-02	5.41E-02	2	CYP2C9,CYP3A4
PW:0000064	propanoate metabolic	9.21E-04	1.18E-02	5.83E-02	7.10E-02	3	ACAT1,ALDH2,LDHA
PW:0000071	valine, leucine and isoleucine degradation	2.09E-03	2.30E-02	1.14E-01	1.61E-01	3	ACAT1,ALDH2,AOX1
PW:0000058	fatty acid metabolic	5.09E-03	4.36E-02	2.15E-01	3.92E-01	3	ACAT1,ADH4,ALDH2
PW:0000054	tryptophan metabolic	5.09E-03	4.36E-02	2.15E-01	3.92E-01	3	ACAT1,ALDH2,AOX1
PW:0000474	coagulation cascade	7.93E-03	5.09E-02	2.51E-01	6.11E-01	2	F2,APOH
**SMPDB**							
SMP00272	Enoxaparin Pathway	5.13E-09	1.10E-07	5.55E-07	4.41E-07	7	F2,FGA,FGB,FGG,PLG,SERPINC1,KNG1
SMP00284	Urokinase Pathway	8.76E-08	5.80E-07	2.92E-06	7.54E-06	6	F2,FGA,FGB,FGG,PLG,KNG1
SMP00032	Valine, Leucine and Isoleucine Degradation	1.10E-03	1.10E-03	4.29E-03	2.16E-02	4	ACAT1,ACAA2,ALDH2,AOX1
SMP00196	Leigh Syndrome	2.48E-03	2.48E-03	8.55E-03	4.30E-02	3	ACAT1,ALDH2,PCK1
SMP00260	Clopidogrel Pathway	2.48E-03	2.48E-03	8.55E-03	4.30E-02	3	F2,CYP2C9,CYP3A4
SMP00060	Pyruvate Metabolism	2.48E-03	8.55E-03	4.30E-02	2.14E-01	3	ACAT1,ALDH2,PCK1
SMP00128	Gluconeogenesis	2.92E-03	9.65E-03	4.86E-02	2.51E-01	3	G6PC,PCK1,PCK2

SMP00123	Betaine Metabolism	4.72E-03	1.50E-02	7.58E-02	4.06E-01	2	MAT1A,BHMT
SMP00004	Glycine, Serine and Threonine Metabolism	6.47E-03	1.99E-02	1.00E-01	5.56E-01	3	ALDH2,GATM,BHMT
SMP00008	Phenylalanine and Tyrosine Metabolism	1.19E-02	3.52E-02	1.77E-01	1.00E+00	2	HGD,PAH

Table 4 The enriched GO terms of the up-regulated differentially expressed genes

GO ID	CATEGORY	GO Name	P Value	FDR B&H	FDR B&Y	Bonferroni	Gene Count	Gene
GO:0021879	BP	forebrain neuron differentiation	3.76E-05	1.09E-01	9.72E-01	1.58E-01	7	SEMA3A,HES1,PHLDA1,NRP1,SATB2,ROBO2,LEF1
GO:0002683	BP	negative regulation of immune system process	6.56E-05	1.09E-01	9.72E-01	2.75E-01	17	HMGB1,SERPINB9,MIF,HES1,TRIM38,KLRK1,ATM,PRNP,MYC,BCL6,LILRB1,CD84,PTPN2,HIST4H4,NFE2L2,MIXL1,CD96
GO:0021872	BP	forebrain generation of neurons	8.41E-05	1.09E-01	9.72E-01	3.53E-01	7	SEMA3A,HES1,PHLDA1,NRP1,SATB2,ROBO2,LEF1
GO:0051130	BP	positive regulation of cellular component organization	1.04E-04	1.09E-01	9.72E-01	4.36E-01	34	TPM1,TUB,ABCB4,S100A10,UBE2D3,MIF,BAMBI,HES1,NPEPPS,ID1,ARF1,SLC51B,ARHGEF10,ATM,FRMPD4,NAP1L2,PSMC6,BCL6,ITGAV,NRP1,JARID2,STX3,NFE2L2,GNL3,NPM1,NEDD4L,TERF1,ARPC3,KCTD17,CCT6A,ROBO2,LEF1,CAPRIN1,CPEB1
GO:1903707	BP	negative	1.42E-04	1.19E-01	1.00E+00	5.96E-01	9	HMGB1,HES1,

		regulation of hemopoiesis						MYC,BCL6,LILRB1,PTPN2,HIST4H4,NFE2L2,MIXL1
GO:1904753	BP	negative regulation of vascular associated smooth muscle cell migration	1.86E-04	1.21E-01	1.00E+00	7.82E-01	2	TPM1,NFE2L2
GO:0002715	BP	regulation of natural killer cell mediated immunity	2.81E-04	1.21E-01	1.00E+00	1.00E+00	5	SERPINB9,KLRK1,LILRB1,RASGRP1,CD96
GO:1901533	BP	negative regulation of hematopoietic progenitor cell differentiation	2.85E-04	1.21E-01	1.00E+00	1.00E+00	4	HES1,HIST4H4,NFE2L2,MIXL1
GO:0002682	BP	regulation of immune system process	3.15E-04	1.21E-01	1.00E+00	1.00E+00	37	HSP90B1,HMGB1,ABCB4,FANCD2,SERPINB9,UBE2D3,HPS1,MIF,HES1,TRIM38,MPP1,ARF1,KLRK1,IFNGR1,CLEC2D,ATM,PRNP,MYC,MYO10,PSMA3,HSPH1,PSMC6,BCL6,LILRB1,ITK,CD84,PTPN2,HIST4H4,NFE2L2,CLEC2B,ARPC3,RASGRP1,CD3G,MIXL1,IRAK4,LEF1,CD96
GO:0097694	BP	establishment of RNA localization to telomere	5.47E-04	1.21E-01	1.00E+00	1.00E+00	2	ATM,TERF1
GO:0031527	CC	filopodium membrane	2.02E-03	5.16E-01	1.00E+00	8.82E-01	3	UTRN,MYO10,ITGAV
GO:0005730	CC	nucleolus	2.37E-03	5.16E-01	1.00E+00	1.00E+00	23	MARS,RPS14,CHD2,FANCD2,NOL7,PHLDA1,CHD7,ESF1,NOP58,RBM34,MYC,MYO10,UTP14A

								,GNL3,NPM1,ZNF354A,DDX52,KRR1,TERF1,ARL4A,SCAF11,DDX21,RPL5
GO:0046581	CC	intercellular canaliculus	6.17E-03	5.23E-01	1.00E+00	1.00E+00	2	ABCB4,ABCC2
GO:0000815	CC	ESCRT III complex	9.26E-03	5.23E-01	1.00E+00	1.00E+00	2	CHMP4C,CHMP2B
GO:0032040	CC	small-subunit processome	1.08E-02	5.23E-01	1.00E+00	1.00E+00	3	NOP58,UTP14A,KRR1
GO:0043005	CC	neuron projection	1.14E-02	5.23E-01	1.00E+00	1.00E+00	26	HMGB1,SEMA3A,UTRN,ATCAY,MAGEE1,INPP5F,ARF1,IFNGR1,SLC18A2,CACYBP,MYC,FRMPD4,MYO10,NRP1,NELL2,STX3,KCNJ4,GLRX,KCNAB1,RBM8A,CAPN2,CASP4,ARPC3,ROBO2,CAPRIN1,CPEB1
GO:0030425	CC	dendrite	1.48E-02	5.23E-01	1.00E+00	1.00E+00	15	SEMA3A,ATCAY,MAGEE1,INPP5F,IFNGR1,FRMPD4,NELL2,STX3,KCNJ4,GLRX,KCNAB1,RBM8A,CAPN2,CAPRIN1,CPEB1
GO:0042581	CC	specific granule	1.49E-02	5.23E-01	1.00E+00	1.00E+00	2	STX3,STXBP3
GO:0016234	CC	inclusion body	1.80E-02	5.23E-01	1.00E+00	1.00E+00	4	DBF4B,BAG5,TRIM37,PSMC6
GO:0031045	CC	dense core granule	1.93E-02	5.23E-01	1.00E+00	1.00E+00	2	MYRIP,SLC18A2
GO:0008301	MF	DNA binding, bending	2.45E-03	2.81E-01	1.00E+00	1.00E+00	3	HMGB1,TERF1,LEF1
GO:0046983	MF	protein dimerization activity	4.34E-03	2.81E-01	1.00E+00	1.00E+00	30	STX2,S100A10,HPGD,CLNS1A,HPS1,HES1,CHMP4C,ID1,INPP5F,ARF1,SLC51B,KLRK1,ATM,CACYBP,TRIM37,MYC,ITGAV,LILRB1,HIST4H

								4,GCLM,NFE2L2,CREB3L3,ABCG5,NPM1,CAPN2,TERF1,RASGRP1,CD3G,MIXL1,PDCD10
GO:0043027	MF	cysteine-type endopeptidase inhibitor activity involved in apoptotic process	4.70E-03	2.81E-01	1.00E+00	1.00E+00	3	SERPINB9,TNFAIP8,LEF1
GO:0015405	MF	P-P-bond-hydrolysis-driven transmembrane transporter activity	6.01E-03	2.81E-01	1.00E+00	1.00E+00	6	TIMM17B,ABCB4,ABCC2,ABCG5,ABCC5,ABCA6
GO:0042623	MF	ATPase activity, coupled	6.34E-03	2.81E-01	1.00E+00	1.00E+00	11	LSG1,CHD2,ABCB4,ABCC2,MYO10, PSMC6,ABCG5, DDX52,ABCC5, ABCA6, DDX21
GO:0000774	MF	adenyl-nucleotide exchange factor activity	7.85E-03	2.81E-01	1.00E+00	1.00E+00	2	BAG5,HSPH1
GO:0004869	MF	cysteine-type endopeptidase inhibitor activity	8.22E-03	2.81E-01	1.00E+00	1.00E+00	4	SERPINB9,TNFAIP8,CST7,LEF1
GO:0003723	MF	RNA binding	9.36E-03	2.81E-01	1.00E+00	1.00E+00	34	HSP90B1,TRIP6, MARS,RPS14,HMGB1, CHD2,PPIG,CLNS1A,NOL7,ARF1,ESF1, FAM133B,NOP58,ZCRB1,RBM34, UTP14A,SSBP1, LSM3,HIST4H4, CCDC47,RBM8A,GNL3,NPM1, DDX52,KRR1, CCT6A,PABPC3,TRIM56,HIST1H1C,

								SCAF11,DDX21,CAPRIN1,RPL5,CPEB1
GO:0043225	MF	ATPase-coupled anion transmembrane transporter activity	9.50E-03	2.81E-01	1.00E+00	1.00E+00	2	ABCC2,ABCC5
GO:0016462	MF	pyrophosphatase activity	1.03E-02	2.81E-01	1.00E+00	1.00E+00	20	LSG1,CHD2,ABCB4,RAB33A,ABCC2,ATP5PD,CHD7,PPA1,ARF1,RASD1,MYO10,PSMC6,ABCG5,GNL3,DDX52,ABCC5,ARL4A,ABCA6,ENTPD6,DDX21

Biological Process(BP), Cellular Component(CC) and Molecular Functions (MF)

Table 5 The enriched GO terms of the down-regulated differentially expressed genes

GO ID	CATEGORY	GO Name	P Value	FDR B&H	FDR B&Y	Bonferroni	Gene Count	Gene
GO:0002576	BP	platelet degranulation	2.52E-18	9.72E-15	8.59E-14	9.72E-15	20	A2M,SERPINA1,FGA,FGB,AHSG,HRG,ALB,FGG,PLG,FN1,APOA1,APOH,IGF2,SOD1,ITIH3,ITIH4,KNG1,TF,ORM1,ORM2
GO:0010035	BP	response to inorganic substance	1.20E-14	2.33E-11	2.06E-10	4.65E-11	34	HK3,FABP1,HP,FGA,FGB,ALB,FGG,ALDOB,FN1,CP,APOB,ARG1,MT1A,MT1B,MT1E,MT1F,MT1G,MT1H,MT1M,MT1X,MT2A,MT-CYB,SOD1,CYB5A,CPNE7,CYP2E1,B2M,GATM,KCNH1,C3,LDHA,TF,COL18A1,REG

								3A
GO:0006614	BP	SRP-dependent cotranslational protein targeting to membrane	3.17E-14	4.08E-11	3.61E-10	1.22E-10	16	RPL23A,RPL30,RPL27A,RPL31,RPL34,RPL39,RPLP0,RPLP1,RPS26,RPS27,RPS29,RPL14,RPL3,RPL7A,RPL11,RPL19
GO:1990267	BP	response to transition metal nanoparticle	7.47E-14	6.59E-11	5.82E-10	2.89E-10	19	ALB,ALDOB,FN1,CP,ARG1,MT1A,MT1B,MT1E,MT1F,MT1G,MT1H,MT1M,MT1X,MT2A,MT-CYB,SOD1,B2M,GATM,TF
GO:0006953	BP	acute-phase response	3.10E-12	9.22E-10	8.15E-09	1.20E-08	12	F2,SAA1,SERPINA1,SAA2,HP,FGA,AHSG,FN1,ITIH4,ORM1,ORM2,REG3A
GO:0072376	BP	protein activation cascade	7.67E-12	1.98E-09	1.75E-08	2.96E-08	15	A2M,CFHR1,F2,FGA,FGB,FGG,APOH,CFI,SERPINC1,CFB,C1S,C3,C4B,C4BPA,KNG1
GO:1901575	BP	organic substance catabolic process	4.84E-11	1.17E-08	1.03E-07	1.87E-07	58	RPL23A,HGD,RPL30,RPL27A,RPL31,HSP90B1,RPL34,HINT1,RPL39,HK3,RPLP0,RPLP1,ACAT1,RPS26,RPS27,RPS29,FABP1,ADH1C,ADH4,UBB,UBC,SCP2,ACAA2,ALDH1A1,ALDH2,PLG,ALDOB,TRIM72,AMBP,VHL,PLA2G3,AOX1,PON3,APOA1,APOA2,APOB,APOC1,APOC2,APOC3,APOH,KHSRP,ARG1,SLC9A1,HSD17B6,FUT6,G6PC,DAB2IP,CYP3A4,BHMT,BANP,C4BPA,RPL14,LDHA,PAH,RPL3,RPL7A,RPL

								11,RPL19
GO:0045055	BP	regulated exocytosis	5.58E-11	1.27E-08	1.12E-07	2.16E-07	22	A2M,SERPINA1,FGA,FGB,AHSG,HRG,ALB,FGG,PLG,FN1,PLA2G3,APOA1,APOH,IGF2,SOD1,ITIH3,ITIH4,KNG1,TF,ORM1,ORM2,SYT14
GO:0006721	BP	terpenoid metabolic process	8.89E-11	1.91E-08	1.69E-07	3.43E-07	14	TTR,ADH1C,ADH4,ALDH1A1,APOA1,APOA2,APOB,APOC2,APOC3,CYP2C9,CYP2E1,CYP3A4,AKR1C1,RBP4
GO:0019752	BP	carboxylic acid metabolic process	5.34E-10	8.52E-08	7.53E-07	2.06E-06	38	HGD,HK3,ACAT1,MAT1A,FABP1,ADH1C,AGT,UGT2B7,UGT2B10,SCP2,ACAA2,ALDH1A1,ALDOB,UGT1A6,PLA2G3,PON3,APOA1,APOA2,ST6GAL1,APOC1,APOC2,APOC3,ARG1,UGT2B11,CYB5A,CYP2C9,CYP2E1,GATM,AKR1C1,BHMT,MDH1B,C3,LDHA,P4HB,PAH,ECHDC2,PCK1,PCK2
GO:0072562	CC	blood microparticle	8.65E-36	3.48E-33	2.29E-32	3.48E-33	35	A2M,CFHR1,TMPRSS13,ACTG1,F2,HP,HPR,AGT,FGA,FGB,AHSG,HRG,ALB,FGG,PLG,HSPA8,AMBP,FN1,CP,APOA1,APOA2,SERPINC1,GC,ITIH1,ITIH2,ITIH4,CFB,C1S,C3,C4B,C4BPA,KNG1,TF,ORM1,ORM2
GO:0005615	CC	extracellular space	4.42E-28	8.89E-26	5.84E-25	1.78E-25	75	A2M,CFHR1,TPT1,CLEC18A,RPL39,TMPRSS13,AC

								TG1,F2,TTR,UCN2,SAA1,SERPINA1,SAA2,UBB,UBC,HP,HPR,AGT,FGA,FGB,AHSG,HRG,ALB,FGG,FGL1,PLG,CCL22,HSPA8,AMBP,ANG,FN1,PLA2G3,PON3,CP,APOA1,APOA2,CPB2,APOB,APOC1,APOC2,APOC3,APOH,CFI,ARG1,IGF2,IGFBP1,SERPINC1,SSH2,DKKL1,SOD1,B2M,GC,ITIH1,ITIH2,ITIH4,CFB,SMR3B,CPO,ADAMTS15,C1S,C3,C4B,C4BPA,KNG1,RBP4,TF,EEF1A1,ORM1,COL18A1,ORM2,SEMA3D,PEBP1,LPA,REG3A,SEZ6
GO:0060205	CC	cytoplasmic vesicle lumen	1.07E-22	1.44E-20	9.44E-20	4.31E-20	23	A2M,HSP90B1,SAA1,SERPINA1,HP,FGA,FGB,AHSG,HRG,ALB,FGG,PLG,FN1,APOA1,APOB,APOH,IGF2,ITIH3,ITIH4,KNG1,TF,ORM1,ORM2
GO:0034774	CC	secretory granule lumen	4.89E-19	3.94E-17	2.59E-16	1.97E-16	19	A2M,SERPINA1,FGA,FGB,AHSG,HRG,ALB,FGG,PLG,FN1,APOA1,APOH,IGF2,ITIH3,ITIH4,KNG1,TF,ORM1,ORM2
GO:1990777	CC	lipoprotein particle	1.28E-12	5.32E-11	3.49E-10	5.13E-10	11	SAA1,SAA2,HPR,APOA1,APOA2,APOB,APOC1,APOC2,APOC3,APOH,LPA
GO:0022626	CC	cytosolic ribosome	1.40E-12	5.32E-11	3.49E-10	5.62E-10	16	RPL23A,RPL30,RPL27A,RPL31,RPL34,RPL39,RPLP

								0,RPLP1,RPS26,RPS27,RPS29,RPL14,RPL3,RPL7A,RPL11,RPL19
GO:0044433	CC	cytoplasmic vesicle part	1.44E-10	3.85E-09	2.53E-08	5.78E-08	30	A2M,HSP90B1,HLA-C,SAA1,SERPINA1,UBB,UBC,HP,FGA,FGB,AHSG,HRG,ALB,FGG,PLG,CNGB1,HSPA8,TRIM72,FN1,APOA1,APOB,APOH,IGF2,B2M,ITIH3,ITIH4,KNG1,TF,ORM1,ORM2
GO:0030141	CC	secretory granule	9.24E-10	2.06E-08	1.36E-07	3.72E-07	23	A2M,SERPINA1,FGA,FGB,AHSG,HRG,ALB,FGG,BAIAP2,PLG,FN1,APOA1,APOH,IGF2,SOD1,ACRBP,ITIH3,ITIH4,KNG1,TF,ORM1,ORM2,REG3A
GO:0005783	CC	endoplasmic reticulum	5.08E-05	6.81E-04	4.48E-03	2.04E-02	40	CLEC18A,HSP90B1,HLA-C,F2,SERPINA1,MGST1,HP,FGA,UGT2B7,UGT2B10,SCP2,UPK3A,ALB,ALDOB,METTL7A,VHL,COL4A6,UGT1A6,APOA1,APOA2,ST6GAL1,APOB,APOC1,SLC9A1,HSD17B6,UGT2B11,G6PC,CYB5A,CYP2C9,CYP2E1,CYP3A4,B2M,RFT1,ERGIC1,SEC61A2,COL18A1,P4HB,PEBP1,SLC39A14,SEZ6
GO:0008043	CC	intracellular ferritin complex	1.52E-04	1.91E-03	1.26E-02	6.11E-02	2	FTH1,FTL
GO:0061134	MF	peptidase regulator	3.17E-10	1.47E-07	1.08E-06	2.83E-07	18	A2M,SERPINA1,AGT,AHSG,HRG,

		activity						AMBP,FN1,SERPINC1,ACRBP,ITIH1,ITIH2,ITIH3,ITIH4,C3,C4B,KNG1,PEBP1,LPA
GO:0004866	MF	endopeptidase inhibitor activity	3.84E-10	1.47E-07	1.08E-06	3.43E-07	16	A2M,SERPINA1,AGT,AHSG,HRG,AMBP,SERPINC1,ITIH1,ITIH2,ITIH3,ITIH4,C3,C4B,KNG1,PEBP1,LPA
GO:0004857	MF	enzyme inhibitor activity	6.99E-10	1.47E-07	1.08E-06	6.24E-07	23	A2M,CDKN2B,SERPINA1,AGT,AHSG,HRG,AMBP,DGKZ,APOA1,APOA2,APOC1,APOC2,APOC3,SERPINC1,ITIH1,ITIH2,ITIH3,ITIH4,C3,C4B,KNG1,PEBP1,LPA
GO:0003735	MF	structural constituent of ribosome	1.16E-08	1.72E-06	1.27E-05	1.03E-05	16	RPL23A,RPL30,RPL27A,RPL31,RPL34,RPL39,RPLP0,RPLP1,RPS26,RPS27,RPS29,RPL14,RPL3,RPL7A,RPL11,RPL19
GO:0046914	MF	transition metal ion binding	1.55E-07	1.64E-05	1.21E-04	1.38E-04	42	RPS27,RPS29,ADH1A,ADH1C,ADH4,RNF113B,HRG,ALB,TRIM72,ANG,FN1,AOX1,CP,CPB2,UBOX5,ARG1,MT1A,MT1B,MT1E,MT1F,MT1G,MT1H,MT1M,MT1X,MT2A,MT-CO1,FTH1,FTL,SOD1,CYP2C9,SPG7,CYP2E1,CYP3A4,BHMT,CPO,ADAMTS15,KNG1,TRIM49,ADAM29,TF,PAH,PCK1
GO:0008289	MF	lipid binding	1.65E-07	1.64E-05	1.21E-04	1.48E-04	27	FABP1,ADH4,UGT2B7,SCP2,ALB,ALDH1A1,ALDOB,HSPA8,RASAL

								1,TRIM72,UGT1A6,APOA1,APOA2,APOB,APOC1,APOC2,APOC3,APOH,SLC9A1,DAB2IP,CYP3A4,GC,AKR1C1,C3,RBP4,PEBP1,SYT14
GO:0030234	MF	enzyme regulator activity	3.92E-07	3.50E-05	2.58E-04	3.50E-04	33	A2M,CDKN2B,RPLP1,TBC1D16,SERPINA1,AGT,AHSG,HRG,ALDH1A1,RASAL1,AMBP,FN1,DGKZ,APOA1,APOA2,APOC1,APOC2,APOC3,APOH,IGF2,SERPINC1,DAB2IP,ACRBP,ITIH1,ITIH2,ITIH3,ITIH4,C3,C4B,KNG1,PEBP1,LPA,ERRFI1
GO:0043178	MF	alcohol binding	1.26E-06	1.02E-04	7.52E-04	1.12E-03	10	ADH1C,ADH4,SCP2,ALDOB,APOA1,APOA2,APOC1,APOC3,GC,RBP4
GO:0016491	MF	oxidoreductase activity	6.34E-06	3.33E-04	2.46E-03	5.66E-03	25	HGD,ADH1A,ADH1C,ADH4,MGST1,ALDH1A1,ALDH2,AOX1,COX7C,CP,MT-CO1,MT-CYB,HSD17B6,MT-ND3,FTH1,SOD1,CYB5A,CYP2C9,CYP2E1,CYP3A4,AKR1C1,MDH1B,LDHA,P4HB,PAH
GO:0098772	MF	molecular function regulator	7.34E-05	2.18E-03	1.61E-02	6.55E-02	35	A2M,CDKN2B,RPLP1,PDGFRA,DENND2D,TBC1D16,SERPINA1,AGT,AHSG,HRG,ALDH1A1,RASAL1,AMBP,FN1,DGKZ,APOA1,APOA2,APOC1,APOC2,

		APOC3,APOH,IGF2,SERPINC1,DAB2IP,ACRBP,ITIH1,ITIH2,ITIH3,ITIH4,C3,C4B,KNG1,PEBP1,LPA,ERRFI1

Biological Process(BP), Cellular Component(CC) and Molecular Functions (MF)

Table 6 Topology table for up and down regulated genes

Regulation	Node	Degree	Betweenness	Stress	Closeness	Clustering Coefficient
Up	MYC	998	0.154309	168499346	0.375882	0.001122
Up	NPM1	820	0.117561	107362468	0.384137	0.004702
Up	UBE2D3	488	0.052712	19611516	0.354172	0.001309
Up	TERF1	429	0.069929	32105062	0.367951	0.002697
Up	PSMA3	403	0.05585	49381886	0.359233	0.003974
Up	RPL5	353	0.017877	15593168	0.360146	0.024758
Up	ATM	342	0.039464	14787526	0.356871	0.004204
Up	MDFI	324	0.055025	39487556	0.314011	0
Up	NEDD4L	321	0.045673	21988316	0.330131	6.53E-04
Up	RPS14	317	0.026808	17473646	0.364509	0.01837
Up	CCT6A	256	0.037281	17878614	0.365128	0.005999
Up	FANCD2	252	0.017648	6098152	0.358678	0.015464
Up	TRIP6	231	0.040921	23848338	0.333008	5.40E-04
Up	PSMC6	220	0.015559	10700136	0.343001	0.013025

Up	BCL6	207	0.02264	24871866	0.307037	0
Up	HSP90B1	200	0.029527	17863622	0.359166	0.007692
Up	NFE2L2	188	0.010348	3652870	0.324196	0.008433
Up	SCLT1	166	0.035705	13124124	0.328172	0.001634
Up	CLNS1A	161	0.015053	9139690	0.320598	0.00561
Up	RBM8A	158	0.015469	10955996	0.322952	7.19E-04
Up	CEP70	156	0.029342	19915780	0.319681	9.12E-04
Up	DDX21	151	0.01608	9050354	0.362055	0.021669
Up	HIST1H1C	149	0.010736	5288286	0.346977	0.012781
Up	RBM34	147	0.00977	5499606	0.331298	0.029493
Up	TPM1	147	0.017014	10998690	0.328357	0.002785
Up	NOP58	135	0.007483	7555478	0.323132	0.035872
Up	ARPC3	117	0.00987	13222134	0.307493	0
Up	SPOP	116	0.00679	2924770	0.307021	0.002899
Up	UTP14A	115	0.017201	7830734	0.339154	0.012219
Up	TRIM37	114	0.017605	9602260	0.319646	7.49E-04
Up	GNL3	111	0.003899	3216060	0.333716	0.061962
Up	KCTD17	109	0.013851	2865578	0.32324	0.003512
Up	HIST4H4	106	0.009751	5174142	0.332722	0.006162
Up	PDCD10	103	0.007998	7154936	0.296396	0.001948
Up	ARF1	103	0.014906	8505454	0.317531	0.00301
Up	STX3	103	0.010786	6295798	0.289064	0
Up	HMGB1	99	0.013111	13325200	0.310402	0
Up	CACYBP	99	0.008583	11077144	0.305857	0
Up	PTPN2	98	0.013814	7942688	0.318488	0.004544
Up	SSBP1	96	0.014867	7665946	0.338403	0.004151
Up	HSPH1	95	0.012083	5336120	0.351364	0.014232
Up	NDUFA8	95	0.012584	2649280	0.317843	0.00496
Up	LSM3	95	0.004744	1935918	0.307525	0.009752
Up	MARS	92	0.00844	4174480	0.350919	0.019182
Up	LEF1	85	0.005814	5392072	0.281374	0

Up	IBTK	84	0.006972	5407418	0.315461	0.002414
Up	DUSP9	81	0.012138	9069720	0.29673	0.002797
Up	KRR1	78	0.003862	2634586	0.320545	0.070344
Up	PFDN4	78	0.008664	3155248	0.30515	0.004372
Up	ITGAV	77	0.009984	12469680	0.300103	0
Up	ITGB3BP	76	0.006656	2680258	0.30499	0.001057
Up	S100A10	74	0.008338	1959770	0.319487	0.005797
Up	MIF	74	0.009065	3631472	0.333506	0.012274
Up	RASSF5	73	0.005664	3843624	0.288003	0
Up	CLEC2D	73	0.019109	14016710	0.261647	0
Up	UTRN	71	0.013585	3458972	0.351471	0.009936
Up	ZCRB1	71	0.006175	4067360	0.30319	0.010753
Up	NARS	70	0.009863	3662592	0.337342	0.007937
Up	PRNP	70	0.010724	2746068	0.311301	0.001815
Up	ATP5H	67	0.009326	8247346	0.30099	0
Up	IFT74	66	0.004592	2701986	0.297033	0.024314
Up	BAG5	66	0.006221	4710450	0.300881	0
Up	ID1	65	0.005023	2210798	0.296912	0
Up	NRP1	63	0.008454	6534574	0.289886	0
Up	IFT81	62	0.004817	2801802	0.299268	0.037805
Up	HES1	61	0.004189	3278606	0.296684	0
Up	IRAK4	60	0.003876	1320202	0.285609	0
Up	IFI30	59	0.008614	4094102	0.285581	0
Up	HEPACAM2	58	0.007573	6098564	0.26301	0
Up	PPIG	55	0.006258	2639128	0.293817	0.006969
Up	CAPN2	55	0.007653	8126808	0.295056	0
Up	COMMD6	54	0.0059	1234474	0.285356	0.015873
Up	ITK	52	0.004356	2606848	0.302212	0.001894
Up	ABCC2	52	0.006213	6458570	0.280748	0
Up	NPEPPS	51	0.006179	6986074	0.301287	0
Up	GLS	49	0.007375	1768552	0.317861	0.004646

Up	SATB2	49	0.008991	7379712	0.282387	0
Up	JARID2	47	0.001542	942940	0.283697	0
Up	MPP1	46	0.006755	4389882	0.300305	0
Up	DCUN1D2	46	4.97E-04	594444	0.289988	0
Up	CHD7	45	0.003555	5309116	0.27008	0
Up	NAPG	44	0.005217	2325312	0.299392	0.002299
Up	SCAF11	43	0.004241	5056338	0.291224	0
Up	PPA1	42	0.005151	1881814	0.322432	0.017422
Up	KCTD6	41	0.004265	2758776	0.289323	0.004598
Up	DDX52	41	0.003878	1210946	0.316924	0.022942
Up	CCDC47	41	0.004489	2995770	0.29896	0
Up	CAPRIN1	40	0.001837	2521356	0.313909	0
Up	NRBF2	38	0.0047	3710704	0.280721	0
Up	ARGLU1	37	0.00188	1921808	0.312591	0
Up	NELL2	36	0.004315	2944396	0.261071	0
Up	IFNGR1	35	0.00348	2746178	0.266939	0
Up	CASP4	34	0.005413	3373256	0.290699	0.001783
Up	DNAJC19	34	0.005049	3323192	0.282483	0
Up	SERPINB9	33	0.004549	1302470	0.317617	0.013258
Up	MTR	32	0.004364	3058286	0.299933	0.002151
Up	TRIM38	32	0.002248	952994	0.29896	0.006154
Up	CBWD3	31	0.004891	1781034	0.256278	0
Up	STXBP3	31	0.004689	3028350	0.292103	0.007246
Up	AKT3	31	0.003391	1317202	0.291545	0
Up	PPIL2	31	0.003098	1349508	0.300585	0
Up	CHMP2B	30	0.003637	2919774	0.285623	0
Up	NUCB2	30	0.003591	3543426	0.282469	0
Up	CHGB	29	0.004035	1659224	0.294742	0.007389
Up	EML4	28	0.002451	2095658	0.292147	0
Up	LYRM4	27	0.004342	1695094	0.263571	0
Up	GCC2	27	0.003608	2284202	0.275057	0

Up	RASL10B	26	0.004556	1200276	0.302212	0.003077
Up	PABPC3	25	0.002577	650314	0.321166	0.021739
Up	STX2	24	0.001342	695522	0.293817	0.019048
Up	GCLM	24	0.001871	1026924	0.290205	0
Up	GLRX	23	0.004024	914578	0.311618	0.021053
Up	ESF1	22	0.001736	844196	0.292765	0.051948
Up	TNFAIP8	22	0.002291	2402500	0.272668	0
Up	ARHGEF10	21	0.002026	1081974	0.260194	0
Up	TMEM92	21	0.001294	281894	0.277568	0.025641
Up	TIMM17B	21	0.001588	855894	0.281265	0
Up	FAM49B	21	0.00205	1156500	0.285468	0
Up	PHLDA1	20	0.004157	819116	0.311234	0
Up	CREB3L3	20	0.001653	1120442	0.283448	0
Up	CCDC91	20	0.001272	622256	0.266743	0
Up	CTNNAL1	20	0.00201	942218	0.270043	0
Up	LSG1	20	0.00122	1112348	0.282868	0
Up	NFIL3	19	0.001508	687668	0.248385	0
Up	MAGEE1	19	0.001079	597640	0.258917	0
Up	SYAP1	19	0.00135	1051836	0.282607	0
Up	KCNJ4	19	0.002415	1124880	0.237292	0
Up	TRIM56	19	0.001618	1090884	0.290582	0
Up	ROBO2	18	0.003296	751760	0.300725	0.013072
Up	CHMP4C	18	0.002677	1634820	0.264737	0
Up	MIS18BP1	18	0.001931	800300	0.272361	0
Up	DBF4B	17	7.17E-04	202448	0.289438	0
Up	NOL7	17	0.001439	927832	0.291107	0
Up	CD96	17	0.002211	1548868	0.254816	0
Up	ZNF101	16	0.002289	580804	0.271355	0
Up	UNC5CL	16	0.002528	1570328	0.267173	0
Up	ODF2L	16	0.001609	878332	0.280491	0
Up	TAOK1	16	0.001633	894288	0.285679	0

Up	TRIM7	15	5.10E-04	186870	0.283961	0.027778
Up	TBC1D3	15	4.06E-04	306790	0.26238	0
Up	CPEB1	15	0.002205	1151826	0.273129	0
Up	HSD17B3	15	0.001701	1650014	0.258445	0
Up	RASD1	14	0.00158	289796	0.268408	0
Up	KCNAB1	14	0.001211	323584	0.296457	0.036364
Up	ARL4A	14	0.002098	1161666	0.261118	0
Up	MYO10	14	5.11E-04	453582	0.285946	0
Up	HPS1	13	0.002106	1991020	0.279075	0.027778
Up	ACTL8	13	0.001273	311004	0.310634	0.025641
Up	CDC42SE1	13	0.001154	743944	0.277475	0
Up	ENTPD6	13	0.001598	861114	0.240994	0
Up	CHD2	13	5.55E-04	377898	0.265754	0
Up	SDF2	13	0.001255	751196	0.28502	0
Up	BAMBI	12	0.001425	712144	0.239316	0
Up	CARD14	12	4.10E-04	302240	0.267937	0
Up	PGGT1B	12	0.00145	932922	0.231124	0
Up	WDR89	12	0.001311	993054	0.279761	0
Up	ZNF354A	12	7.61E-04	923222	0.264906	0
Up	ATCAY	11	8.72E-05	62794	0.284573	0
Up	CCNL1	11	5.28E-04	302612	0.275448	0
Up	ABCC5	11	0.001451	872734	0.25738	0
Up	ZNF697	10	7.48E-04	634488	0.211517	0
Up	DMXL1	10	7.68E-04	308174	0.252423	0
Up	RAB33A	10	0.00112	563432	0.249538	0
Up	SAMD9	9	0.00117	600284	0.260007	0
Up	NCOA7	9	0.001101	730090	0.245352	0
Up	CD84	9	0.001033	728256	0.247908	0
Up	C11orf87	9	7.95E-04	411636	0.225532	0
Up	AGPAT4	9	0.001421	916616	0.209571	0
Up	ZNF430	8	9.80E-04	288378	0.276208	0.047619

Up	LILRB1	8	1.06E-05	3322	0.230437	0
Up	ZDHHC21	8	0.001463	1048744	0.245238	0
Up	CST7	8	0.001791	1344300	0.23254	0
Up	MAP9	8	1.30E-04	135846	0.270407	0
Up	ARHGAP15	7	6.81E-04	190748	0.282965	0
Up	NFXL1	7	4.55E-04	340848	0.261388	0
Up	HSD17B11	7	7.09E-04	597650	0.286313	0
Up	CITED4	7	3.47E-04	291144	0.250054	0
Up	SYTL3	6	6.89E-04	460710	0.147847	0
Up	PLCH1	6	7.78E-04	360000	0.252566	0
Up	FAM133B	6	1.13E-04	141062	0.279183	0
Up	SP140L	6	4.90E-04	239794	0.267567	0
Up	TUB	6	7.08E-04	608742	0.23649	0
Up	TRPC7	5	1	6	1	0
Up	ARHGAP27	5	8.53E-06	7612	0.247033	0
Up	DIRAS2	5	7.09E-04	349968	0.217891	0
Up	NAP1L2	5	6.89E-04	624774	0.25577	0
Up	RASGRP1	4	4.89E-04	312594	0.247507	0
Up	ZNF784	4	5.61E-05	34306	0.232671	0
Up	ZNF649	4	3.46E-04	150480	0.23282	0
Up	HSF5	4	1.16E-06	1088	0.238745	0
Up	STK31	4	3.49E-04	220646	0.240755	0
Up	OMD	4	6.92E-04	374446	0.214328	0
Up	LAD1	4	2.24E-05	23944	0.276946	0
Up	ZNF83	3	3.47E-04	166192	0.232699	0
Up	SLC18A2	3	0	0	0.243961	0
Up	DENND2C	3	6.89E-04	272418	0.216979	0
Up	C9orf139	3	6.89E-04	222890	0.198211	0
Up	CD3G	3	3.54E-04	352224	0.214526	0
Up	CRB1	3	3.45E-04	251236	0.224304	0
Up	CAPS	3	1.14E-06	920	0.240485	0

Up	STK17A	2	0	0	0	0
Up	MYRIP	2	0.001377	921400	0.209828	0
Up	C19orf12	2	9.71E-06	21804	0.274109	0
Up	FRMPD4	2	3.44E-04	183682	0.226253	0
Up	TNFRSF11B	2	1	2	1	0
Up	IFNW1	2	1.13E-05	4674	0.26584	0
Up	DPY19L2	2	0	0	1	0
Up	ZNF84	2	3.08E-05	32012	0.242009	0
Up	GSTT2	2	3.44E-04	196528	0.227654	0
Up	ABCB4	2	1	2	1	0
Up	SLC25A2	2	3.87E-05	51300	0.234144	0
Up	CCDC71L	2	2.46E-06	1790	0.230135	0
Up	KLRK1	2	3.44E-04	169280	0.213878	0
Up	ABCG5	1	0	0	1	0
Up	HPGD	1	0	0	0.263428	0
Up	IMMP2L	1	0	0	0.243603	0
Up	TCEAL5	1	0	0	1	0
Up	C2orf74	1	0	0	0.215978	0
Up	ZNF716	1	0	0	0.257334	0
Up	NT5C1B	1	0	0	0.257334	0
Up	ZNF804B	1	0	0	0.210849	0
Up	SLC44A3	1	0	0	0.202264	0
Up	FAM9C	1	0	0	0.23341	0
Up	ERICH1	1	0	0	0.251483	0
Up	WNT10A	1	0	0	0.251483	0
Up	TMEM74	1	0	0	1	0
Up	C12orf40	1	0	0	0.227618	0
Up	MFSD9	1	0	0	0.208271	0
Up	ZNF738	1	0	0	0.241165	0
Up	ZNF708	1	0	0	0.241165	0
Up	ABCA6	1	0	0	1	0

Up	MCTP2	1	0	0	0.263428	0
Up	SEMA3A	1	0	0	1	0
Up	SLC14A2	1	0	0	1	0
Up	CLEC2B	1	0	0	0.263428	0
Up	CHRDL2	1	0	0	1	0
Up	PRB2	1	0	0	0.221481	0
Up	INPP5F	1	0	0	0.242251	0
Up	LIPA	1	0	0	0.251407	0
Down	UBC	799	0.218173	75534956	0.392325	0.001503
Down	FN1	718	0.328615	48390654	0.437081	0.002231
Down	VHL	587	0.096408	18147816	0.375508	0.004368
Down	HSPA8	576	0.173669	48349874	0.395328	0.002184
Down	SOD1	245	0.071264	17465716	0.369713	0.003601
Down	RPL14	192	0.030814	8946280	0.349996	0.015146
Down	UBB	182	0.049252	14437930	0.340788	4.14E-04
Down	ALB	178	0.067014	7846552	0.360366	0.005123
Down	EEF1A1	168	0.031484	11944926	0.358741	0.011819
Down	RPL30	145	0.030272	8232074	0.351232	0.020591
Down	HSP90B1	129	0.029873	10084508	0.333054	0.002516
Down	FGL1	126	0.026325	4245640	0.277568	0
Down	RPL11	117	0.003725	2593220	0.327865	0.007212
Down	FGB	105	0.038168	4918076	0.32336	0.006535
Down	RPL3	100	0.012256	5200810	0.341696	0.022207
Down	RPLP0	96	0.012053	4254370	0.362105	0.027168
Down	CEP89	91	0.026356	7722906	0.287584	0
Down	ACTG1	90	0.017438	4988288	0.338152	0.006024
Down	MLLT1	87	0.012162	3785138	0.281438	0.003484
Down	EP400	82	0.009442	2816160	0.289257	0
Down	P4HB	81	0.015895	5344532	0.319617	0.001317
Down	RPL7A	79	0.006001	2911512	0.34317	0.020344
Down	RPL23A	78	0.006574	3734796	0.331776	0.005708

Down	ZFP41	77	0.015261	2911550	0.265116	0
Down	PCK1	76	0.017632	3349026	0.328298	0.012877
Down	APOA1	75	0.020417	2327144	0.328569	0.021445
Down	DKKL1	73	0.028521	6204926	0.276181	0
Down	FTH1	68	0.024473	9361246	0.288984	0.003365
Down	FAM83H	67	0.015605	4392044	0.3004	0
Down	BAIAP2	65	0.013242	3593002	0.296946	0
Down	APOB	63	0.014453	1708896	0.312117	0.005319
Down	ERRFI1	62	0.009875	1646064	0.334736	0.026713
Down	LDHA	62	0.010825	3691522	0.34252	0.013115
Down	KHSRP	52	0.006681	1947604	0.345106	0.028912
Down	RPS27	51	0.010973	3727378	0.342137	0.029804
Down	GNG2	50	0.006471	1757972	0.270122	0
Down	RPL31	49	0.004626	2305186	0.327865	0.005952
Down	PDGFRA	48	0.009778	2934856	0.291783	0
Down	DAB2IP	48	0.008034	2216604	0.299766	0
Down	KLC3	48	0.012742	3330684	0.287751	0
Down	RPL27A	47	0.003331	1562566	0.325823	0.008696
Down	RBM24	45	0.006223	773842	0.253006	0
Down	BANP	45	0.007271	4039232	0.282357	0
Down	RPL19	45	0.001291	1500970	0.297479	0
Down	SLC9A1	43	0.009547	3088208	0.284804	0
Down	DENND2D	42	0.008231	981960	0.297635	0.013333
Down	SERPINA1	42	0.005709	690648	0.332803	0.019943
Down	CIRBP	41	0.008543	2350782	0.328162	0.006098
Down	NPTX1	40	0.012349	1782322	0.265594	0
Down	B2M	38	0.011465	1248416	0.278054	0.002849
Down	TF	36	0.004799	788946	0.312559	0.036232
Down	HINT1	36	0.004757	826390	0.294048	0.01087
Down	ALDH2	34	0.005207	944738	0.291462	0.031579
Down	A2M	32	0.010034	1671224	0.272285	0

Down	PCK2	31	0.011663	1597368	0.286404	0.002151
Down	KLHL18	31	0.003175	1297108	0.28462	0
Down	COX1	30	0.008506	1200028	0.244423	0
Down	KNG1	29	0.005034	493356	0.303379	0.005848
Down	RPLP1	28	0.001403	1280332	0.287542	0
Down	PNMA2	28	0.008154	984518	0.270526	0
Down	ARG1	28	0.006842	3059490	0.291163	0
Down	CCL22	27	0.008589	1864888	0.27695	0
Down	CIITA	27	0.00443	720926	0.28106	0
Down	FGG	26	0.00212	325270	0.312977	0.156863
Down	ACAT1	26	0.001892	591856	0.304821	0
Down	COL18A1	26	0.004618	1556100	0.273446	0
Down	FTL	25	0.00331	1489940	0.266823	0.040936
Down	RPL34	25	0.001832	1118924	0.293462	0
Down	SPG7	25	0.009074	1421614	0.251916	0
Down	CPNE7	24	0.005904	1822996	0.290525	0.007246
Down	DHRS2	23	0.004226	2073968	0.274465	0
Down	RPS26	23	9.66E-04	398582	0.320855	0.019048
Down	FGA	22	0.002308	340098	0.31913	0.228758
Down	HSD17B6	22	0.003785	723898	0.201735	0
Down	C3	20	0.002646	404744	0.315585	0.058824
Down	IGFBP1	20	0.006948	1476680	0.246651	0
Down	DUSP26	20	0.00423	785754	0.267541	0
Down	TMEM108	19	0.006907	2799226	0.223905	0
Down	AKT3	19	0.00422	1204286	0.26868	0
Down	ST6GAL1	18	0.004924	654624	0.258532	0
Down	BCAM	18	0.005785	1052078	0.238816	0
Down	PEBP1	18	0.00137	584916	0.260975	0
Down	RFT1	18	0.003198	534138	0.258146	0
Down	TPT1	17	0.004633	634484	0.29668	0.030303
Down	ITIH2	17	0.007916	1300728	0.318109	0

Down	SSH2	17	0.001152	319308	0.279716	0
Down	CYP3A4	16	0.002573	309510	0.291911	0.027778
Down	DGKZ	16	0.002387	342038	0.293484	0.032967
Down	GC	16	0.002899	618272	0.259172	0
Down	ECHDC2	16	0.004029	658158	0.219857	0
Down	ERGIC1	16	0.003093	1235466	0.27339	0
Down	AHSG	15	0.001225	258590	0.29997	0.106061
Down	HP	15	0.00105	142288	0.282157	0.060606
Down	SERPINC1	15	0.002641	1033782	0.234895	0
Down	OBSCN	15	0.002788	1242502	0.264375	0
Down	PPM1F	15	0.00371	927348	0.291462	0
Down	RPS29	15	0.002258	1288178	0.274598	0
Down	PRNP	15	0.001569	306248	0.273333	0
Down	GSTA1	14	0.00261	355912	0.253086	0
Down	HK3	14	0.005035	674426	0.203813	0
Down	SCP2	14	0.002177	396108	0.267146	0
Down	CLEC18A	13	0.002071	234594	0.255657	0
Down	CDKN2B	13	0.001007	193134	0.234451	0
Down	DDIT4	12	0.001036	431588	0.274579	0
Down	CYB5A	12	0.002121	439134	0.25404	0
Down	APOA2	11	2.50E-04	25730	0.27071	0.222222
Down	RBP4	10	0.002682	220762	0.256763	0
Down	CFB	10	0.003805	298298	0.315485	0.071429
Down	TTR	10	0.001753	153542	0.277027	0.178571
Down	ORM1	10	5.68E-04	377228	0.231127	0.035714
Down	TBC1D16	10	0.002051	854746	0.249937	0
Down	ACE	9	7.28E-04	106694	0.266145	0
Down	NUDT8	9	0.00303	1479436	0.18667	0
Down	GSTA2	9	0.001031	164648	0.181234	0
Down	AMBP	8	0.00389	307314	0.31496	0.166667
Down	C1S	8	0.001367	149378	0.305125	0

Down	AKR1C1	8	0.002011	210268	0.304354	0
Down	CP	8	1.35E-04	45816	0.270305	0.142857
Down	TRIM49	8	5.37E-04	131368	0.271579	0
Down	GSTM4	8	0.001508	458352	0.22455	0
Down	PLG	7	2.72E-05	14336	0.273108	0
Down	BHMT	7	0.001508	727776	0.197047	0
Down	CYTB	7	0.001786	196606	0.241513	0
Down	C4BPA	7	0.001249	73972	0.309904	0.066667
Down	METTL7A	7	0.001933	269968	0.26089	0
Down	SEC61A2	7	0.001067	278594	0.245585	0
Down	SMR3B	7	0.001511	233078	0.250867	0
Down	TRIM72	7	6.27E-04	78990	0.249357	0
Down	ACAA2	7	0.002109	558152	0.251757	0
Down	AGT	6	0.001209	88060	0.258381	0
Down	ITIH3	6	0.002011	255276	0.18639	0
Down	MAT1A	6	5.03E-04	90244	0.230751	0
Down	F2	5	0.001341	116804	0.314387	0.3
Down	MT2A	5	5.17E-04	242292	0.226313	0
Down	LPA	5	2.05E-04	29092	0.251279	0
Down	COL4A6	5	2.72E-04	29418	0.273409	0
Down	SLC39A14	5	5.99E-04	93388	0.269957	0
Down	SPATA19	5	0.001061	320240	0.230404	0
Down	HEMK1	5	1	12	1	0
Down	CYP2C9	5	0.001508	110442	0.192237	0
Down	ANG	5	6.05E-06	1906	0.268934	0
Down	HRSP12	4	0.001064	212332	0.255772	0
Down	UGT2B7	4	0	0	0.225966	0
Down	ALDOB	4	0.001034	245666	0.222415	0
Down	EXOC3L2	4	0.001508	353658	0.283464	0
Down	RPL39	4	5.09E-04	64620	0.2543	0
Down	TMPRSS13	4	5.15E-04	88230	0.262664	0

Down	ZNF215	4	7.55E-05	11440	0.256846	0
Down	ADH1C	4	1	2	1	0
Down	B4GALT6	4	0.001006	126196	0.237646	0
Down	ARL11	4	0.001508	603546	0.193698	0
Down	PALM3	4	0.001508	387876	0.250741	0
Down	MGST1	4	5.15E-04	165834	0.256184	0
Down	REXO1	4	1.14E-05	2092	0.265647	0
Down	HRG	4	0.001006	261086	0.235158	0
Down	APOC3	3	5.03E-04	59976	0.268698	0.333333
Down	ITIH1	3	0.002512	184040	0.311922	0.333333
Down	ORM2	3	0	0	0.223678	1
Down	SLC2A6	3	0.001006	126714	0.182197	0
Down	CYSLTR2	3	0	0	1	0
Down	CFI	3	5.03E-04	191584	0.183204	0
Down	REG3A	3	1.05E-05	2712	0.24191	0
Down	APOH	3	6.17E-06	426	0.241998	0
Down	CFHR1	2	5.03E-04	45920	0.198354	0
Down	APOC1	2	0	0	0.268662	1
Down	CPB2	2	3.98E-05	8880	0.304331	0
Down	UGT1A6	2	0	0	0.225966	0
Down	CYP2E1	2	1	2	1	0
Down	ITIH4	2	8.58E-06	2144	0.304261	0
Down	CNGB1	2	5.03E-04	129650	0.214915	0
Down	COX7C	2	1	2	1	0
Down	PON3	2	1	2	1	0
Down	C22orf23	2	0	0	0.244965	0
Down	PAH	2	0	0	0	0
Down	DLX4	2	5.03E-04	57204	0.196444	0
Down	ALDH1A1	2	5.03E-04	146678	0.198097	0
Down	FABP1	2	3.43E-04	75008	0.215159	0
Down	MYOT	2	5.03E-04	52570	0.233324	0

Down	TMC1	2	3.20E-07	630	0.267775	0
Down	CFHR2	1	0	0	0.165529	0
Down	MT1X	1	0	0	0.251073	0
Down	IGF2	1	0	0	0.197861	0
Down	APOC2	1	0	0	0.264921	0
Down	HPR	1	0	0	0.198315	0
Down	CECR1	1	0	0	0.214105	0
Down	STK32B	1	0	0	0.253732	0
Down	MT1E	1	0	0	0.245433	0
Down	UGT2B15	1	0	0	1	0
Down	UPK3A	1	0	0	1	0
Down	MT1M	1	0	0	0.238115	0
Down	ACRBP	1	0	0	1	0
Down	FAM153C	1	0	0	0.202701	0
Down	STK32A	1	0	0	0.18632	0
Down	SYT14	1	0	0	1	0
Down	IL23R	1	0	0	0.173486	0
Down	MN1	1	0	0	0.179914	0
Down	C4B	1	0	0	0.265736	0
Down	GATM	1	0	0	0.193509	0
Down	NNMT	1	0	0	0.248531	0
Down	KCNH1	1	0	0	1	0
Down	MYF5	1	0	0	1	0
Down	OLIG2	1	0	0	0.214892	0
Down	ADH4	1	0	0	0.250646	0

Table 7 miRNA - target gene interaction table

Regulation	Target Genes	Degree	MicroRNA	Regulation	Target Genes	Degree	MicroRNA
Up	TAOK1	222	hsa-mir-3941	Down	KHSRP	178	hsa-mir-548ac
Up	HMGB1	143	hsa-mir-1183	Down	TRIM72	123	hsa-mir-6890-3p

Up	ZNF708	104	hsa-mir-6832-5p	Down	MLLT1	95	hsa-mir-5681a
Up	MYC	103	hsa-mir-4662b	Down	C3	93	hsa-mir-3135b
Up	ZNF101	91	hsa-mir-3187-3p	Down	DDIT4	82	hsa-mir-3607-3p
Up	CCDC71L	80	hsa-mir-4749-3p	Down	RFT1	73	hsa-mir-6884-5p
Up	LYRM4	80	hsa-mir-3652	Down	CCL22	71	hsa-mir-1273h-5p
Up	ARF1	80	hsa-mir-6513-3p	Down	NPTX1	69	hsa-mir-4795-3p
Up	IRAK4	77	hsa-mir-4327	Down	ENTHD1	67	hsa-mir-1470
Up	UBE2D3	76	hsa-mir-4459	Down	EEF1A1	62	hsa-mir-4459
Up	JARID2	76	hsa-mir-5583-3p	Down	PDGFRA	61	hsa-mir-1827
Up	BAMBI	76	hsa-mir-6889-3p	Down	HINT1	61	hsa-mir-6513-5p
Up	SYAP1	76	hsa-mir-6787-3p	Down	PPM1F	60	hsa-mir-6817-5p
Up	NARS	75	hsa-mir-4446-5p	Down	RPL14	58	hsa-mir-3960
Up	ZDHHC21	73	hsa-mir-1271-3p	Down	PRNP	57	hsa-mir-4798-3p
Up	TRIM37	71	hsa-mir-4255	Down	ERGIC1	56	hsa-mir-1228-3p
Up	NRBF2	69	hsa-mir-548aq-3p	Down	ANKRD45	55	hsa-mir-6760-5p
Up	FAM9C	64	hsa-mir-4427	Down	HSPA8	53	hsa-mir-6801-3p
Up	MFSD9	61	hsa-mir-548l	Down	G6PC	53	hsa-mir-365b-3p
Up	SPOP	61	hsa-mir-1236-3p	Down	EIF1	52	hsa-mir-6076
Up	SATB2	60	hsa-mir-4782-3p	Down	PNMA2	52	hsa-mir-7159-5p
Up	RBM8A	60	hsa-mir-3689c	Down	HSP90B1	51	hsa-mir-4419b
Up	LSM3	60	hsa-mir-4768-5p	Down	CEP89	51	hsa-mir-6504-3p
Up	MYO10	58	hsa-mir-5011-3p	Down	ACTG1	49	hsa-mir-4776-3p
Up	CAPRIN1	57	hsa-mir-6845-3p	Down	F2	46	hsa-mir-1260b
Up	PRNP	57	hsa-mir-7-2-3p	Down	AKT3	46	hsa-mir-6891-3p
Up	ABCC5	52	hsa-mir-4741	Down	TACR3	46	hsa-mir-4528
Up	LIPA	52	hsa-mir-5047	Down	IGF2	45	hsa-mir-6891-5p
Up	HSP90B1	51	hsa-mir-1827	Down	ANG	44	hsa-mir-3922-5p
Up	PDCD10	50	hsa-mir-1277-5p	Down	LDHA	43	hsa-mir-8080
Up	ZNF83	50	hsa-mir-6809-3p	Down	REXO1	43	hsa-mir-1180-5p
Up	ATM	49	hsa-mir-7151-3p	Down	SSH2	43	hsa-mir-4780
Up	LSG1	47	hsa-mir-6858-3p	Down	MAT1A	43	hsa-mir-7111-5p
Up	EOGT	47	hsa-mir-301a-3p	Down	RPL27A	42	hsa-mir-7703
Up	CDC42SE1	46	hsa-mir-4530	Down	APOH	42	hsa-mir-302a-3p
Up	AKT3	46	hsa-mir-3976	Down	UBB	42	hsa-mir-3666

Up	MIXL1	43	hsa-mir-4252	Down	B2M	41	hsa-mir-526b-3p
Up	ATCAY	42	hsa-mir-4485-5p	Down	MT1A	41	hsa-mir-3944-5p
Up	DDX21	40	hsa-mir-548x-3p	Down	CYB5A	40	hsa-mir-600
Up	ZNF716	40	hsa-mir-1200	Down	METTL7A	39	hsa-mir-6895-3p
Up	HNRNPDL	39	hsa-mir-377-5p	Down	FAM83H	39	hsa-mir-5582-5p
Up	ARPC3	39	hsa-mir-5194	Down	UBOX5	38	hsa-mir-520c-3p
Up	PPIG	38	hsa-mir-6507-5p	Down	VHL	38	hsa-mir-6802-3p
Up	CLEC2D	38	hsa-mir-6818-3p	Down	PEBP1	38	hsa-mir-548an
Up	ODF2L	37	hsa-mir-4506	Down	APOC3	37	hsa-mir-4421
Up	NPM1	37	hsa-mir-4468	Down	RPLP0	36	hsa-mir-664a-5p
Up	NPEPPS	37	hsa-mir-500b-3p	Down	ACAA2	34	hsa-mir-6780a-5p
Up	DDX52	36	hsa-mir-4722-5p	Down	SLC29A2	34	hsa-mir-4756-5p
Up	DMXL1	36	hsa-mir-1273f	Down	SERPINC1	32	hsa-mir-6845-3p
Up	ESF1	34	hsa-mir-1208	Down	SLC9A1	30	hsa-mir-5196-5p
Up	RASL10B	34	hsa-mir-3612	Down	TPT1	29	hsa-mir-3200-3p
Up	EML4	33	hsa-let-7f-2-3p	Down	CECR1	29	hsa-mir-520f-3p
Up	CHD7	33	hsa-mir-548u	Down	KCNH1	28	hsa-mir-2467-3p
Up	RPS14	33	hsa-mir-4477a	Down	PCK1	25	hsa-mir-548aa
Up	CD84	33	hsa-mir-4763-3p	Down	RPS27	24	hsa-mir-6088
Up	SYTL3	31	hsa-mir-3689a-3p	Down	RPLP1	24	hsa-mir-5010-3p
Up	STK17A	31	hsa-mir-6817-3p	Down	P4HB	24	hsa-mir-378d
Up	NUCB2	30	hsa-mir-5586-3p	Down	SAA1	24	hsa-mir-4792
Up	NEDD4L	30	hsa-mir-4710	Down	RPL34	24	hsa-mir-1304-3p
Up	KRR1	30	hsa-mir-6124	Down	CIITA	24	hsa-mir-4441
Up	NAPG	29	hsa-mir-499a-3p	Down	SLC39A14	23	hsa-mir-3653-5p
Up	CCT6A	29	hsa-mir-4789-5p	Down	FTH1	23	hsa-mir-4790-3p
Up	ABCA6	28	hsa-mir-4430	Down	IL23R	23	hsa-mir-3666
Up	ZNF84	28	hsa-mir-6760-3p	Down	TTR	23	hsa-mir-6872-3p
Up	TRIM56	28	hsa-mir-6501-5p	Down	SLC35E4	23	hsa-mir-6819-3p
Up	AGPAT4	28	hsa-mir-6849-3p	Down	RPL30	22	hsa-mir-557
Up	PGGT1B	27	hsa-mir-4282	Down	CYSLTR2	22	hsa-mir-9500
Up	OMD	26	hsa-mir-3666	Down	ERRFI1	21	hsa-mir-4495
Up	CITED4	26	hsa-mir-1197	Down	RPS26	21	hsa-mir-519e-5p
Up	BAG5	25	hsa-mir-4260	Down	FGG	21	hsa-mir-6765-5p

Up	SLFN13	25	hsa-mir-1207-3p	Down	B4GALT6	19	hsa-mir-1248
Up	GNL3	25	hsa-mir-5692c	Down	FGB	18	hsa-mir-5587-5p
Up	GLRX	24	hsa-mir-6745	Down	CACFD1	18	hsa-mir-1207-5p
Up	CCNL1	24	hsa-mir-6735-5p	Down	RPL3	17	hsa-mir-1296-5p
Up	ZNF430	24	hsa-mir-4772-3p	Down	MN1	17	hsa-mir-548u
Up	DCUN1D2	24	hsa-mir-6843-3p	Down	KLF17	17	hsa-mir-4284
Up	ARHGAP15	24	hsa-mir-6807-5p	Down	TBC1D16	17	hsa-mir-6867-5p
Up	FRMPD4	24	hsa-mir-1268a	Down	ZNF215	17	hsa-mir-5572
Up	DTHD1	24	hsa-mir-7111-3p	Down	SEC61A2	16	hsa-mir-7158-3p
Up	ZNF736	24	hsa-mir-4310	Down	KIAA1324	16	hsa-mir-548x-3p
Up	TPM1	22	hsa-mir-6831-3p	Down	BANP	16	hsa-mir-888-5p
Up	HIST1H1C	22	hsa-mir-3940-5p	Down	BCAM	16	hsa-mir-6795-5p
Up	DIRAS2	22	hsa-mir-7109-5p	Down	HEMK1	16	hsa-mir-548p
Up	ARGLU1	21	hsa-mir-4463	Down	ADH4	16	hsa-mir-4666a-5p
Up	TERF1	21	hsa-mir-3120-5p	Down	SLC38A4	16	hsa-mir-4763-3p
Up	CCDC47	21	hsa-mir-500a-3p	Down	COL18A1	15	hsa-mir-8059
Up	C19orf12	21	hsa-mir-6822-5p	Down	CDKN2B	15	hsa-mir-4792
Up	TRIM38	20	hsa-mir-600	Down	CNGB1	15	hsa-mir-7158-3p
Up	TNFAIP8	20	hsa-mir-6867-5p	Down	APOA1	15	hsa-mir-1305
Up	NELL2	20	hsa-mir-6810-3p	Down	C9orf47	15	hsa-mir-4684-5p
Up	FAM49B	19	hsa-mir-4291	Down	UBC	14	hsa-mir-769-3p
Up	WNT10A	19	hsa-mir-4690-3p	Down	SCP2	14	hsa-mir-4703-3p
Up	NT5C1B	19	hsa-mir-6778-3p	Down	KLHL18	14	hsa-mir-5191
Up	MDFI	19	hsa-mir-1470	Down	GATM	14	hsa-mir-6830-3p
Up	TENM3	18	hsa-mir-660-3p	Down	RPL7A	13	hsa-mir-1303
Up	MTR	18	hsa-mir-548ax	Down	ST6GAL1	13	hsa-mir-5009-3p
Up	DUSP9	18	hsa-mir-1321	Down	ARL11	13	hsa-mir-3662
Up	CHMP2B	18	hsa-mir-4697-3p	Down	ALDH2	12	hsa-mir-6790-3p
Up	CD96	18	hsa-mir-6821-3p	Down	HLA-C	12	hsa-mir-6864-3p
Up	GCLM	17	hsa-mir-5587-5p	Down	RPL23A	12	hsa-mir-892c-5p
Up	TUB	17	hsa-mir-374c-3p	Down	MT2A	11	hsa-let-7g-3p
Up	IFNGR1	17	hsa-mir-3919	Down	MT1E	11	hsa-mir-4668-3p
Up	NCOA7	17	hsa-mir-4789-5p	Down	SLC2A6	11	hsa-mir-4701-5p
Up	STX2	17	hsa-mir-6089	Down	NNMT	11	hsa-mir-6086

Up	CACYBP	17	hsa-mir-3147	Down	GSTM4	10	hsa-mir-3135a
Up	UTRN	16	hsa-mir-3136-3p	Down	RPL19	10	hsa-mir-5197-5p
Up	INPP5F	15	hsa-mir-519d-3p	Down	HPR	10	hsa-mir-4290
Up	RASD1	15	hsa-mir-3180-3p	Down	APOB	10	hsa-mir-6869-5p
Up	ZNF283	15	hsa-mir-6778-3p	Down	DLX4	9	hsa-mir-6819-3p
Up	MARS	14	hsa-mir-541-5p	Down	GNG2	9	hsa-mir-6867-3p
Up	RPL5	14	hsa-mir-1206	Down	FUT6	9	hsa-mir-7845-5p
Up	IMMP2L	14	hsa-mir-5195-3p	Down	SEMA3D	9	hsa-mir-2053
Up	DENND2C	14	hsa-mir-5190	Down	UGT2B10	9	hsa-mir-7977
Up	SP140L	14	hsa-mir-4684-5p	Down	IGFBP1	8	hsa-mir-5580-3p
Up	LILRB1	14	hsa-mir-5011-5p	Down	ASCL5	8	hsa-mir-4691-5p
Up	IBTK	13	hsa-mir-140-3p	Down	RPL31	7	hsa-let-7d-5p
Up	RASGRP1	13	hsa-mir-4677-3p	Down	EP400	7	hsa-let-7i-5p
Up	MIS18BP1	13	hsa-mir-320e	Down	FN1	7	hsa-let-7g-5p
Up	PABPC3	13	hsa-mir-3973	Down	C1S	7	hsa-mir-548s
Up	RASSF5	12	hsa-mir-19a-3p	Down	RBM24	7	hsa-mir-3168
Up	FANCD2	11	hsa-let-7c-3p	Down	DAB2IP	6	hsa-mir-338-3p
Up	DNAJC19	11	hsa-mir-370-5p	Down	ADAMTS15	6	hsa-mir-6723-5p
Up	ARHGAP27	11	hsa-mir-1199-3p	Down	FGA	5	hsa-mir-409-3p
Up	NRP1	10	hsa-mir-320a	Down	ARG1	4	hsa-mir-7-5p
Up	IFT74	10	hsa-mir-6071	Down	ACAT1	4	hsa-mir-23b-3p
Up	CLNS1A	10	hsa-mir-3142	Down	RPL39	4	hsa-mir-30c-5p
Up	PPIL2	10	hsa-mir-6507-5p	Down	RPS29	4	hsa-mir-10a-5p
Up	ITK	10	hsa-mir-3074-3p	Down	COX7C	4	hsa-mir-331-3p
Up	CEP70	10	hsa-mir-1470	Down	CYP2E1	4	hsa-mir-488-3p
Up	BCL6	9	hsa-mir-21-5p	Down	CCDC62	4	hsa-mir-3161
Up	ZCRB1	9	hsa-mir-30e-5p	Down	KLC3	4	hsa-mir-6809-3p
Up	KCTD6	9	hsa-mir-30d-3p	Down	HRSP12	3	hsa-mir-15a-5p
Up	ITGB3BP	9	hsa-mir-299-3p	Down	PCK2	3	hsa-mir-155-5p
Up	SLC25A2	9	hsa-mir-3190-5p	Down	CFI	3	hsa-mir-181a-5p
Up	STXBP3	8	hsa-mir-1-1	Down	DGKZ	3	hsa-mir-218-5p
Up	ENTPD6	8	hsa-mir-365a-5p	Down	A2M	3	hsa-mir-122-5p
Up	ID1	8	hsa-mir-100-5p	Down	MT1F	3	hsa-mir-219a-5p
Up	CRB1	8	hsa-mir-18a-5p	Down	SOD1	3	hsa-mir-378a-3p

Up	NFE2L2	8	hsa-mir-106b-3p	Down	PAH	3	hsa-mir-590-3p
Up	GCC2	8	hsa-mir-4422	Down	NUDT8	2	hsa-let-7e-5p
Up	ROBO2	8	hsa-mir-4801	Down	FTL	2	hsa-mir-133a-3p
Up	TMEM92	8	hsa-mir-2116-3p	Down	TF	2	hsa-mir-19a-3p
Up	KCNAB1	8	hsa-mir-3606-3p	Down	CIRBP	2	hsa-mir-30a-5p
Up	KCNJ4	8	hsa-mir-3667-3p	Down	HGD	2	hsa-mir-26b-5p
Up	HES1	7	hsa-mir-296-3p	Down	MPC1	2	hsa-mir-331-3p
Up	PPA1	7	hsa-mir-200a-5p	Down	RPL11	2	hsa-mir-505-5p
Up	WDR89	7	hsa-mir-20a-5p	Down	MGST1	2	hsa-mir-652-3p
Up	SCAF11	7	hsa-mir-29c-3p	Down	CFB	2	hsa-mir-98-5p
Up	ZNF784	7	hsa-mir-1306-5p	Down	COL4A6	2	hsa-mir-29b-3p
Up	COMMD6	7	hsa-mir-1298-5p	Down	MT1X	2	hsa-mir-1225-3p
Up	ABCG5	7	hsa-mir-4753-3p	Down	MYF5	2	hsa-mir-10b-5p
Up	HPGD	6	hsa-mir-4289	Down	ALDOB	2	hsa-mir-378a-3p
Up	ITGAV	6	hsa-mir-196a-5p	Down	SYT14	2	hsa-mir-132-3p
Up	LEF1	6	hsa-mir-449a	Down	SEPP1	2	hsa-mir-148b-3p
Up	ZNF649	6	hsa-mir-4313	Down	DUSP26	2	hsa-mir-544a
Up	HSF5	6	hsa-mir-4733-5p	Down	KRTAP3-3	2	hsa-mir-148b-3p
Up	UTP14A	5	hsa-mir-27b-3p	Down	PSORS1C2	2	hsa-mir-1468-5p
Up	PSMA3	5	hsa-mir-147a	Down	ADAM29	1	hsa-mir-26b-5p
Up	MIF	5	hsa-mir-451a	Down	AGT	1	hsa-mir-26b-5p
Up	MAP9	5	hsa-mir-548m	Down	AOX1	1	hsa-mir-26b-5p
Up	HSPH1	4	hsa-mir-16-5p	Down	CPB2	1	hsa-mir-26b-5p
Up	ACTL8	4	hsa-mir-148b-5p	Down	GSTA1	1	hsa-mir-26b-5p
Up	SERPINB9	4	hsa-mir-9-5p	Down	ITGAX	1	hsa-mir-26b-5p
Up	MPP1	4	hsa-mir-32-5p	Down	MT1M	1	hsa-mir-26b-5p
Up	PTPN2	4	hsa-mir-210-3p	Down	TMEM108	1	hsa-mir-183-5p
Up	CHD2	4	hsa-mir-378a-5p	Down	CPNE7	1	hsa-mir-214-3p
Up	C12orf40	4	hsa-mir-148b-3p	Down	OLIG2	1	hsa-mir-218-5p
Up	CCDC91	4	hsa-mir-5689	Down	CYP3A4	1	hsa-mir-27b-3p
Up	C11orf87	4	hsa-mir-4520-3p	Down	ZSCAN4	1	hsa-mir-122-5p
Up	RBM34	3	hsa-mir-30a-5p	Down	DENND2D	1	hsa-mir-124-3p
Up	IFI30	3	hsa-let-7c-3p	Down	HSD17B6	1	hsa-mir-124-3p
Up	TNFRSF11B	3	hsa-mir-181a-5p	Down	KNG1	1	hsa-mir-128-3p

Up	HIST4H4	3	hsa-mir-615-3p	Down	ADH1A	1	hsa-mir-132-3p
Up	TRIP6	3	hsa-mir-484	Down	ADH1C	1	hsa-mir-132-3p
Up	CAPN2	3	hsa-mir-101-3p	Down	ALDH1A1	1	hsa-mir-140-5p
Up	NFIL3	3	hsa-mir-98-5p	Down	LGI2	1	hsa-mir-149-5p
Up	KLRK1	3	hsa-mir-9-5p	Down	OBSCN	1	hsa-mir-155-5p
Up	SLC18A2	3	hsa-mir-6507-5p	Down	ITIH4	1	hsa-mir-375
Up	CPEB1	2	hsa-let-7b-5p	Down	ACE	1	hsa-mir-335-5p
Up	C2orf74	2	hsa-mir-15a-5p	Down	APOC1	1	hsa-mir-335-5p
Up	NOL7	2	hsa-mir-16-5p	Down	AQP10	1	hsa-mir-335-5p
Up	ARL4A	2	hsa-mir-382-5p	Down	CFHR1	1	hsa-mir-335-5p
Up	CARD14	2	hsa-mir-26b-5p	Down	CFHR2	1	hsa-mir-335-5p
Up	HSD17B11	2	hsa-mir-1-3p	Down	CPO	1	hsa-mir-335-5p
Up	STX3	2	hsa-mir-375	Down	FABP1	1	hsa-mir-335-5p
Up	HPS1	2	hsa-mir-331-3p	Down	GC	1	hsa-mir-335-5p
Up	TIMM17B	2	hsa-mir-3176	Down	ITIH1	1	hsa-mir-335-5p
Up	ARHGEF10	2	hsa-mir-192-5p	Down	MYOT	1	hsa-mir-335-5p
Up	DBF4B	2	hsa-mir-215-5p	Down	ORM2	1	hsa-mir-335-5p
Up	ZNF354A	2	hsa-mir-197-3p	Down	PON3	1	hsa-mir-335-5p
Up	GLS	2	hsa-mir-7-5p	Down	PSORS1C1	1	hsa-mir-335-5p
Up	HSD17B3	2	hsa-mir-181a-5p	Down	SERPINA1	1	hsa-mir-335-5p
Up	PHLDA1	2	hsa-mir-375	Down	TMPRSS13	1	hsa-mir-335-5p
Up	CTNNAL1	2	hsa-mir-218-5p	Down	ZFP41	1	hsa-mir-335-5p
Up	MARCH11	2	hsa-mir-124-3p	Down	ECHDC2	1	hsa-mir-484
Up	NAP1L2	2	hsa-mir-369-3p	Down	ALB	1	hsa-mir-492
Up	ZSWIM8	2	hsa-mir-149-5p	Down	SMR3B	1	hsa-mir-495-3p
Up	CASP4	1	hsa-mir-26b-5p	Down	RASAL1	1	hsa-mir-421
Up	CLEC2B	1	hsa-mir-26b-5p	Down	BAIAP2	1	hsa-mir-455-3p
Up	GSTT2	1	hsa-mir-26b-5p	Down	LRRC9	1	hsa-mir-552-5p
Up	MCTP2	1	hsa-mir-26b-5p				
Up	PSMC6	1	hsa-mir-26b-5p				
Up	TRPC7	1	hsa-mir-26b-5p				
Up	SAMD9	1	hsa-mir-30a-5p				
Up	S100A10	1	hsa-mir-100-5p				
Up	NOP58	1	hsa-mir-221-3p				

Up	TMEM74	1	hsa-mir-122-5p
Up	PRB2	1	hsa-mir-124-3p
Up	TRIM7	1	hsa-mir-124-3p
Up	SDF2	1	hsa-mir-132-3p
Up	PFDN4	1	hsa-mir-155-5p
Up	ZNF493	1	hsa-mir-155-5p
Up	CBWD3	1	hsa-mir-375
Up	CHGB	1	hsa-mir-375
Up	ATP5H	1	hsa-mir-324-5p
Up	CHRDL2	1	hsa-mir-335-5p
Up	DPY19L2	1	hsa-mir-335-5p
Up	IFT81	1	hsa-mir-335-5p
Up	KCTD17	1	hsa-mir-335-5p
Up	MRGPRX3	1	hsa-mir-335-5p
Up	SPATA6L	1	hsa-mir-335-5p
Up	UNC5CL	1	hsa-mir-335-5p
Up	ZNF804B	1	hsa-mir-335-5p
Up	ERICH1	1	hsa-mir-92b-3p
Up	CHMP4C	1	hsa-mir-30c-2-3p
Up	NFXL1	1	hsa-mir-500a-5p
Up	TCEAL5	1	hsa-mir-2115-3p

Degree – No of miRNA interact with target gene. We taken any one miRNA in table.

Table 8 TF - target gene interaction table

Regulation	TF	Degree	Target Gene	Regulation	TF	Degree	Target Gene
Up	FOXC1	166	HSD17B11	Down	FOXC1	149	PAH
Up	GATA2	128	HEPACAM2	Down	GATA2	118	CYP3A4
Up	FOXL1	88	CACYBP	Down	YY1	76	CP
Up	YY1	76	NAPG	Down	SRF	66	COX7C
Up	USF2	59	PSMA3	Down	FOXL1	59	TTTY10
Up	NFIC	58	ABCC5	Down	NFKB1	57	APOC1
Up	PPARG	56	LILRB1	Down	NFIC	53	HSPA8
Up	JUN	54	STK17A	Down	PPARG	52	VHL

Up	NFKB1	54	SPX	Down	USF2	47	TTR
Up	E2F1	52	BCL6	Down	HINFP	46	CYP2E1
Up	POU2F2	51	IFNW1	Down	JUN	44	CFI
Up	CREB1	50	EML4	Down	E2F1	42	RPL11
Up	SRF	49	COMMD6	Down	RELA	42	ERGIC1
Up	NFYA	47	NEDD4L	Down	TFAP2A	41	EXOC3L2
Up	TP53	46	SYTL3	Down	CREB1	39	EEF1A1
Up	MEF2A	44	PPIG	Down	MEF2A	39	EP400
Up	STAT3	44	CCDC91	Down	TP53	39	FTL
Up	HINFP	41	MYO10	Down	STAT3	38	DAB2IP
Up	HOXA5	40	TNFRSF11B	Down	GATA3	37	FGL1
Up	TFAP2A	40	JARID2	Down	POU2F2	36	REXO1
Up	GATA3	39	FANCD2	Down	NFYA	36	ANG
Up	RELA	39	NPEPPS	Down	SREBF1	36	IL23R
Up	SREBF1	34	TUB	Down	HNF4A	33	SPATA19
Up	MAX	30	WNT10A	Down	STAT1	30	AKR1C1
Up	PAX2	30	DPY19L2	Down	ELK1	30	ARL11
Up	JUND	29	CAPN2	Down	JUND	28	C4BPA
Up	STAT1	29	MARS	Down	MAX	27	DLX4
Up	HNF4A	28	TBC1D3	Down	HOXA5	26	HP
Up	TEAD1	25	RASL10B	Down	PRDM1	24	CYB5A
Up	ELK1	25	LSM3	Down	SREBF2	24	UBC
Up	RUNX2	25	UTRN	Down	ARID3A	22	LRRC9
Up	IRF2	24	TPM1	Down	BRCA1	22	KLC3
Up	PRRX2	23	KLRK1	Down	IRF2	21	HLA-C
Up	CEBPB	23	HMGB1	Down	NR3C1	21	C1S
Up	USF1	22	CST7	Down	FOS	20	FGG
Up	FOS	21	ODF2L	Down	TEAD1	20	A2M
Up	FOXA1	21	NUCB2	Down	ESR1	20	UBB
Up	SREBF2	21	ARHGAP27	Down	E2F6	18	CACFD1
Up	ARID3A	20	RAB33A	Down	KLF5	18	FUT6
Up	PRDM1	19	ITK	Down	USF1	18	AOX1
Up	NRF1	19	MYRIP	Down	ELK4	18	TMPRSS13
Up	BRCA1	18	ATM	Down	ZNF354C	17	HINT1

Up	E2F6	18	ATP5H	Down	FOXF2	17	RPS27
Up	ELK4	18	HPGD	Down	FOXA1	17	APOB
Up	TP63	18	MRGPRX3	Down	TFAP2C	16	DDIT4
Up	TFAP2C	17	MYC	Down	TP63	16	HRG
Up	FOXF2	17	SPOP	Down	SPIB	15	LDHA
Up	NR3C1	17	NOP58	Down	PRRX2	15	MN1
Up	ZNF354C	16	DIRAS2	Down	CEBPB	15	AHSG
Up	NR2F1	14	MIF	Down	SP1	14	ACE
Up	ESR1	14	CEACAM19	Down	NR2F1	13	GC
Up	NKX3-2	13	IRAK4	Down	NKX3-2	12	BAIAP2
Up	PDX1	12	BAG5	Down	RUNX2	12	RFT1
Up	SRY	11	OMD	Down	NRF1	12	MT1A
Up	SP1	10	HES1	Down	SOX10	11	CEP89
Up	KLF5	10	CLNS1A	Down	EGR1	10	FN1
Up	EN1	9	ZDHHC21	Down	PAX2	9	ALDOB
Up	EGR1	8	NFIL3	Down	SOX5	9	UBOX5
Up	SPIB	7	STXBP3	Down	SOX17	9	SCP2
Up	SOX17	7	NCOA7	Down	PDX1	8	TF
Up	NKX2-5	6	HSPH1	Down	EN1	8	KRTAP3-3
Up	SOX5	6	PGGT1B	Down	NKX2-5	7	RPL27A
Up	SOX10	5	SDF2	Down	SRY	6	TACR3
Up	ELF5	5	EOGT	Down	FEV	4	SERPINA1
Up	FOXI1	4	TERF1	Down	NKX3-1	4	KIAA1324
Up	E2F4	4	ARGLU1	Down	REL	4	TRIM49
Up	ARNT	4	ATCAY	Down	FOXD1	3	FGA
Up	GATA1	4	CLNS1A	Down	ESRRB	3	TMEM108
Up	NFATC2	3	CD3G	Down	MYB	3	REG3A
Up	HNF1B	3	SATB2	Down	ELF5	3	ZFP41
Up	MYB	3	PDCD10	Down	E2F4	2	COL18A1
Up	REL	2	CXORF65	Down	PBX1	1	UGT2B11
Up	NR4A2	1	HIST4H4	Down	ESR2	1	CCL22
Up	FEV	1	CASP4	Down	SOX9	1	SLC35E4
Up	NKX3-1	1	UBE2D3	Down	FOXO3	1	PLG
Up	NOBOX	1	LYRM4	Down	FOXI1	1	DGKZ

Up	KLF4	1	NAPG	Down	NOBOX	1	PDGFRA
Up	NR2E3	1	HIST1H1C	Down	NFATC2	1	OR2H2
Up	NFYB	1	ID1	Down	ARNT	1	DGKZ
				Down	ZFX	1	SEPP1
				Down	NR2E3	1	KNG1
				Down	GATA1	1	ALDH2
				Down	HNF1B	1	BANP
				Down	NFYB	1	FTH1

Degree – No of target gene interact with TF. We taken any one target gene in table. TF transcription factors
